# Entropic Control of the Helicity Inversion Rates of Twisted Metallomacrocycles by Reversible and Regioselective Deprotonation

**DOI:** 10.1021/jacs.5c18601

**Published:** 2026-01-28

**Authors:** Tomoki Nakajima, Shohei Tashiro, Masahiro Ehara, Mitsuhiko Shionoya

**Affiliations:** † Department of Chemistry, Graduate School of Science, 13143 The University of Tokyo, 7-3-1 Hongo, Bunkyo-ku, Tokyo 113-0033, Japan; ‡ Research Centre for Computational Science, 88301 Institute for Molecular Science, Myodaiji, Okazaki, Aichi 444-8585, Japan; § Research Institute for Science and Technology, Tokyo University of Science 2641 Yamazaki, Noda, Chiba 278-8510, Japan

## Abstract

The rate of molecular motion has been enthalpically controlled by external stimuli such as acids and bases, electrons, and light. However, controlling the rate of molecular motion through activation entropy remains a challenging task. Here, we report entropic control of the helicity inversion rate of a trinuclear Pd^II^ macrocycle with right- and left-handed twisted structures. Three of the six NH protons in this metallo-macrocycle were regioselectively deprotonated by a moderately strong base, inducing intramolecular proton transfer from the NH to N^–^ moieties in the helicity inversion. After the partial deprotonation, the helicity inversion rate of the twisted macrocycle decreased to 1/20 of that before deprotonation due to the dominant influence of the activation entropy term. The kinetic isotope effects on the inversion rate suggest that an orderly proton relay occurs between the NH and N^–^ moieties via multiple water molecules during the inversion process, resulting in a significant reduction in the activation entropy. The mechanism by which the activation entropy term controls the helicity inversion rate via a proton relay is expected to provide a guide for the design of more advanced molecular machines.

## Graphical abstract

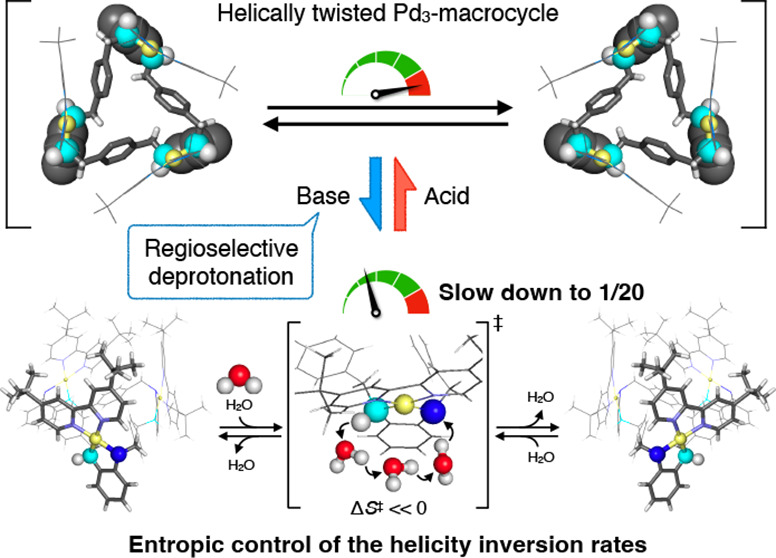

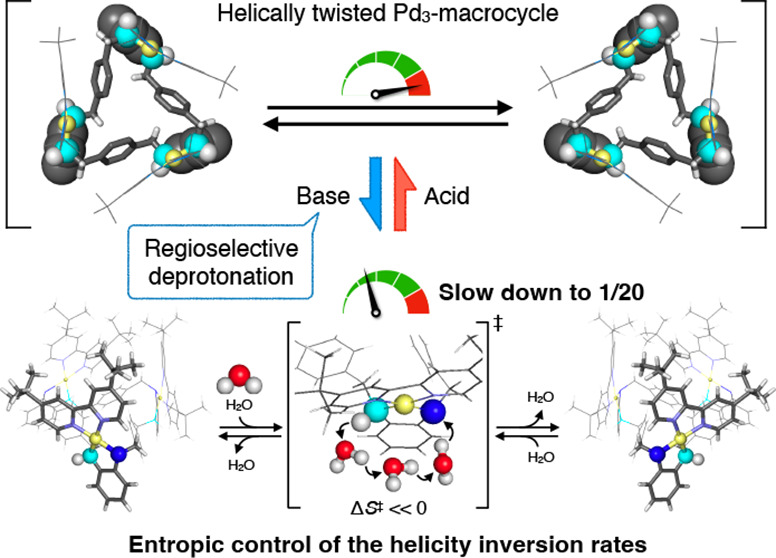

## Introduction

The translational and rotational motions of nanometer-sized molecules can be controlled by various external stimuli such as acids and bases,^1–5^ temperature,^6,7^ electrons,^8–11^ and light.^12–16^ Such molecular motions are driven by adjusting the ground-state Gibbs free energy, the enthalpy terms of which in most cases are modulated by steric effects, metal coordination, and noncovalent interactions including hydrogen bonding and electrostatic interactions/repulsions. On the other hand, the motion of molecules can also be controlled by entropy terms, as shown by the equation Δ*G* = Δ*H* – *T*Δ*S*. For example, Leigh et al. controlled the position of a synthetic molecular shuttle via the entropy term by varying the temperature.^6^ Although there are many examples of DNA nanomachines controlled by entropy terms,^17–21^ there are limited examples of artificial molecular systems.^6,7^

In contrast, the rate or frequency of molecular motions is determined by the Gibbs free energy of activation (Δ*G* ^‡^ = Δ*H* ^‡^ – *T*Δ*S* ^‡^) in the transition state. Furthermore, the main approaches include the modulation of the activation enthalpy terms (Δ*H* ^‡^) through electrostatic forces,^22,23^ steric effects,^24–27^ etc.^28–30^ For example, Wang et al. exploited the switching between attractive and repulsive interactions by the addition of an acid and a base^22^ to reversibly control the rate of molecular rotors by tuning the activation enthalpy term. However, activation entropy (Δ*S* ^‡^) has received little attention as a strategy for controlling the rate of molecular motion. The entropic control of motion rate, which cannot be applied in the centimeter scale world, is unique to nanometer-sized scales, and we believe that such a strategy is important for expanding the design possibilities and methodologies for molecular machines.

One possible way to use activation entropy to control the rate of molecular motion is to restrict the motion or position of the molecule in a transition state that lowers the activation entropy. The water-mediated proton relay could be an effective candidate for this purpose because the well-ordered arrangement of water molecules via hydrogen bonds in the transition state of the proton relay leads to a significant decrease in the activation entropy. Inspired by artificial helical or twisted molecules with complex and asymmetric motions and functions,^16,29–32^ our group has focused on controlling the helicity inversion motion of twisted macrocycles. Therefore, if such a mechanism can be used to confine solvent molecules in the transition state, then the entropy term of activation can be used to control the rate of molecular motions such as helicity inversion (Figure 1a).

**Figure 1:**
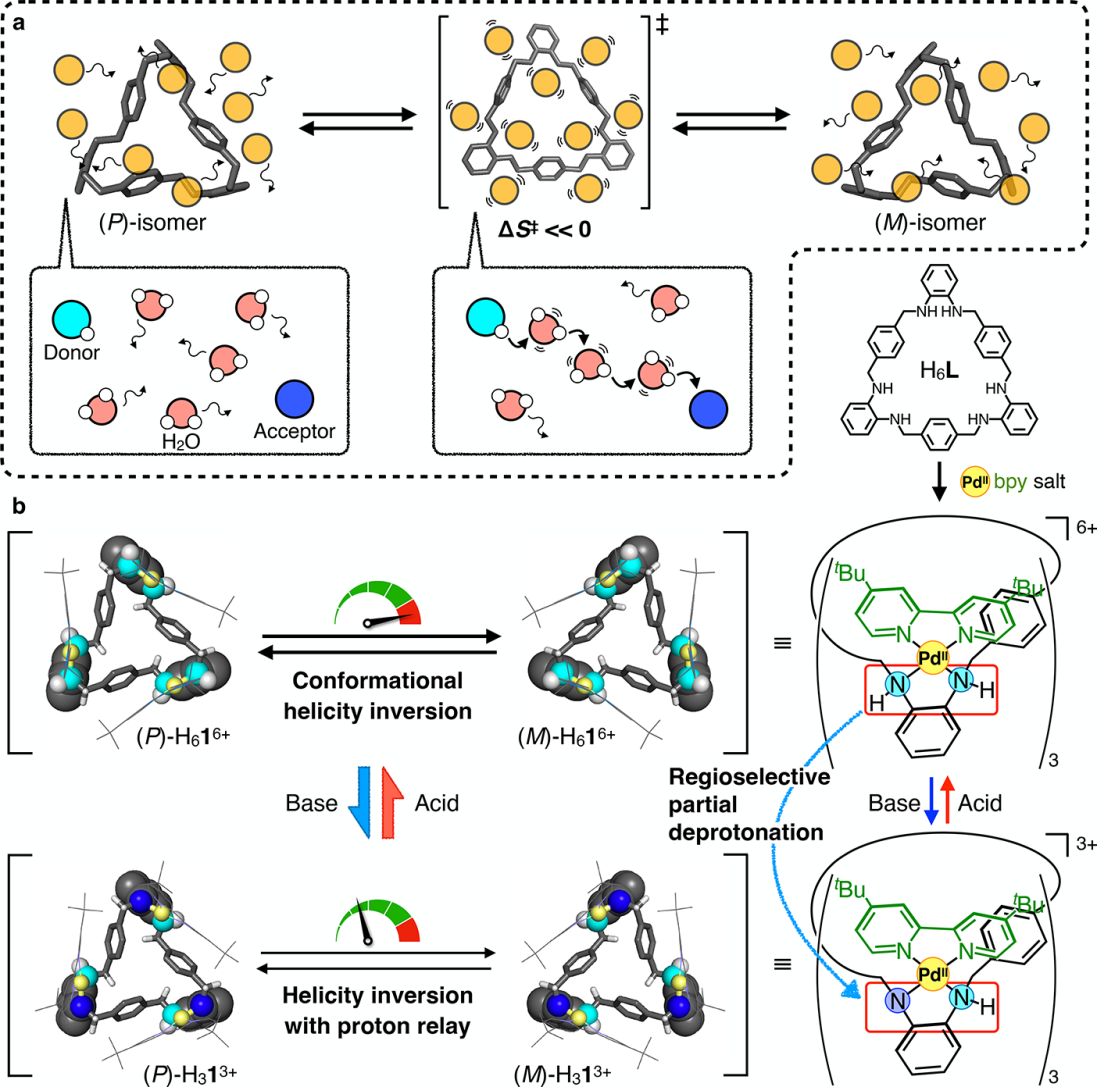
Concept of this work. (a) Conceptual figure depicting the control of the rate of molecular motion by the entropy of activation (Δ*S* ^‡^) due to the restriction of solvent molecules in the transition state, and a schematic diagram of the water-mediated proton relay with a decrease in the entropy of activation in the transition state. (b) This work: diagram of the formation of trinuclear complexes from a tris­(*ortho*-phenylenediamine) macrocycle H_6_ **L**. [Pd_3_(H_6_ **L**)­(^*t*^Bu_2_bpy)_3_]­(OTf)_6_ = H_6_ **1**·6OTf shows conformational helicity inversion between the (*P*)- and (*M*)-isomers, and [Pd_3_(H_3_ **L**)­(^*t*^Bu_2_bpy)_3_]­(OTf)_3_ = H_3_ **1**·3OTf exhibits helicity inversion between the (*P*)- and (*M*)-isomers accompanied by proton transfer.

We recently reported the selective synthesis of a trinuclear Pd^II^ complex, ([Pd_3_(H_6_ **L**)­(^*t*^Bu_2_bpy)_3_]­(OTf)_6_ = H_6_ **1**·6OTf), with a twisted tris­(*ortho*-phenylenediamine) macrocycle that exhibits a conformational helicity inversion motion (Figure 1b).^33^ This macrocyclic complex and its isomers exhibited significantly different inversion rates due to the different modes of twisting. This is attributed to the presence or absence of bond dissociation during the inversion process. Therefore, these inversion systems are enthalpically controlled by the change in the twisting mode, and entropic control of the inversion motion by the same twisting mode has not been demonstrated.

Here, we achieved the entropic control of the helicity inversion rate via reversible and regioselective partial deprotonation of the twisted trinuclear Pd^II^ hexaazamacrocycle (Figure 1b). In general, when a 1,2-diamine bidentate ligand binds to a metal, the two NH sites of the ligand are deprotonated with a weaker base, but when one of the protons is eliminated to become N^–^, it is expected that a stronger base will be required to deprotonate the other NH due to the repulsion of the negative charge in the product. Furthermore, if these NH sites are chemically unequal, then the more acidic NH will be deprotonated first. Therefore, it was expected that the use of a base with moderate basicity would enable regioselective stepwise (partial) deprotonation. Indeed, the reaction of H_6_ **1** ^6+^ with a base in acetone-*d*_6_ resulted in regioselective deprotonation of three of the six amine protons in the *ortho*-phenylenediamine moieties to form [Pd_3_(H_3_ **L**)­(^*t*^Bu_2_bpy)_3_]­(OTf)_3_ = H_3_ **1**·3OTf. H_6_ **1** ^6+^ and H_3_ **1** ^3+^ were interconvertible upon addition of acids and bases, respectively. The partially deprotonated H_3_ **1** ^3+^ exhibited helicity inversion between the (*P*)- and (*M*)-isomers accompanied by proton transfer from the N–H moiety to the N^–^ moiety of each phenylenediamine moiety, resulting in a slower helicity inversion rate. Compared with the helicity inversion rate of H_6_ **1** ^6+^ in acetone-*d*_6_ at 300 K, the inversion rate of H_3_ **1** ^3+^ was 20 times slower, and the enthalpy and entropy of activation were significantly decreased by the regioselective deprotonation. Despite the decrease in the activation enthalpy, the inversion rate became slower because the activation entropy term was reduced more significantly than the activation enthalpy term, indicating that the activation entropy term dominates in controlling the helicity inversion rate. The kinetic isotope effects on the helicity inversion rate of H_3_ **1** ^3+^ by H_2_O or D_2_O suggest that a proton relay via water molecules is involved in the inversion process. These findings indicate that the slowing of the helicity inversion rate is due to a decrease in the entropy term of activation caused by the water-mediated proton transfer between the two amine nitrogen atoms in the transition state. This strategy to control the rate of molecular motion is an excellent example of controlling the rate of molecular motion via modulation of the activation entropy term, which differs from the conventional methods using the activation enthalpy term.

## Results and Discussion

### Regioselective Partial Deprotonation of a Trinuclear Pd^II^ Complex

In a previous study, a twisted trinuclear Pd^II^ complex H_6_ **1** ^6+^ was synthesized by a two-step reaction of the macrocyclic hexaamine ligand H_6_ **L** with [Pd­(^*t*^Bu_2_bpy)­(OH_2_)_2_]­(OTf)_2_ (^*t*^Bu_2_bpy = 4,4′-di-*tert*-butyl-2,2′-bipyridine).^33^ Single-crystal X-ray diffraction (XRD) and ^1^H NMR analyses revealed that H_6_ **1** ^6+^ has a helically twisted structure with *C*_3_ symmetry, which is caused by the formation of intramolecular C–H···π interactions, in which three of the six methylene groups point inside. As a result, the two amine proton signals (H_a_ and H_b_) of each *ortho*-phenylenediamine moiety became chemically inequivalent and showed separate signals in the ^1^H NMR spectrum.

In this study, we first undertook the regioselective partial deprotonation of H_6_ **1** ^6+^ (Figure 2). When diisopropylethylamine (DIPEA; 5.0 equiv) was added to an acetone-*d*_6_ solution of H_6_ **1**·6OTf at room temperature in air, the color of the solution immediately changed from colorless to red-orange. 1D ^1^H and 2D ^1^H–^1^H COSY and ROESY NMR analyses of this solution showed the disappearance of the amine proton H_a_ next to methylene not involved in C–H···π interactions. This was also supported by the disappearance of correlations between H_a_ and the adjacent methylene protons (H_e_ and H_f_) in the COSY spectrum (Figures S3 and S7). The upfield shifts of the methylene protons (H_e_ and H_f_) were also consistent with the deprotonation of H_a_. Considering the *C*_3_ symmetry of this complex, this result indicates that three of the six amine protons were regioselectively deprotonated by DIPEA. Remarkably, the helically twisted conformation with the intramolecular C–H···π interactions was preserved after the regioselective partial deprotonation, as supported by the fact that H_c_ remained upshifted relative to the other methylene protons.

**Figure 2:**
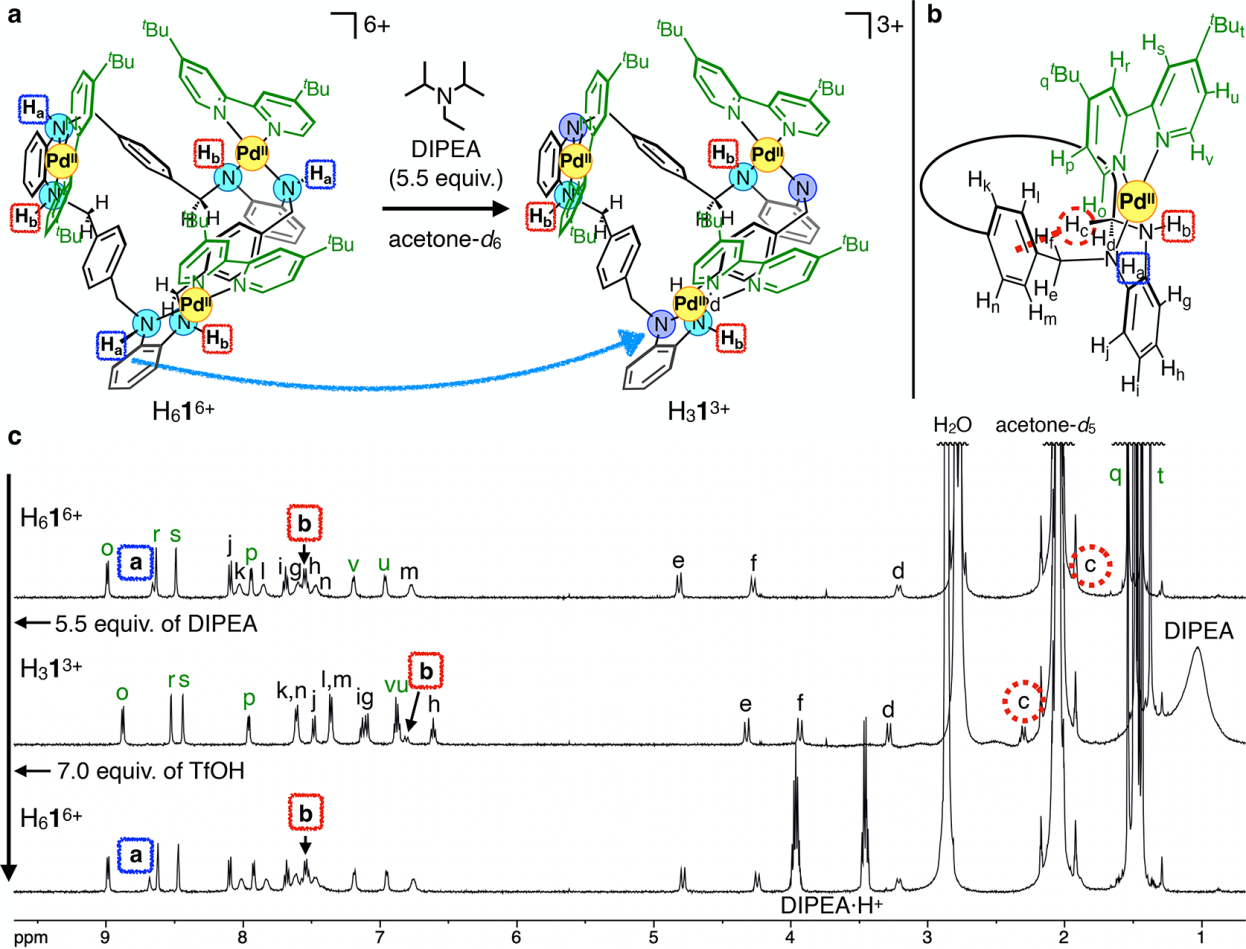
Regioselective partial deprotonation of H_6_ **1** ^6+^ to form H_3_ **1** ^3+^. (a) Schematic diagram of the deprotonation of H_6_ **1** ^6+^ with 5.5 equiv of DIPEA to form H_3_ **1** ^3+^. For clarity, one enantiomer of H_6_ **1** ^6+^ and H_3_ **1** ^3+^ is shown. (b) Partial structural formula of H_6_ **1** ^6+^. (c) ^1^H NMR spectra of H_6_ **1** ^6+^ and H_3_ **1** ^3+^ upon addition of 5.5 equiv of DIPEA and 7.0 equiv of TfOH (500 MHz, acetone-*d*_6_, 300 K). The proton signal H_c_ overlapped with that of acetone-*d*_5_, and its position was confirmed by ^1^H–^1^H COSY NMR analysis (Figures S2 and S3).

Next, we investigated the reversibility of the partial deprotonation reaction. Upon addition of trifluoromethanesulfonic acid (TfOH), the red-orange color of the solution immediately turned colorless, and the ^1^H NMR signals of H_6_ **1** ^6+^ recovered within 15 min after the addition of TfOH (Figure 2c). DIPEA and TfOH were added alternately for up to three cycles, each time recovering the NMR signals of the deprotonated and original complexes (Figure S11). The results showed a reversible interconversion between the two states. This rapid and reversible interconversion suggests that the deprotonation occurs without an oxidation reaction, as supported by the observation of the sharp signals without paramagnetic shifts in the NMR spectra. The absence of an oxidation reaction was also supported by density functional theory (DFT) calculations, which are discussed below. Therefore, the composition of the deprotonated complex was concluded to be [Pd_3_(H_3_ **L**)­(^*t*^Bu_2_bpy)_3_]­(OTf)_3_ = H_3_ **1**·3OTf.

Regioselective deprotonation of H_6_ **1** ^6+^ was similarly observed with Na_2_CO_3_ or 1,8-bis­(dimethylamino)­naphthalene (a proton sponge). For example, 26 equiv of solid Na_2_CO_3_ was added to an acetone-*d*_6_ solution of H_6_ **1** ^6+^, and the mixture was sonicated for 5 min; the color of the solution gradually changed to red-orange. The resulting insoluble inorganic salts were removed by centrifugation, leaving 3 equiv of NaOTf in the acetone solution. The ^1^H NMR spectrum of this solution was nearly identical to that of H_3_ **1** ^3+^ deprotonated by DIPEA (Figure S12). High-resolution electrospray ionization time-of-flight (ESI-TOF) mass spectrometry also supported the formation of H_3_ **1** ^3+^ (*m*/*z* = 950.2865 as [H_3_ **1**·OTf]^2+^; Figure S16). These results indicate that partially deprotonated H_3_ **1** ^3+^ was selectively formed even during the reaction with excess solid Na_2_CO_3_. In the case of proton sponge, the amine protons were quantitatively deprotonated, which was confirmed by ^1^H NMR spectroscopy (Figure S18). When 3.3 equiv of proton sponge was added, the signals of reacted and unreacted proton sponge were observed separately in the ^1^H NMR spectrum, and the molar ratio of H_3_ **1** ^3+^ to protonated proton sponge calculated based on the integral values was nearly 1:3. This result also confirmed that only three of the six amine protons in H_6_ **1** ^6+^ were deprotonated.

The red-orange acetone solution of H_3_ **1** ^3+^ was analyzed by UV–vis spectroscopy. As a result, a new absorption band was observed at approximately 465 nm at 293 K in the UV–vis spectrum of an acetone solution of H_3_ **1** ^3+^ deprotonated by Na_2_CO_3_. In contrast, a colorless acetone solution of H_6_ **1** ^6+^ showed no absorption in the visible region (Figure 3a–c and Figure S17). Nearly the same absorption was observed for samples deprotonated with DIPEA, and the increase in absorption nearly saturated with 3.5 equiv of DIPEA in acetone at 293 K ([H_6_ **1**·6OTf] = 82 μM) (Figure S10). Furthermore, the addition of 100 equiv of DIPEA did not change the UV–vis spectrum of H_3_ **1** ^3+^, suggesting that no further deprotonation occurred under these conditions. On the other hand, when ^*t*^BuOK was used in dry tetrahydrofuran (THF) as a stronger base instead of DIPEA, further deprotonation was suggested by changes in the UV–vis spectrum (see the Sections S2.8 and S5.6 for detail).

**Figure 3:**
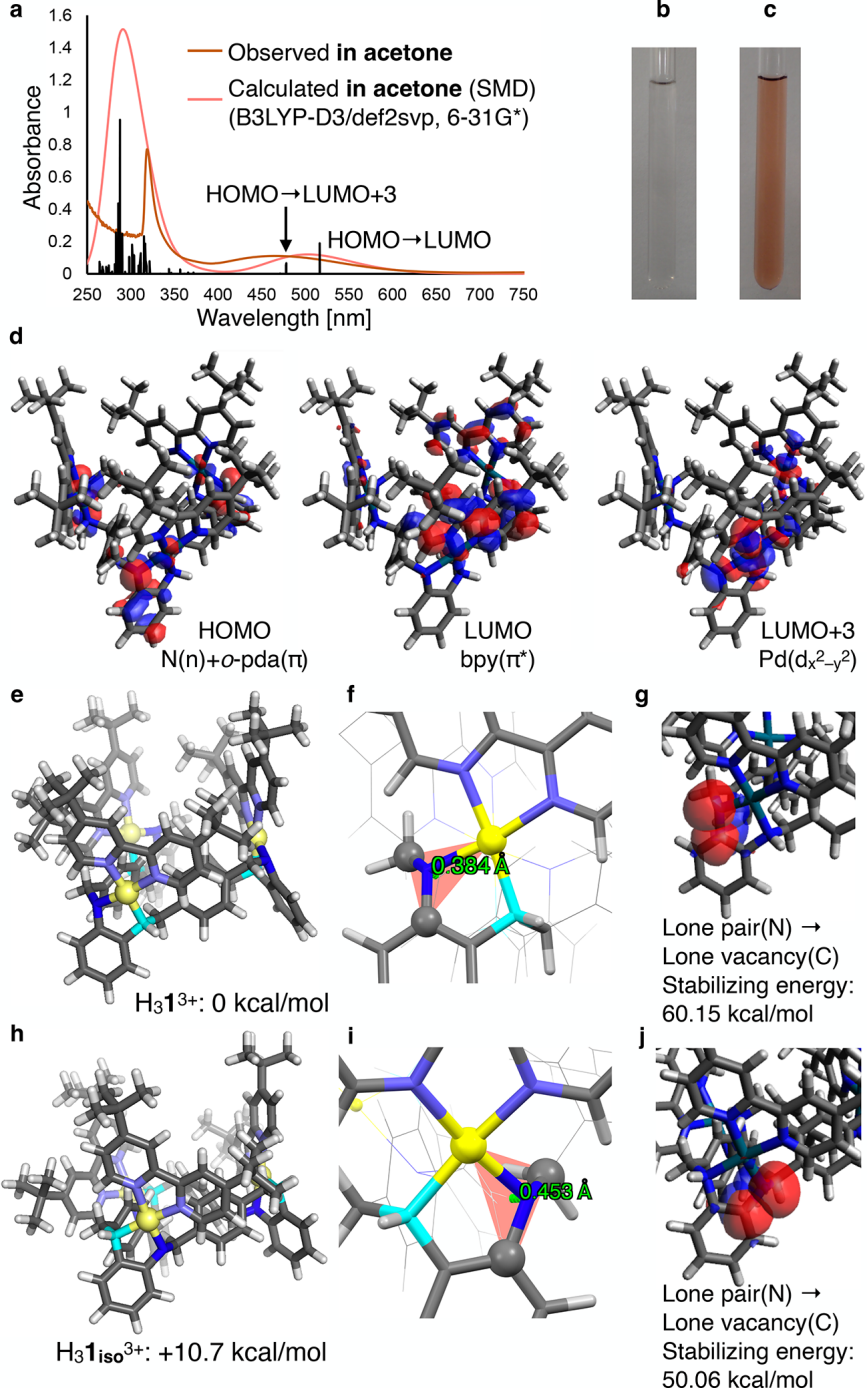
DFT calculations of H_3_ **1** ^3+^. (a) Observed UV–vis spectrum of H_3_ **1** ^3+^ deprotonated by Na_2_CO_3_ (acetone; 293 K, *l* = 0.2 cm; 96 μM) (brown) and calculated absorption spectra of H_3_ **1** ^3+^ in acetone (pink and black). (b, c) Photographs of H_6_ **1** ^6+^ and H_3_ **1** ^3+^ in acetone-*d*_6_. (d) HOMO, LUMO, and LUMO+3 of H_3_ **1** ^3+^ in acetone. (e, h) Optimized structures of H_3_ **1** ^3+^ and H_3_ **1_iso_** ^3+^ and their relative Gibbs free energies to H_3_ **1** ^3+^. (f, i) Enlarged images of H_3_ **1** ^3+^ and H_3_ **1_iso_** ^3+^ optimized for the heights of the deprotonated nitrogen atom above the Pd–C–C planes. (g, j) Superposition of donor (lone pair (N)) and acceptor (lone vacancy (C)) NBOs (isovalue = 0.03) for H_3_ **1** ^3+^ and H_3_ **1_iso_** ^3+^ with stabilization energies of 60.2 and 50.1 kcal/mol, respectively.

### DFT Calculations of H_3_1^3+^

DFT and time-dependent DFT (TD-DFT) calculations were performed to analyze the characteristic absorption of H_3_ **1** ^3+^, and the electronic states of H_3_ **1** ^3+^ were discussed.^34–38^ Computational details are referred to Section S5. The geometry optimization provided the helically twisted backbone structure of H_3_ **1** ^3+^, which is consistent with the results of ^1^H NMR analyses. TD-DFT calculations were performed considering the solvent effects in acetone using the solvation model density (SMD) method.^37^ The calculated spectrum accurately reproduced the experimental spectrum, and the absorption at approximately 465 nm was assigned to the interligand charge transfer (ILCT) from the lone pair orbitals (HOMO–HOMO–2) of the deprotonated amine nitrogen moieties to the π* orbitals (LUMO–LUMO+2) of the bipyridine moieties and ligand-to-metal charge transfer (LMCT) from the lone pair orbitals (HOMO–HOMO–2) of the deprotonated amine moieties to the d_*x* ^2^__–*y* ^2^_ orbitals (LUMO+3–LUMO+5) of the Pd^II^ centers (Figure 3a,d). We also confirmed these assignments with other functionals including long-range corrections (Section S5.3). Compared with the optimized trinuclear Pd^II^ complex before deprotonation, the HOMO energy level was significantly destabilized by deprotonation (Figure S92). These results indicate that the red-orange color of H_3_ **1** ^3+^ is due to ILCT and LMCT and not due to oxidation under the reaction conditions.

Next, we used DFT calculations to explain why the amine protons, H_a_, are regioselectively deprotonated rather than H_b_. The isomeric trinuclear Pd^II^ complex with the other three amine protons (H_b_) deprotonated (H_3_ **1**_**iso**_^3+^) was also optimized. Comparison of the Gibbs free energies of H_3_ **1** ^3+^ and H_3_ **1**_**iso**_^3+^ in acetone revealed that H_3_ **1** ^3+^ was 10.7 kcal/mol more stable than H_3_ **1**_**iso**_^3+^ (Figure 3e,h). This is consistent with the regioselective deprotonation of H_a_ observed in the ^1^H NMR spectrum. This energy difference can be explained by the stability of the lone pairs on the deprotonated amine–nitrogen atoms in the optimized structures (Figure 3f,i). That is, the amine nitrogen atoms with H_a_ deprotonated have a more planar geometry, and the lone pairs can be more effectively delocalized to the adjacent phenyl rings. This makes H_3_ **1** ^3+^ more stable, as supported by natural bond orbital (NBO) analysis,^38^ which shows that the stabilization by the interaction between the lone-pair orbitals and the adjacent vacant π* orbitals is larger for H_3_ **1** ^3+^ than for H_3_ **1**_**iso**_^3+^ (Figure 3g,j).

### Comparison with Model Pd^II^ Complexes

Crystals of H_3_ **1** ^3+^ could not be obtained despite numerous crystallization trials, so we synthesized and crystallized a model Pd^II^ complex, [Pd­(H_–1_ *o*-pda)­(^*t*^Bu_2_bpy)]­(OTf) = H**2**·OTf (*o*-pda = *ortho*-phenylenediamine), a partial structure of H_3_ **1** ^3+^, to clarify the structure of the partially deprotonated *ortho*-phenylenediamine moiety. When DIPEA was added to a THF solution of the colorless Pd^II^ model complex [Pd­(*o*-pda)­(^*t*^Bu_2_bpy)]­(OTf)_2_ = H_2_ **2**·2OTf, the color of the solution rapidly changed from colorless to orange. When the reaction solution was allowed to stand at room temperature overnight, red-orange needle-like crystals were obtained. The resulting red-orange crystals were found to consist of the desired partially deprotonated model complex with the triflate anion H**2**·OTf as revealed by single-crystal XRD analysis (Figure 4). In the crystal structure of H**2** ^+^, the bond lengths of Pd–N1 and C1–N1 are 0.060 and 0.063 Å shorter than those of H_2_ **2** ^2+^ (2.037 and 1.456 Å), respectively, suggesting monodeprotonation from N1. These shorter bond lengths are consistent with the calculated structure of H_3_ **1** ^3+^ (Figure S75). In particular, the short C1–N1 length is explained by the delocalization of the lone pair of electrons of N1 into the lone vacancy orbitals of C1, as discussed in the previous DFT calculation section. The monodeprotonation was also confirmed by X-ray photoelectron spectroscopy and elemental analysis (Figures S48–S50).

**Figure 4:**
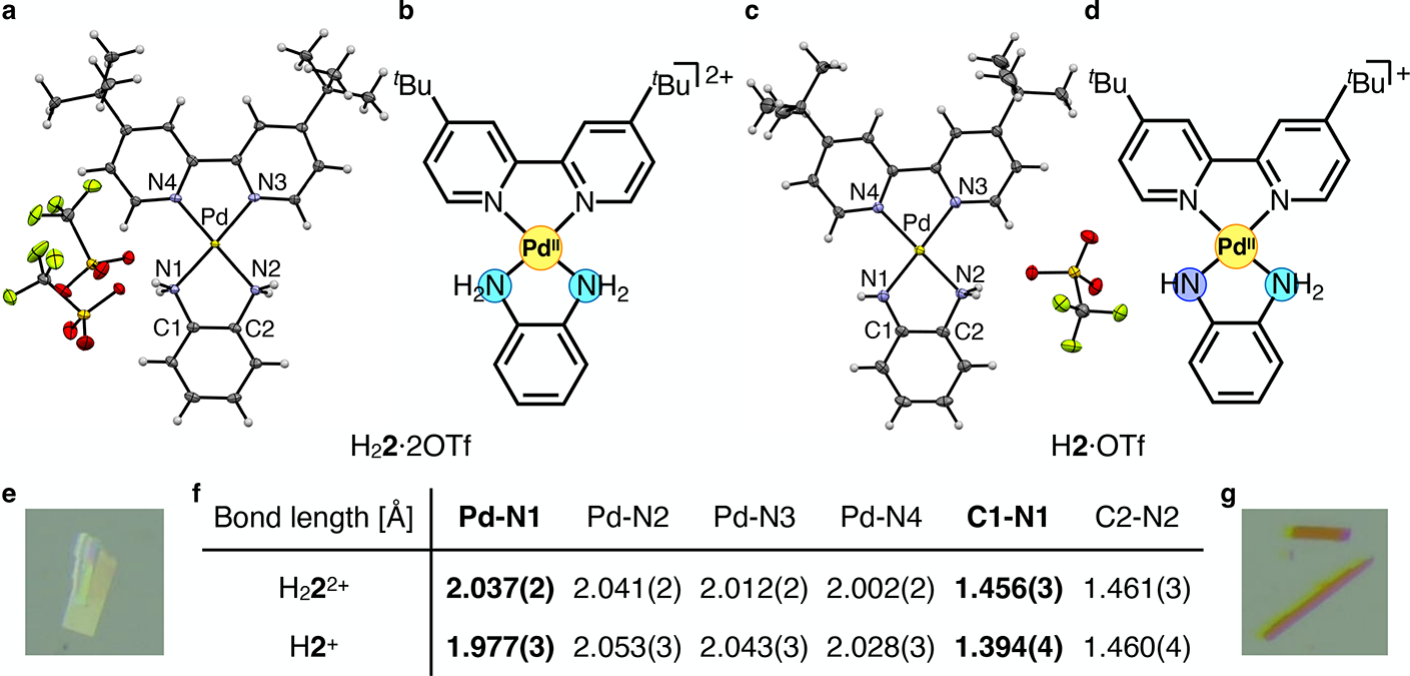
Structures of model Pd^II^ complexes. (a, c) ORTEP drawings of H_2_ **2**·2OTf and H**2**·OTf at the 50% probability level. (b, d) Chemical formula of H_2_ **2** ^2+^ and H**2** ^+^. (e, g) Photographs of the crystals of H_2_ **2**·2OTf and H**2**·OTf. (f) Bond lengths of H_2_ **2** ^2+^ and H**2** ^+^ in the crystal structures.

Next, we investigated the deprotonation behavior of the model complex in solution. When H_2_ **2** ^2+^ was reacted with 2.6 equiv of proton sponge in acetone-*d*_6_, the molar ratio of the resulting Pd^II^ model complex to the protonated proton sponge was estimated to be nearly 1:1 by the ^1^H NMR spectrum, suggesting that only one amine proton of H_2_ **2** ^2+^ was deprotonated (Figure S43). Furthermore, the UV–vis spectrum of an acetone solution of H**2** ^+^ exhibited an absorption band at approximately 500 nm in the visible region (Figure S45). These partial deprotonation behaviors without oxidation were consistent with the regioselective partial deprotonation of H_6_ **1** ^6+^. The reactivity is quite different from that of the reported bis­(*o*-pda)platinum complexes, which showed the deprotonation of two protons from a single *ortho*-phenylenediamine moiety and the subsequent oxidation in aqueous ammonia,^39,40^ mainly due to the difference in the solvent systems used.

### Helicity Inversion of H_3_1^3+^ Accompanied by Proton Transfer

We previously reported that H_6_ **1** ^6+^ exhibits helicity inversion between the (*P*)- and (*M*)-isomers.^33^ Furthermore, the helicity inversion rate, enthalpy (Δ*H* ^‡^), entropy (Δ*S* ^‡^), and Gibbs free energy (Δ*G* ^‡^) of activation of H_6_ **1** ^6+^ were estimated to be 3.31 s^–1^, 86.1 kJ/mol, 51.9 J/(mol·K), and 70.6 kJ/mol, respectively, by exchange spectroscopy (EXSY)^41^ and Eyring plots. Notably, H_6_ **1** ^6+^ exhibited conformational helicity inversion and did not require bond dissociation.

To evaluate the effect of regioselective partial deprotonation on the rate of helicity inversion between the (*P*)- and (*M*)-isomers, EXSY was performed on H_3_ **1** ^3+^ deprotonated with Na_2_CO_3_. Chemical exchange signals between H_d_ and H_f_ were observed in the EXSY spectra, and the helicity inversion rate of H_3_ **1** ^3+^ in acetone-*d*_6_ at 300 K was estimated to be 0.167 s^–1^, which is 20 times slower than that of H_6_ **1** ^6+^ (Figure 5). On the other hand, in an equimolar mixture of (*P*)- and (*M*)-isomers, no circular dichroism signal is observed because no bias in the molar ratio occurs due to bidirectional helicity inversion. Activation parameters were also estimated by EXSY at various temperatures and Eyring plots (Δ*H* ^‡^ = 42 kJ/mol, Δ*S* ^‡^ = −119 J/(mol·K), and Δ*G* ^‡^ = 78 kJ/mol at 300 K). Comparing the activation parameters of H_6_ **1** ^6+^, the Gibbs free energy of activation increased despite the decrease in the activation enthalpy of H_3_ **1** ^3+^ (ΔΔ*H* ^‡^ = −44 kJ/mol), indicating that the large decrease in the activation entropy term (Δ­(*T*Δ*S* ^‡^) = −51.3 kJ/mol at 300 K) mainly affected the slowdown of the helicity inversion of H_3_ **1** ^3+^. The large differences in the activation enthalpy and entropy between H_6_ **1** ^6+^ and H_3_ **1** ^3+^ also suggest that the helicity inversion mechanism of H_3_ **1** ^3+^ is different from the conformational inversion of H_6_ **1** ^6+^.

**Figure 5:**
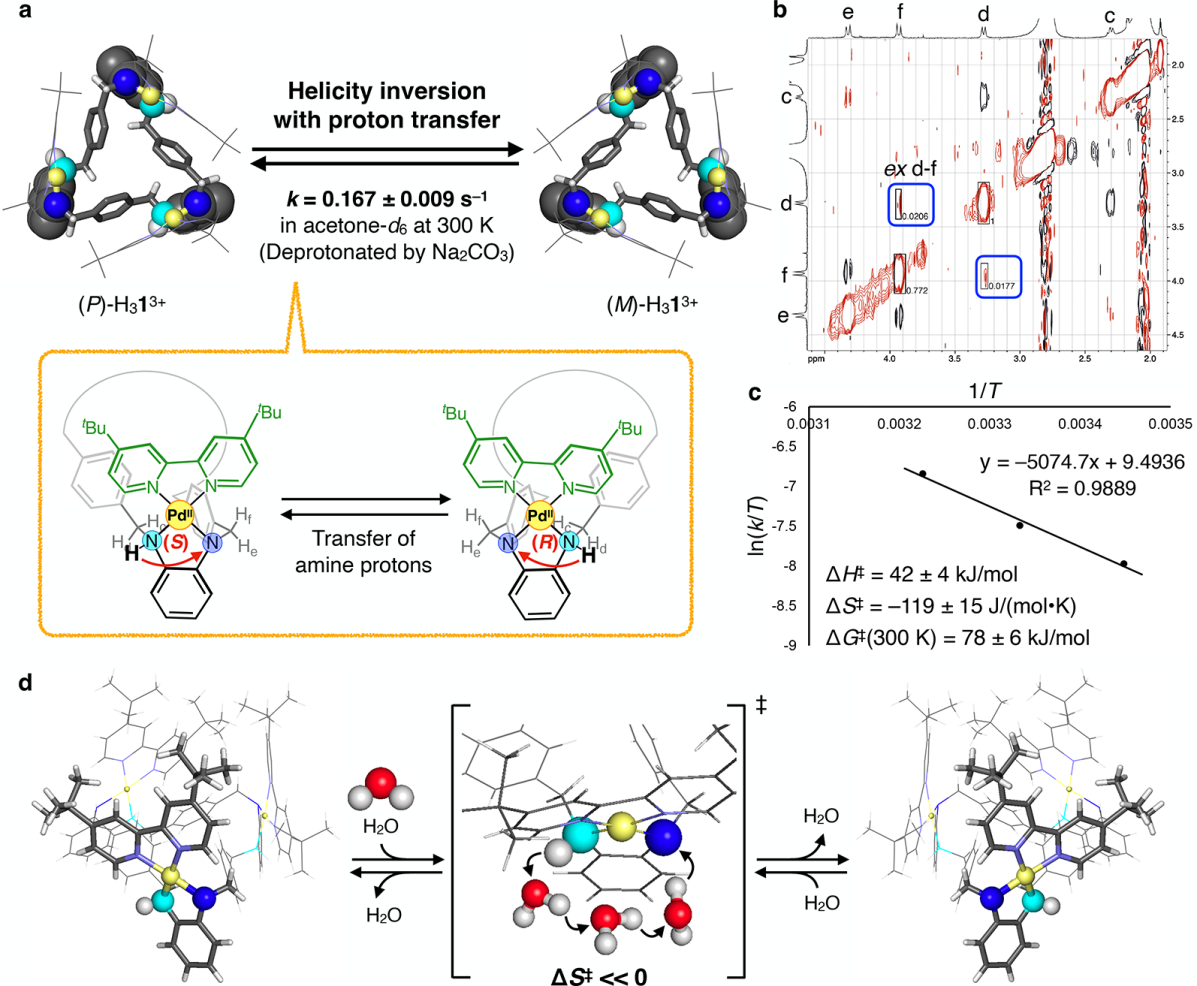
Estimation of the helicity inversion rate of H_3_ **1** ^3+^. (a) Schematic diagram of the helicity inversion between (*P*)- and (*M*)-H_3_ **1** ^3+^. The inversion is accompanied by amine proton transfer to the adjacent amine site. (b) EXSY spectrum of (*P*/*M*)-H_3_ **1** ^3+^ (500 MHz acetone-*d*_6_, 300 K; 0.27 mM). The inversion rate was estimated using the chemical exchange signals between H_d_ and H_f_ (*ex* d-f), shown in blue. (c) Eyring plot for the helicity inversion of (*P*/*M*)-H_3_ **1** ^3+^. (d) Proposed mechanism for the helicity inversion of (*P*/*M*)-H_3_ **1** ^3+^: the amine proton transfers via water molecules, decreasing the activation entropy.

To analyze the mechanism of helicity inversion of H_3_ **1** ^3+^, we first focused on the structural differences between the (*P*)- and (*M*)-isomers. The structural comparison of (*P*)- and (*M*)-H_3_ **1** ^3+^ demonstrated that the helicity inversion of H_3_ **1** ^3+^ requires the transfer of amine protons. For example, when (*P*)-H_3_ **1** ^3+^ inverts to the (*M*)-isomer, the amine protons attached to the nitrogen atoms in the (*S*)-configuration transfers to the other amine nitrogen atoms (Figure 5a). Furthermore, the DFT-optimized structure showed that the two amine nitrogen atoms of each *ortho*-phenylenediamine moiety were too far apart for the amine protons to be transferred directly to the nearest amine nitrogen atom. Therefore, it was thought that the proton transfer was mediated by the solvent, water molecules contained in the solvent and the counteranions.

To gain further insight into the proton transfer process, we investigated the effect of the amount of water on the helicity inversion of H_3_ **1** ^3+^. First, the inversion rate in distilled acetone-*d*_6_, which contains approximately one-third the amount of water compared to undried acetone-*d*_6_ at room temperature, was estimated to be 0.0593 s^–1^ at 300 K. This rate was three times slower than that in undried acetone-*d*_6_ (0.167 s^–1^). In contrast, the inversion rate was accelerated by a factor of 4 (*k*_H_ = 0.704 s^–1^ at 300 K) in an acetone-*d*_6_–H_2_O solution (100:1 (v/v)) prepared by adding H_2_O to the undried acetone-*d*_6_ solution. These results suggest that proton transfer is accelerated by the mediation of water molecules during helicity inversion. Next, to investigate the effect of the kinetic isotope on the helicity inversion rate, D_2_O instead of H_2_O was added to an acetone-*d*_6_ solution of H_3_ **1** ^3+^. Under these conditions, most of protons of the amine moieties and water molecules were replaced by deuterons. The inversion rate (*k*_D_) in acetone-*d*_6_:D_2_O = 100:1 (v/v) was estimated to be 0.51 s^–1^, and the observed kinetic isotope effect (*k*_H_/*k*_D_ = 1.4) suggested that proton transfer was involved in the rate-determining step. Notably, this *k*_H_/*k*_D_ value is consistent with the reported kinetic isotope effect of proton hopping in the Grotthuss mechanism.^42^ Thus, this mechanism appears to involve multiple water molecules relaying proton transport through hydrogen bond recombination, rather than other mediated mechanisms involving single-molecule transport processes (vehicle mechanisms). The kinetic isotope effects for the inversion of H_3_ **1** ^3+^ in acetone-*d*_6_:CH_3_OH/CD_3_OD = 100:1 (v/v) was also consistent with this mechanism (Section S4.6). In contrast, no kinetic isotope effect (*k*_H_/*k*_D_ = 1.02) was observed in control experiments with nondeprotonated H_6_ **1** ^6+^ in acetone-*d*_6_:H_2_O or D_2_O = 100:1 (v/v). These results and the large negative value of the activation entropy are consistent with a mechanism in which water-mediated proton relay between amine nitrogen atoms plays a role in the rate-determining step of the helicity inversion of H_3_ **1** ^3+^. This mechanism requires several water molecules to line up in a well-ordered manner to transfer the protons in the transition state, resulting in a significant reduction in the activation entropy (Figure 5d) and indicating the entropic control of the helicity inversion rates.

Finally, we investigated the effects of other protic substrates on the helicity inversion rate of H_3_ **1** ^3+^. Estimation of the inversion rates in acetone-*d*_6_–CH_3_OH (100:1 (v/v)) as in water showed that the inversion was similarly accelerated to 0.55 s^–1^, suggesting that the proton transfer may also be mediated by the hydroxy groups of the protic methanol molecules. In contrast, the addition of aprotic CH_3_CN instead of CH_3_OH did not accelerate the inversion rate (0.082 s^–1^ in acetone-*d*_6_:CH_3_CN = 100:1 (v/v)). These results suggest that the protic nature of the solvent, rather than its polarity, played a key role in accelerating the inversion rates. This is supported by a similar experiment conducted using proton sponge (4.2 equiv) as a base instead of Na_2_CO_3_. The inversion rate was estimated to be 0.57 s^–1^ in acetone-*d*_6_ at 300 K, which is faster than that for Na_2_CO_3_, suggesting that the conjugate acid of this reaction, i.e., the protonated proton sponge, also acted as a protic mediator to accelerate the proton relay.

## Conclusions

In this study, we successfully synthesized a partially deprotonated trinuclear Pd^II^ macrocycle that exhibited helicity inversion using a proton relay. In the reaction of a moderate base with Pd^II^ macrocycle H_6_ **1** ^6+^, three of the six amine protons regioselectively deprotonated to form H_3_ **1** ^3+^ utilizing the difference in the acidity of two chemically inequivalent NH sites of each *ortho*-phenylenediamine, as suggested by DFT calculations. The helicity inversion rate of H_3_ **1** ^3+^ was estimated to be 0.167 s^–1^, which was 20 times slower than that of H_6_ **1** ^6+^. The slowdown in the inversion rate due to deprotonation was ascribed to a large decrease in the entropy term of activation, which exceeded the decrease in the enthalpy term of activation. The kinetic isotope effects on the helicity inversion suggest that a proton relay through aligned water molecules was included in the transition state of the inversion process to decrease the entropy of activation. Our strategy for controlling the helicity inversion rate using the entropy term of activation differs from typical enthalpic control of the rate of molecular motion. Incorporating the proton relay into a molecular machine to entropically control the rate of molecular motion is expected to expand its designability for the construction of more sophisticated molecular machines.

## Supplementary Material



## Notes

Additional experimental details, materials, and methods, including NMR, UV–vis, ESI-MS, XRD, and XPS analyses, and DFT calculations; crystallographic data (PDF)
