# Supplementary material for: Entropic Control of the Helicity Inversion Rates of Twisted Metallomacrocycles by Reversible and Regioselective Deprotonation
Source: J Am Chem Soc. 2026 Jan 28;148(5):5338–46. doi: 10.1021/jacs.5c18601 (PMC12903852; doi:10.1021/jacs.5c18601)
Supplement: Supplementary file 1 [file ja5c18601_si_001.pdf]

Supplementary Information

Entropic control of the helicity inversion rates of twisted metallomacrocycles by reversible and regioselective deprotonation

Tomoki Nakajima[1], Shohei Tashiro[1], Masahiro Ehara[2], and Mitsuhiko Shionoya[1,3]

[1]Department of Chemistry, Graduate School of Science, The University of Tokyo, 7-3-1 Hongo, Bunkyo-ku, Tokyo 113-0033, Japan.

[2]Research Centre for Computational Science, Institute for Molecular Science, Myodaiji, Okazaki, Aichi 444-8585, Japan.

[3]Research Institute for Science and Technology, Tokyo University of Science, 2641 Yamazaki, Noda, Chiba 278-8510, Japan.

1. Materials and methods ····**3**

2. Synthesis of $H_3\mathbf{1}^{3+}$ ····**4**

2.1 Synthesis of $H_6\mathbf{1}\cdot 6OTf$ ····4

2.2 Preparation of $H_3\mathbf{1}^{3+}$ using DIPEA ····5

2.3 Titration of DIPEA to $H_6\mathbf{1}\cdot 6OTf$ in acetone and estimation of the relative acid dissociation constant of $H_6\mathbf{1}^{6+}$ ····9

2.4 Reversible transformation between $H_6\mathbf{1}^{6+}$ and $H_3\mathbf{1}^{3+}$ upon alternate addition of base and acid ····10

2.5 Preparation of $H_3\mathbf{1}^{3+}$ using Na_2CO_3 ····11

2.6 Preparation of $H_3\mathbf{1}^{3+}$ using proton sponge ····15

2.7 Titration of proton sponge to $H_6\mathbf{1}\cdot 6OTf$ in acetone-d_6 ····15

2.8 Deprotonation of $H_6\mathbf{1}^{6+}$ using ^{t}BuOK as a stronger base ····17

3. Synthesis of model Pd complexes ····**18**

3.1 Synthesis of $H_2\mathbf{2}\cdot 2OTf$ ····18

3.2 Synthesis of $H\mathbf{2}\cdot OTf$ ····24

3.3 Titration of DIPEA to H_2**2**·2OTf in acetone and estimation of the relative acid dissociation constant of H_2**2**$^{2+}$ ····33

4. Estimation of the rate of helicity inversion by EXSY NMR ····34

4.1 Theoretical analysis of the rate of helicity inversion based on ^{1}H-^{1}H EXSY NMR measurements ····34

4.2 Theoretical analysis of activation parameters of helicity inversion based on VT EXSY NMR measurements ····35

4.3 VT EXSY NMR measurements of H_3**1**$^{3+}$ in acetone-d_6 and estimation of the rate and activation parameters of the helicity inversion ····35

4.4 Estimation of the helicity inversion rate of H_3**1**$^{3+}$ in distilled acetone-d_6 ····38

4.5 Kinetic isotope effect on the helicity inversion of H_3**1**$^{3+}$ with water ····40

4.6 Kinetic isotope effect on the helicity inversion of H_3**1**$^{3+}$ with methanol ····43

4.7 Kinetic isotope effect on the helicity inversion of H_6**1**$^{6+}$ ····45

4.8 The effect of the addition of CH_3CN on the helicity inversion rate of H_3**1**$^{3+}$ ····48

4.9 Estimation of the helicity inversion rate of H_3**1**$^{3+}$ deprotonated by proton sponge ····49

5. Computational study ····52

5.1 General calculation methods and conditions ····52

5.2 DFT calculations of H_3**1**$^{3+}$ ····53

5.3 TD-DFT calculations using other basis sets and functionals ····55

5.4 Comparison between H_3**1**$^{3+}$ and H_3**1**$_{\mathbf{iso}}^{3+}$ ····58

5.5 DFT calculations of H_6**1**·OTf^{5+} ····59

5.6 DFT calculations of **1** ····63

6. References ····66

1. Materials and methods

A metal source,[1] $[Pd(^tBu_2bpy)(OH_2)_2](OTf)_2 \cdot (H_2O)_2$ (OTf = trifluoromethanesulfonate), a macrocyclic ligand H_6**L**[2] and a trinuclear Pd^{II} complex,[1] H_6**1**·6OTf, were prepared according to reported procedures, and their analytical data were referred to the reported ones. Specific references for them are noted where applicable. Other solvents, organic and inorganic reagents are commercially available, and were used without further purification.

NMR spectroscopic measurements were performed using a Bruker AVANCE 500 spectrometer (500 MHz for ^1H; 126 MHz for ^{13}C). Display of NMR spectra was performed using iNMR software. The chemical shifts were reported in parts per million (ppm) on the δ scale and were referenced to acetone-d_5 (δ= 2.05 ppm for ^1H NMR), and acetone-d_6 (δ= 29.84 ppm for ^{13}C NMR). The multiplicity of each signal for ^1H NMR was indicated by s (singlet), d (doublet), dd (double-doublet), t (triplet), m (multiplet), and brs (broad-singlet). The assignment of ^1H and ^{13}C NMR signals was supported by 2D NMR spectroscopy. ESI-TOF mass spectra were recorded on a Micromass LCT spectrometer, in which high-resolution mass (HRMS) data were collected using a leucine enkephalin as an internal standard. UV-Vis spectroscopy was performed using a JASCO V-770 spectrophotometer. IR spectra were recorded on a JASCO FT/IR-4200 spectrometer using a ZnSe ATR method. Melting point was measured by YANACO MP-500D apparatus. Single-crystal X-ray diffraction (XRD) analyses were performed using a Rigaku XtaLAB P200 diffractometer under CuKα radiation with a CrysAlisPro software package, and the obtained data were analyzed using CrystalStructure and Olex2 crystallographic software packages[3] except for refinement, which was performed using SHELXL-2013 program suite.[4] X-ray structures were displayed using Mercury software packages. Powder X-ray diffraction (pXRD) measurements were conducted using a Rigaku SmartLab diffractometer with CuKα radiation. Elemental analyses were performed in Microanalytical Laboratory, Department of Chemistry, School of Science, the University of Tokyo using Vario MICRO Cube elemental analyzer with the addition of MgO, and the creation of calibration curves and calculation of analytical values were carried out in Excel. X-ray photoelectron spectroscopy (XPS) measurement was carried out using UlvacPhi PHI5000 VersaProbe III with AlKα radiation (1486.6 eV). The helispherical electron energy analyzer operated at pass energies of 112 and 27.0 eV for survey and narrow scans, respectively. All XPS measurements were conducted using a neutralizer.

2. Synthesis of $H_3 1^{3+}$

2.1 Synthesis of $H_6 1 \cdot 6OTf$

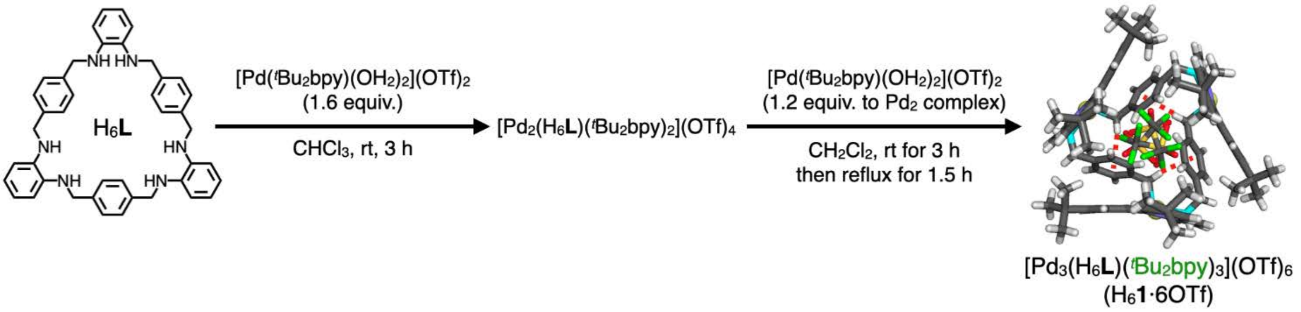

Figure S1. The synthetic scheme of a trinuclear Pd^{II} complex, $[Pd_3(H_6\mathbf{L})(^t\text{Bu}_2\text{bpy})_3](OTf)_6$ = $H_6\mathbf{1}\cdot 6OTf$. The red dotted lines in $H_6\mathbf{1}\cdot 6OTf$ indicate the intramolecular C-H···π interactions.

A trinuclear Pd^{II} complex, $[Pd_3(H_6\mathbf{L})(^t\text{Bu}_2\text{bpy})_3](OTf)_6$ = $H_6\mathbf{1}\cdot 6OTf$, was synthesized according to the reported procedure.[1]

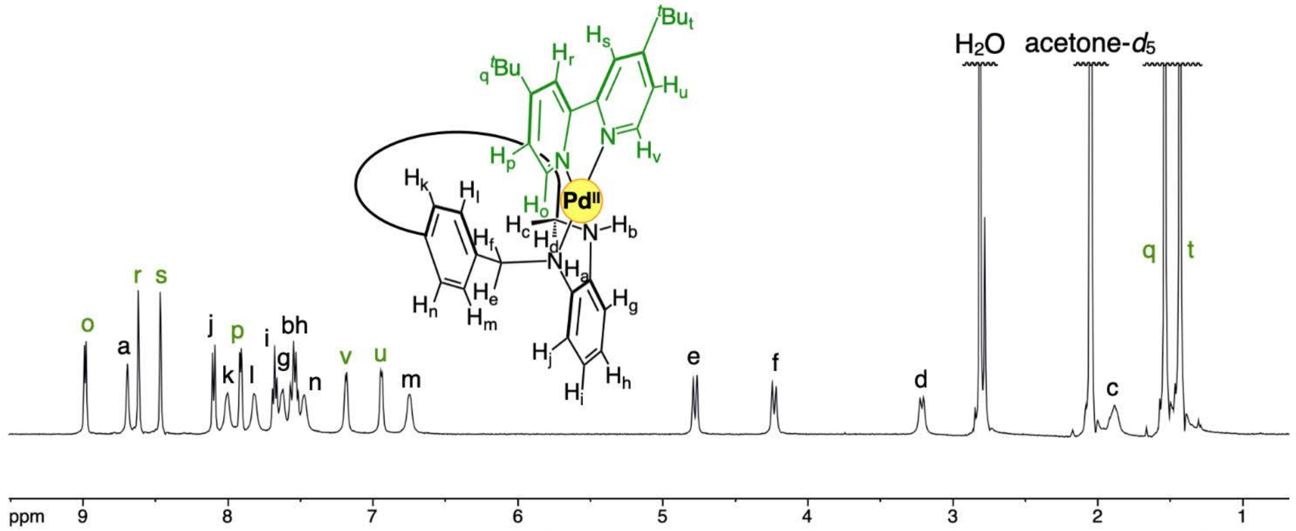

Figure S2. ^{1}H NMR spectrum of $H_6\mathbf{1}^{6+}$ (500 MHz, acetone-d_6, 300 K).

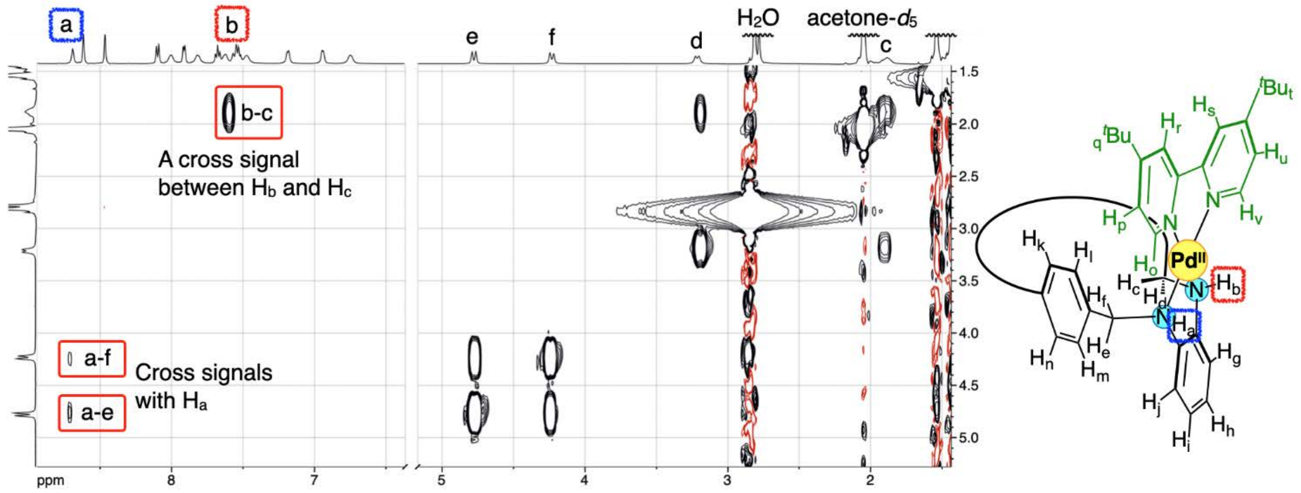

Figure S3. Enlarged view of the ^{1}H-^{1}H COSY NMR spectrum of $H_6\mathbf{1}^{6+}$ (500 MHz, acetone-d_6, 300 K) and the chemical structure of the asymmetric unit.

2.2 Preparation of $H_3\mathbf{1}^{3+}$ using DIPEA

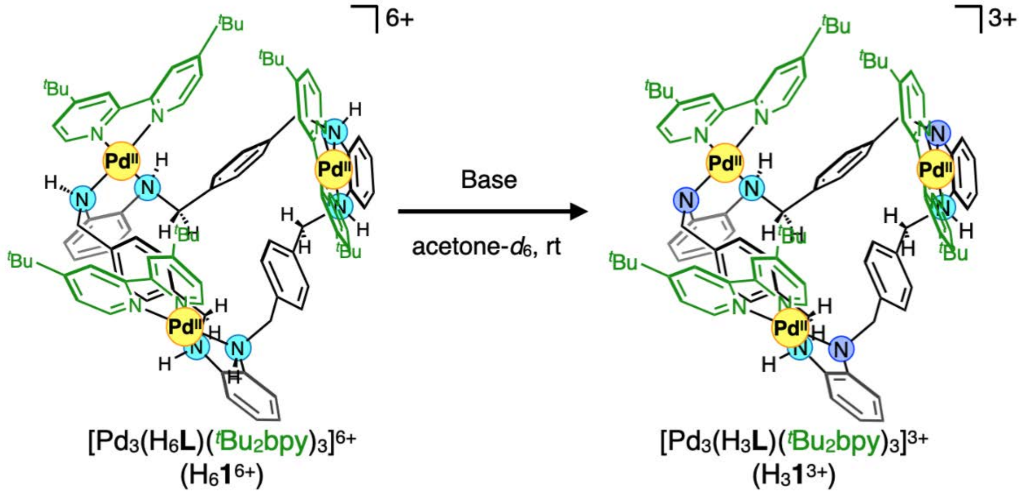

Figure S4. Scheme of the preparation of $[Pd_3(H_3\mathbf{L})(^{t}Bu_2bpy)_3](OTf)_3$ = $H_3\mathbf{1}\cdot 3OTf$.

To an acetone-d_6 solution (0.5 mL) of $H_6\mathbf{1}\cdot 6OTf$ (0.63 mg, 0.23 μmol, 1.0 equiv.) was added an acetone-d_6 solution of diisopropylethylamine (DIPEA, 5.0 μL, 230 mM, 5.0 equiv.). The color of the solution was changed from colorless to red-orange. Then, 1D ^{1}H and 2D ^{1}H-^{1}H COSY and ROESY NMR spectroscopies were conducted and the formation of the desired $H_3\mathbf{1}^{3+}$ was confirmed. This deprotonated Pd_3 complex, $H_3\mathbf{1}\cdot 3OTf$, was not so stable in the solid state and was not isolated.

Typical *ortho*-phenylenediamine metal complexes undergo one-electron and two-electron oxidation. The one-electron oxidized complexes is a radical species and exhibits no diamagnetic NMR signals. The two-electron oxidized complexes has two C=N double bonds at the phenylenediamine

moieties and no amine protons. On the other hand, the ^{1}H NMR spectra of our trinuclear complexes were completely different from those of the typical oxidized phenylenediamine complexes. Therefore we concluded that $H_3\mathbf{1}^{6+}$ was deprotonated to $H_3\mathbf{1}^{3+}$ without oxidation. The XPS spectrum of model complex H**2**·OTf was also consistent with these results (Figures S48, S49).

^{1}H NMR (500 MHz, acetone-d_6, 300 K): δ = 8.87 (d, J = 6.0 Hz, 3H), 8.52 (d, J = 1.0 Hz, 3H), 8.44, (s, 3H), 7.95 (dd, J = 5.5, 1.5 Hz, 3H), 7.60 (d, J = 7.0 Hz, 6H), 7.48 (d, J = 8.0 Hz, 3H), 7.35 (d, J = 7.5 Hz, 6H), 7.12 (t, J = 7.5 Hz, 3H), 7.10 (d, J = 7.0 Hz, 3H), 6.90 (d, J = 6.0 Hz, 3H), 6.86 (d, J = 5.5 Hz, 3H), 6.84 (brs, 3H), 6.61 (t, J = 7.0 Hz, 3H), 4.32 (d, J = 14.0 Hz, 3H), 3.93 (d, J = 14.0 Hz, 3H), 3.27 (d, J = 12.0 Hz, 3H), 2.30 (d, J = 11.5 Hz, 3H), 1.49 (s, 27H), 1.37 (s, 27H).

UV-vis (acetone, 293 K, 75.7 μM): λ_{max} (nm) (ε (M^{-1} cm^{-1})) = 475.6 (6.35 × 10^3).

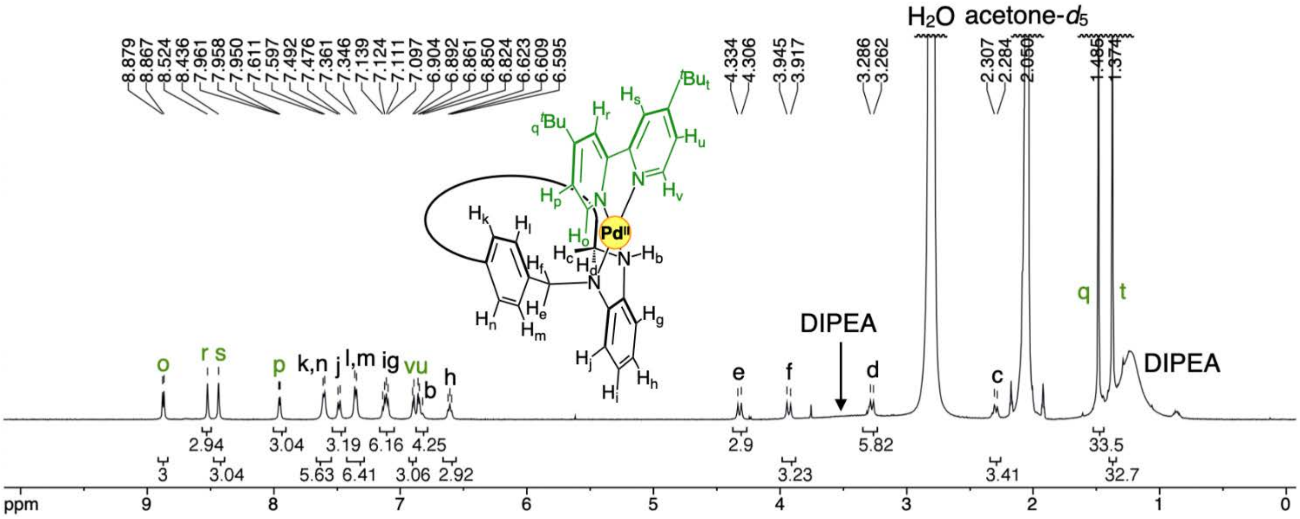

Figure S5. ^{1}H NMR spectrum of $H_3\mathbf{1}^{3+}$ deprotonated by 5.0 equiv. of DIPEA (500 MHz, acetone-d_6, 300 K). Broad signals around 1.2 and 2.5-4 ppm are the averaged signals of unreacted and protonated DIPEA.

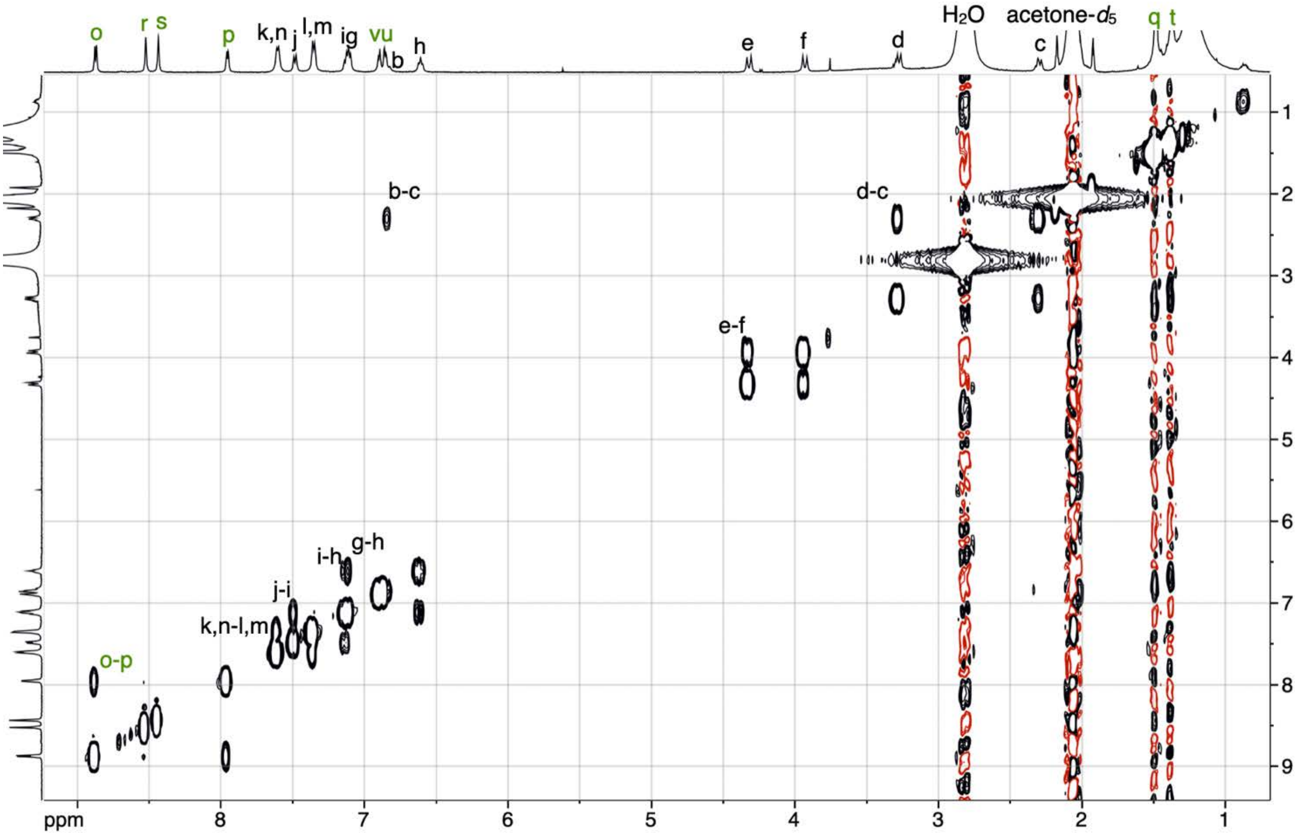

Figure S6. ^{1}H-^{1}H COSY NMR spectrum of $H_3\mathbf{1}^{3+}$ deprotonated by 5.0 equiv. of DIPEA (500 MHz, acetone-d_6, 300 K).

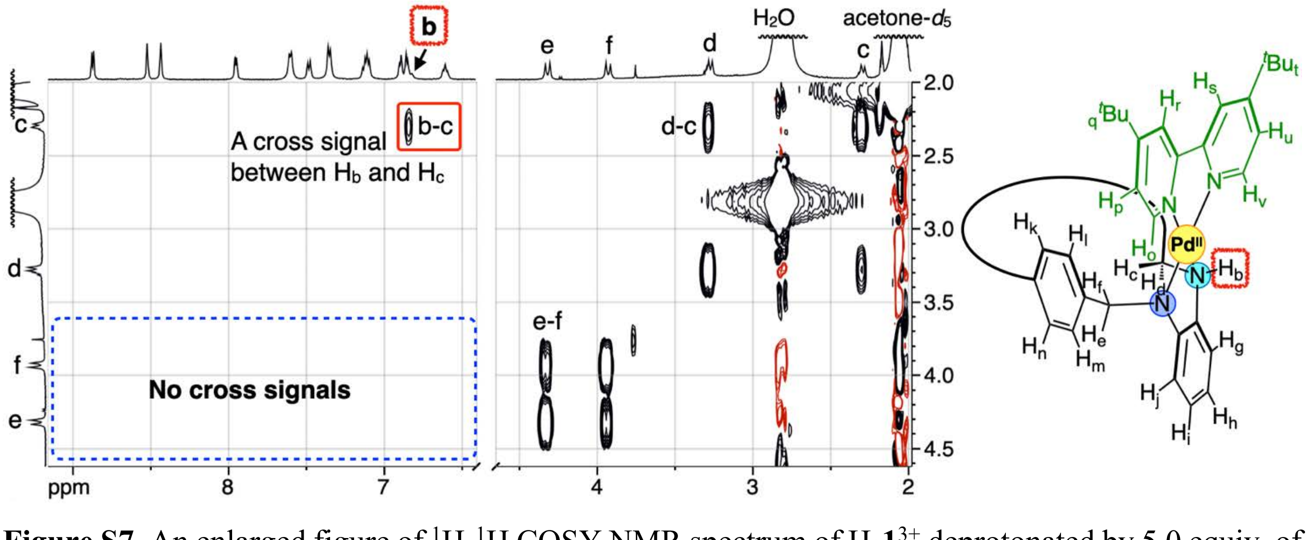

Figure S7. An enlarged figure of ^{1}H-^{1}H COSY NMR spectrum of $H_3\mathbf{1}^{3+}$ deprotonated by 5.0 equiv. of DIPEA (500 MHz, acetone-d_6, 300 K) and the chemical structural formula of the asymmetric unit.

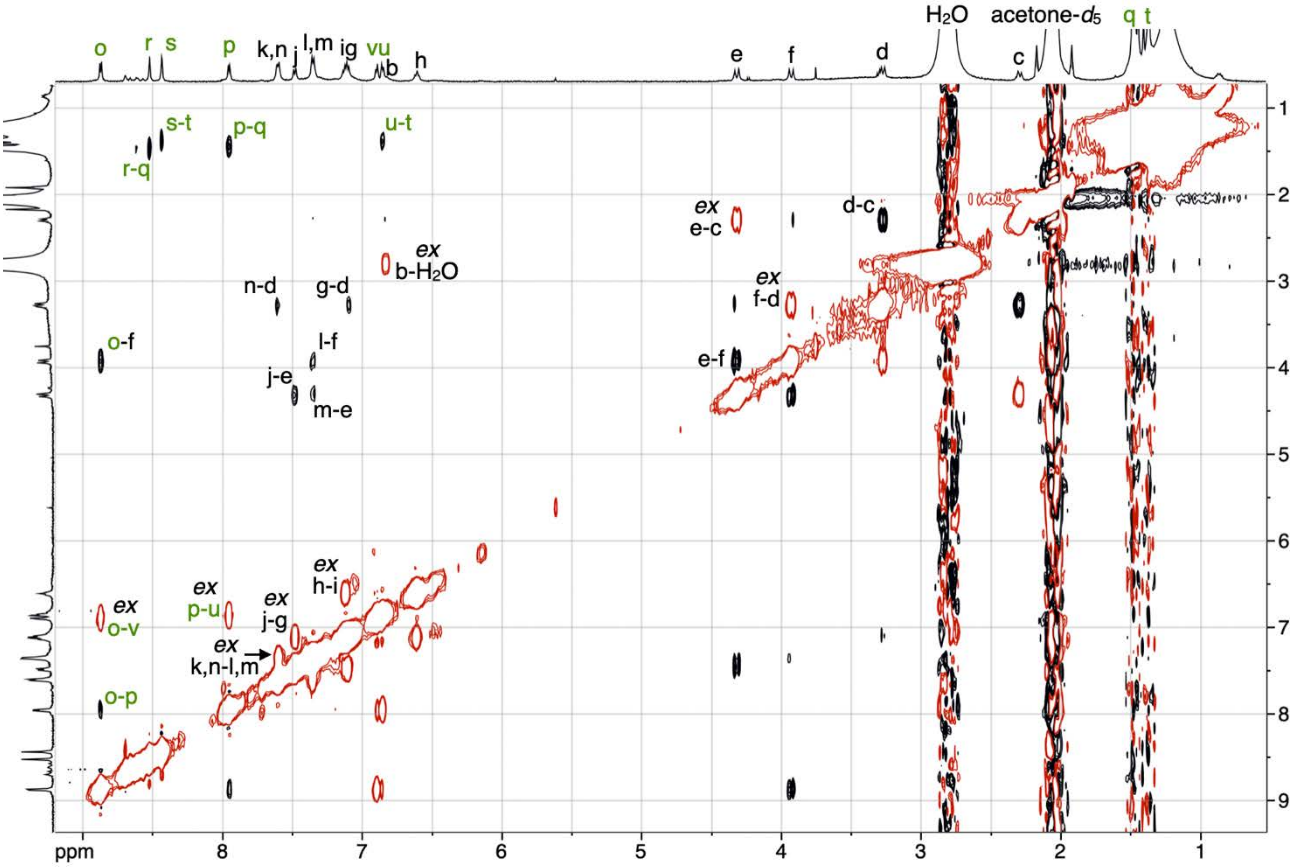

Figure S8. ^{1}H-^{1}H ROESY NMR spectrum of $H_3\mathbf{1}^{3+}$ deprotonated by 5.0 equiv. of DIPEA (500 MHz, acetone-d_6, 300 K). The symbol *ex* denotes the chemical exchange signals.

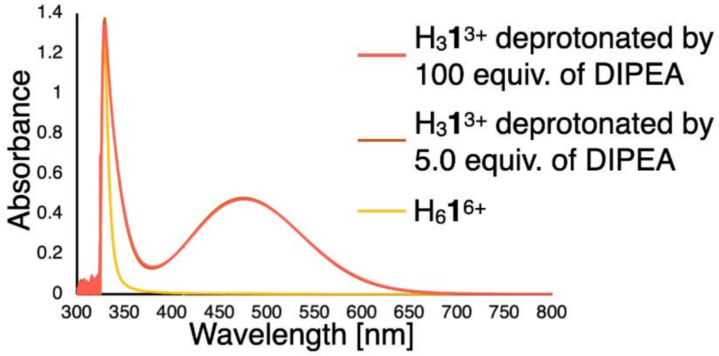

Figure S9. UV-vis spectra of (yellow line) $H_6\mathbf{1}^{6+}$, (brown line) $H_3\mathbf{1}^{3+}$ deprotonated by 5.0 equiv. of DIPEA, and (pink line) $H_3\mathbf{1}^{3+}$ deprotonated by 100 equiv. of DIPEA (acetone, 293 K, 75.7 µM, l = 1.0 cm). Note that the brown and pink lines nearly overlap. The sudden decrease of the absorbance around 320 nm is due to the absorption of acetone.

2.3 Titration of DIPEA to $H_6\mathbf{1}\cdot6OTf$ in acetone and estimation of the relative acid dissociation constant of $H_6\mathbf{1}^{6+}$

2.3.1 Theoretical analysis of the relative acid dissociation constant of $H_6\mathbf{1}^{6+}$

$$H_6\mathbf{1}^{6+} + 3\mathrm{DIPEA} \overset{K}{\rightleftharpoons} H_3\mathbf{1}^{3+} + 3[\mathrm{DIPEA}\cdot H^+]$$

We have confirmed that only three of the six amine protons of $H_6\mathbf{1}^{6+}$ were deprotonated with DIPEA as a base. Based on this finding, here we consider the equilibrium shown in the scheme above, and for simplicity, each step forming mono- and di-deprotonated Pd_3 complexes was not considered separately. In this model, the equilibrium constant *K* is given below:

$$K = \frac{[\mathrm{H_3}\mathbf{1}^{3+}][\mathrm{BH}^+]^3}{[\mathrm{H_6}\mathbf{1}^{6+}][\mathrm{B}]^3} \qquad \cdots(2\text{-}1)$$

where $[\mathrm{H_6}\mathbf{1}^{6+}]$ and $[\mathrm{H_3}\mathbf{1}^{3+}]$ are the concentration of $H_6\mathbf{1}^{6+}$ and $H_3\mathbf{1}^{3+}$, respectively, and $[\mathrm{B}]$, $[\mathrm{BH}^+]$ are the concentration of a base and its protonated base, DIPEA and $[\mathrm{DIPEA}\cdot H^+]$, respectively. During the titration experiment, the total amount of Pd complexes was constant to the initial concentration, C_0, and the total amount of the added DIPEA ($[\mathrm{B}_{tot}]$) was the sum of $[\mathrm{B}]$ and $[\mathrm{BH}^+]$, which leads to the equations (2-2) and (2-3).

$$C_0 = [\mathrm{H_6}\mathbf{1}^{6+}] + [\mathrm{H_3}\mathbf{1}^{3+}] \qquad \cdots(2\text{-}2)$$

$$[\mathrm{B}_{tot}] = [\mathrm{B}] + [\mathrm{BH}^+] \qquad \cdots(2\text{-}3)$$

From the charge balance, the equation (2-4) is given below:

$$[\mathrm{BH}^+] = 3[\mathrm{H_3}\mathbf{1}^{3+}] \qquad \cdots(2\text{-}4)$$

Substitution of equations (2-2) to (2-4) into equation (2-1) gives a quartic equation in $[\mathrm{H_3}\mathbf{1}^{3+}]$ (2-5).

$$27(K-1)[\mathrm{H_3}\mathbf{1}^{3+}]^4 - 27K(C_0 + [\mathrm{B}_{tot}])[\mathrm{H_3}\mathbf{1}^{3+}]^3 + 9K(3C_0[\mathrm{B}_{tot}] + [\mathrm{B}_{tot}]^2)[\mathrm{H_3}\mathbf{1}^{3+}]^2$$
$$-K(9C_0 + [\mathrm{B}_{tot}])[\mathrm{B}_{tot}]^2[\mathrm{H_3}\mathbf{1}^{3+}] + KC_0[\mathrm{B}_{tot}]^3 = 0 \qquad \cdots(2\text{-}5)$$

After solving this quartic equation, the concentrations of all species are obtained.

The *K* value was estimated by curve fitting analysis of titration UV-vis spectroscopy based on equation (2-6). In the simplified deprotonation model, the absorbances of the intermediary mono- and di-deprotonated Pd_3 complexes were not considered. The error of the fitting analysis was calculated as a standard error.

$$Abs. = \varepsilon'\, l[\mathrm{H_6}\mathbf{1}^{6+}] + \varepsilon l[\mathrm{H_3}\mathbf{1}^{3+}] \qquad \cdots(2\text{-}6)$$

2.3.2 Titration of DIPEA to $H_6\mathbf{1}\cdot6OTf$ in acetone

DIPEA was titrated to an acetone solution of $H_6\mathbf{1}\cdot6OTf$ to estimate the relative acid dissociation constant (*K*) of $H_6\mathbf{1}^{6+}$ to DIPEA.

$H_6\mathbf{1}\cdot6OTf$ (1.12 mg, 0.41 μmol) was dissolved in acetone (5 mL, 82 μM). 4.0 mL of this acetone solution was transferred to a quartz UV-vis cell and UV-vis spectroscopy of this solution was performed. To this solution was added an acetone solution of DIPEA (2.5 μL, 25 mM, 0.064 μmol,

0.19 equiv.), and UV-vis spectroscopy was performed. The addition of DIPEA and measurement of UV-vis spectra were repeated until the absorption around 470 nm was saturated.

After plotting the absorbance at 476.2 nm versus the equivalents of DIPEA to the Pd_3 complex, curve fitting analysis was performed based on equation (2-6) where the absorption coefficients of $H_6\mathbf{1}^{6+}$ and $H_3\mathbf{1}^{3+}$ at 476.2 nm were substituted for ε' and ε, respectively. As a result, the relative dissociation constant based on this equilibrium was estimated to be 4300 ± 800. Assuming that the dissociation constants of each deprotonation step are equal, then the acidity of one N-H group of the Pd_3 complex is approximately 16 times higher than that of the conjugate acid of DIPEA under this condition.

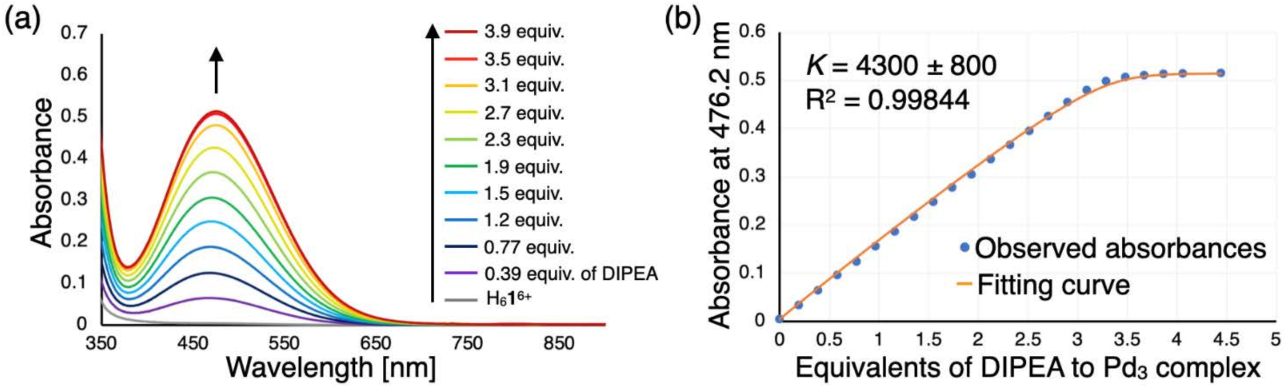

Figure S10. (a) UV-vis spectra of Pd_3 complex upon titration of DIPEA (acetone, 293 K, l = 1.0 cm, 82 μM). (b) Plot of the absorbance of Pd_3 complex at 476.2 nm versus equivalents of DIPEA to Pd_3 complex: (blue points) measured absorbances and (orange line) fitting curve based on the equation (2-6).

2.4 Reversible transformation between $H_6\mathbf{1}^{6+}$ and $H_3\mathbf{1}^{3+}$ upon alternate addition of base and acid

To check reversibility between $H_6\mathbf{1}^{6+}$ and $H_3\mathbf{1}^{3+}$, DIPEA and TfOH were added to an acetone-d_6 solution of $H_6\mathbf{1}\cdot 6OTf$ alternately.

$H_6\mathbf{1}\cdot 6OTf$ (0.35 mg, 0.13 μmol, 1.0 equiv.) was dissolved in acetone-d_6 and ^1H NMR spectroscopy of this solution was conducted. To this solution, an acetone-d_6 solution of DIPEA (2.0 μL, 350 mM, 5.5 equiv.) was added and the reaction solution was analyzed by ^1H NMR spectroscopy. Then, an acetone-d_6 solution of TfOH (3.0 μL, 330 mM, 7.6 equiv.) was added and the reaction solution was analyzed by ^1H NMR spectroscopy. In the same manner, 8.2 equiv. of DIPEA, 10 equiv. of TfOH, 14equiv. of DIPEA, and 15 equiv. of TfOH were added to the acetone-d_6 solution of $H_6\mathbf{1}\cdot 6OTf$ in this order, and spectral changes of each step were monitored by ^1H NMR spectroscopy.

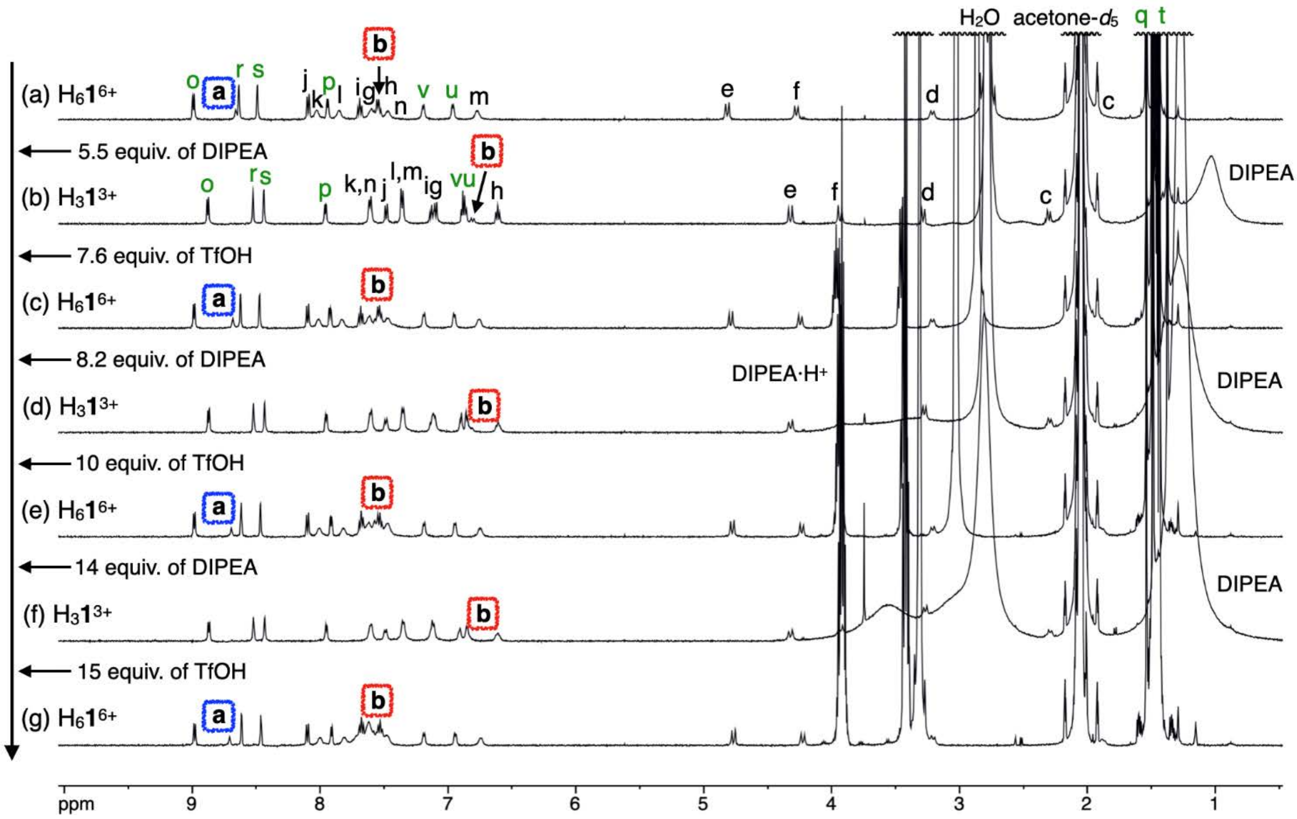

Figure S11. ^{1}H NMR spectra of $H_6\mathbf{1}^{6+}$ and $H_3\mathbf{1}^{3+}$ upon alternate addition of DIPEA and TfOH: (a) $H_6\mathbf{1}^{6+}$, (b) $H_3\mathbf{1}^{3+}$ after the addition of 5.5 equiv. of DIPEA to $H_6\mathbf{1}^{6+}$, (c) $H_6\mathbf{1}^{6+}$ after the addition of 7.6 equiv. of TfOH to the acetone-d_6 solution of (b), (d) $H_3\mathbf{1}^{3+}$ after the addition of 8.2 equiv. of DIPEA to the acetone-d_6 solution of (c), (e) $H_6\mathbf{1}^{6+}$ after the addition of 10 equiv. of TfOH to the acetone-d_6 solution of (d), (f) $H_3\mathbf{1}^{3+}$ after the addition of 14 equiv. of DIPEA to the acetone-d_6 solution of (e), and (g) $H_6\mathbf{1}^{6+}$ after the addition of 15 equiv. of TfOH to the acetone-d_6 solution of (f).

2.5 Preparation of $H_3\mathbf{1}^{3+}$ using Na_2CO_3

To an acetone-d_6 solution (0.5 mL) of $H_6\mathbf{1}\cdot 6OTf$ (0.35 mg, 0.13 µmol, 1.0 equiv.) was added solid Na_2CO_3 (0.35 mg, 3.3 µmol, 26 equiv.). This suspension was sonicated at room temperature for 4 min and centrifuged to remove precipitate. The supernatant, an acetone-d_6 solution of $H_3\mathbf{1}\cdot 3OTf$, was used for further analysis. Since NaOTf is soluble in acetone, 3 equiv. of NaOTf remained in this acetone-d_6 solution, while Na_2CO_3 and $NaHCO_3$ insoluble in acetone were removed by centrifugation.

The ^{1}H NMR spectrum of $H_3\mathbf{1}^{3+}$ prepared with Na_2CO_3 was almost identical to that of $H_3\mathbf{1}^{3+}$ prepared with DIPEA or proton sponge. Formation of $H_3\mathbf{1}\cdot 3OTf$ using Na_2CO_3 was also confirmed by high resolution-ESI-TOF mass spectrometry.

^{13}C NMR (126 MHz, acetone-d_6, 300 K): δ = 167 (supported by ^{1}H-^{13}C HMBC), 164 (supported by ^{1}H-^{13}C HMBC), 159 (supported by ^{1}H-^{13}C HMBC), 158 (supported by ^{1}H-^{13}C HMBC), 155 (supported by ^{1}H-^{13}C HMBC), 151.0, 149.2, 144 (supported by ^{1}H-^{13}C HMBC), 142 (supported by ^{1}H-^{13}C HMBC), 134 (supported by ^{1}H-^{13}C HMBC), 132.9, 130.8, 129.0, 126.1, 125.3, 124.7, 123.5, 123.4,

121.9, 121 (supported by ^{1}H-^{13}C HSQC), 121.3, 121.0, 119 (supported by ^{1}H-^{13}C HSQC), 64.6, 60.2, 36.7, 36.4, 30.8, 30.5. Some carbon signals of $H_3\mathbf{1}^{3+}$ and one TfO signal were not observed due to low solubility and were deduced using 2D ^{1}H-^{13}C HSQC and HMBC analyses. HRMS (ESI-TOF): *m*/*z* = 950.2811 as $[Pd_3(H_3\mathbf{L})(^{t}Bu_2bpy)_3(OTf)]^{2+}$ (calcd 950.2865). UV-vis (acetone, 293 K, 96.3 μM): λ_{max} (nm) (ε (M^{-1} cm^{-1})) = 465 (5.75 × 10^3).

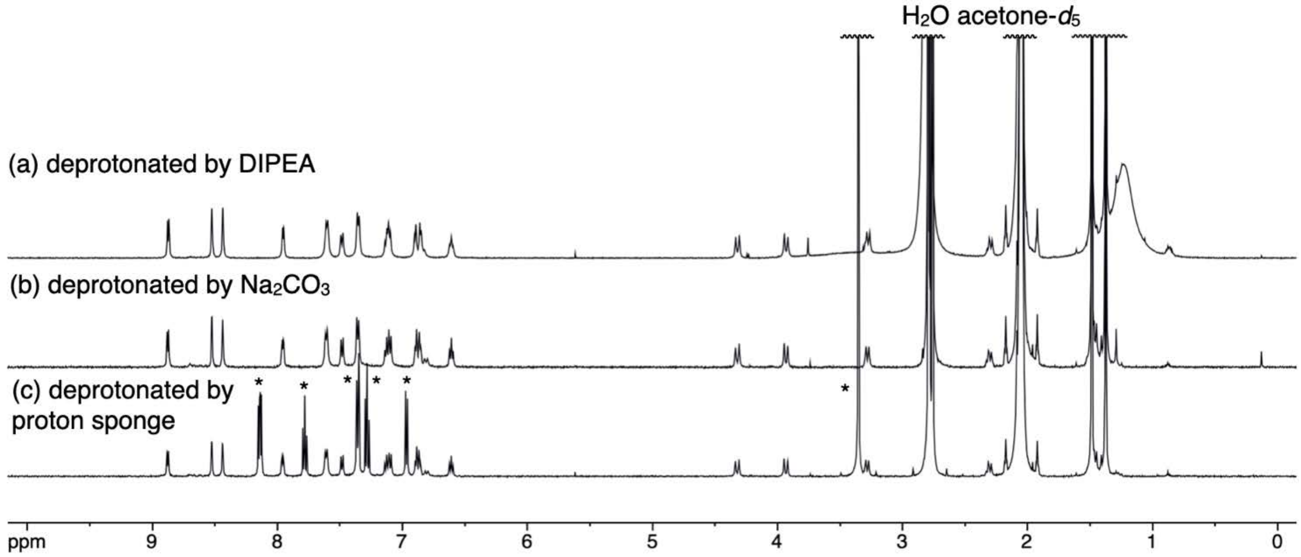

Figure S12. ^{1}H NMR spectra of $H_3\mathbf{1}^{3+}$ prepared with (a) 5.0 equiv. of DIPEA, (b) 26 equiv. of Na_2CO_3 or (c) 7.5 equiv. of proton sponge (500 MHz, acetone-d_6, 300 K). The symbol * indicates signals derived from proton sponge and its conjugate acid.

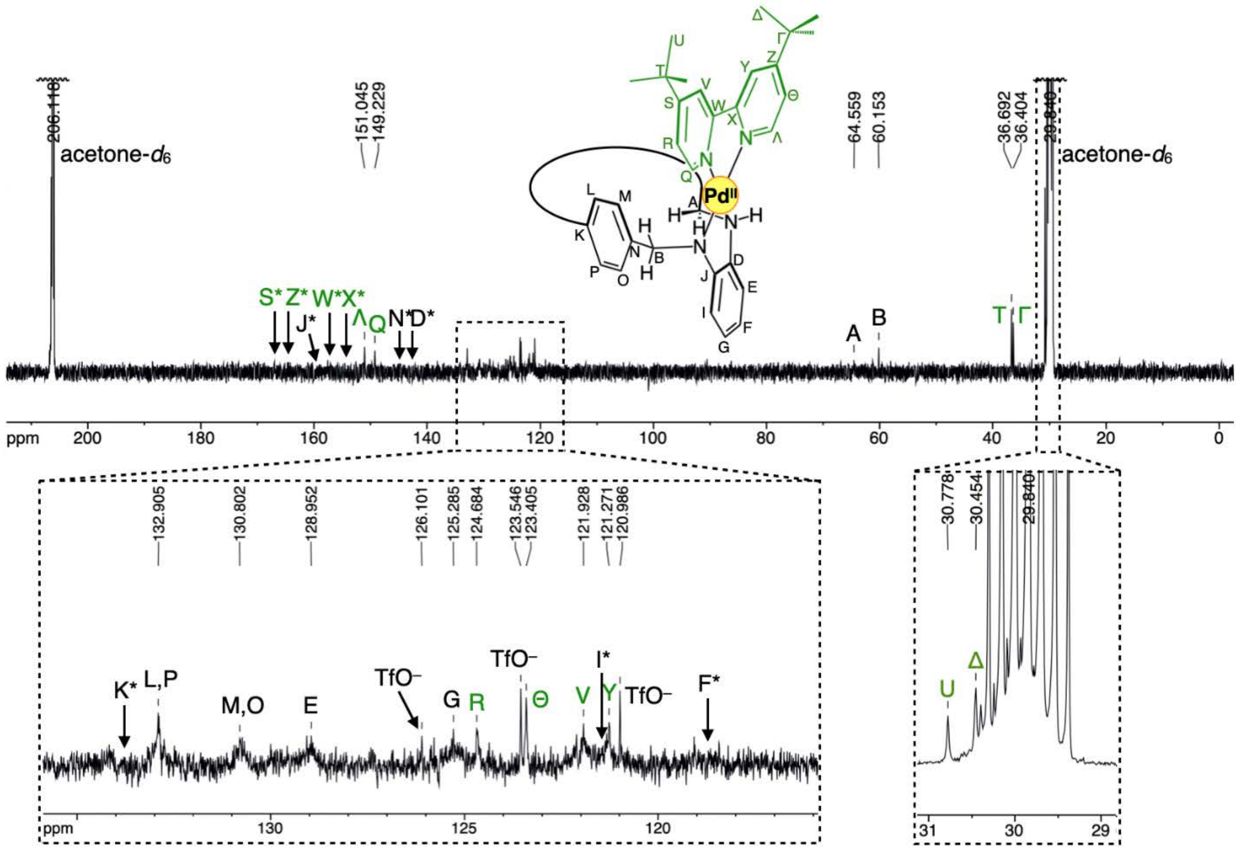

Figure S13. ^{13}C NMR spectrum of $H_3\mathbf{1}^{3+}$ prepared with Na_2CO_3 (126 MHz, acetone-d_6, 300 K). Signals with * were deduced using ^{1}H-^{13}C HSQC and HMBC NMR analyses.

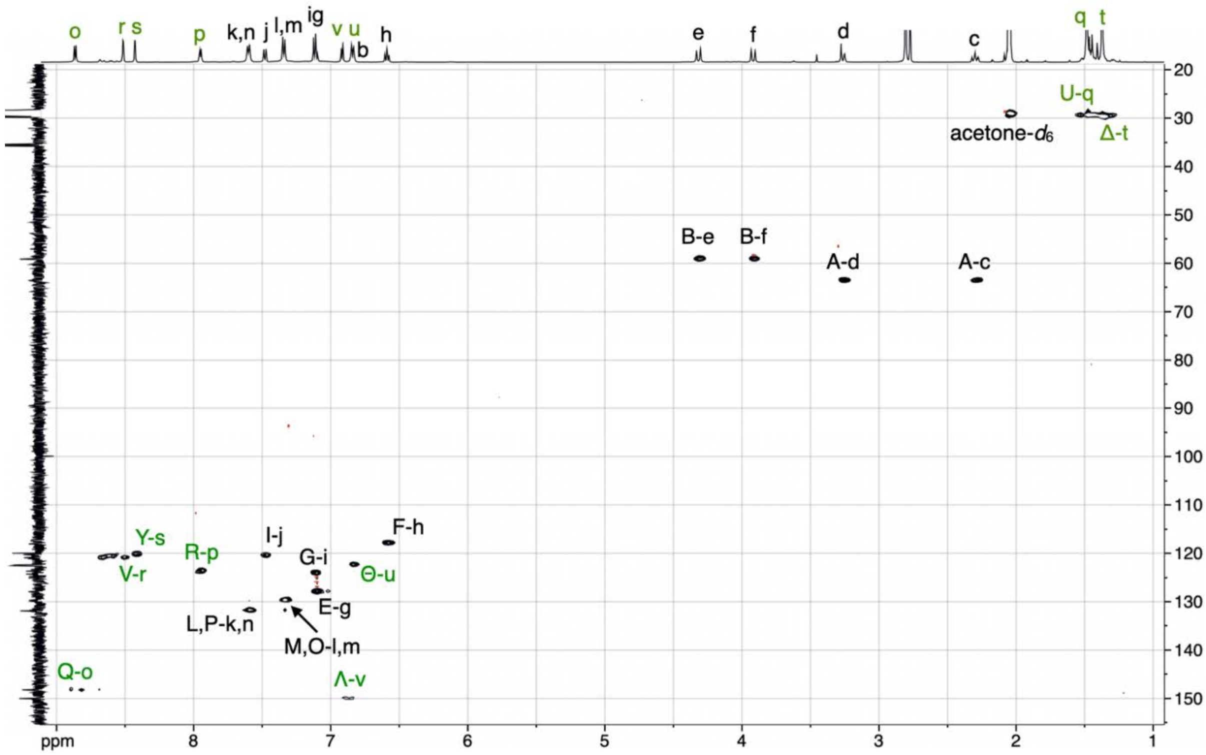

Figure S14. ^{1}H-^{13}C HSQC NMR spectrum of $H_3\mathbf{1}^{3+}$ prepared with Na_2CO_3 (500 MHz for ^{1}H and 126 MHz for ^{13}C, acetone-d_6, 300 K).

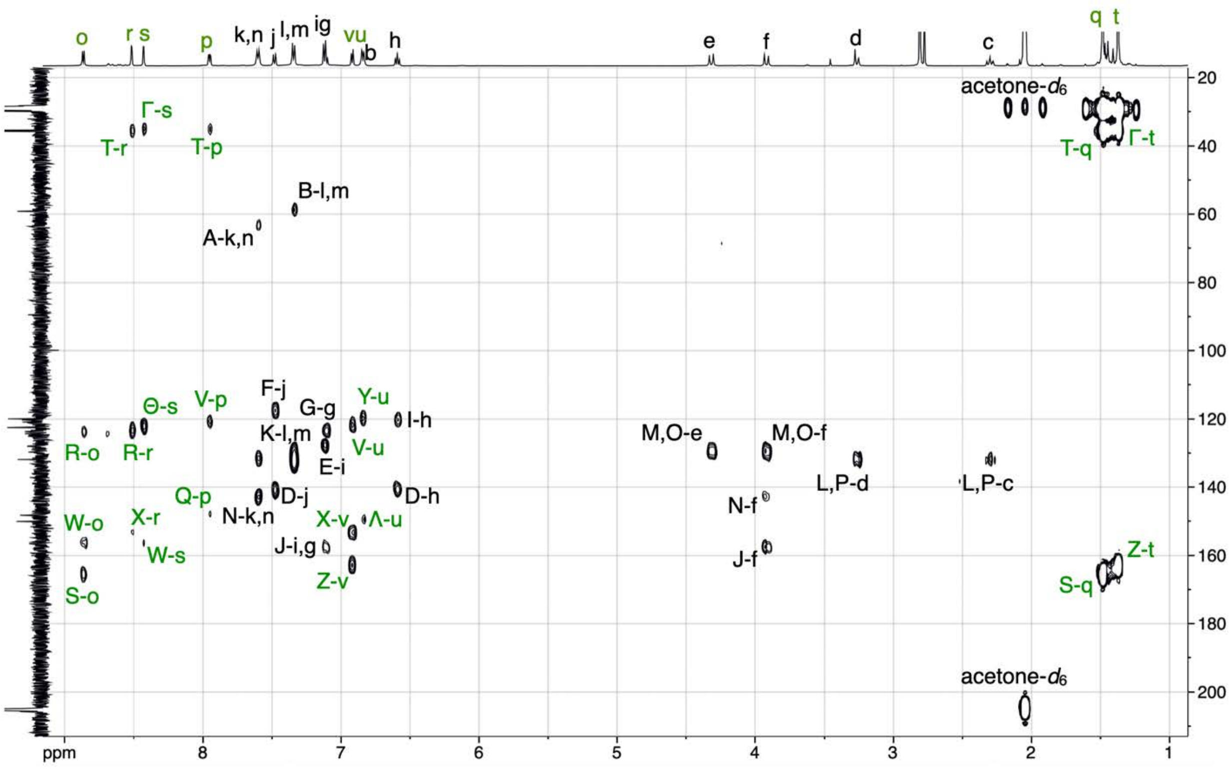

Figure S15. ^{1}H-^{13}C HMBC NMR spectrum of $H_3\mathbf{1}^{3+}$ prepared with Na_2CO_3 (500 MHz for ^{1}H and 126 MHz for ^{13}C, acetone-d_6, 300 K).

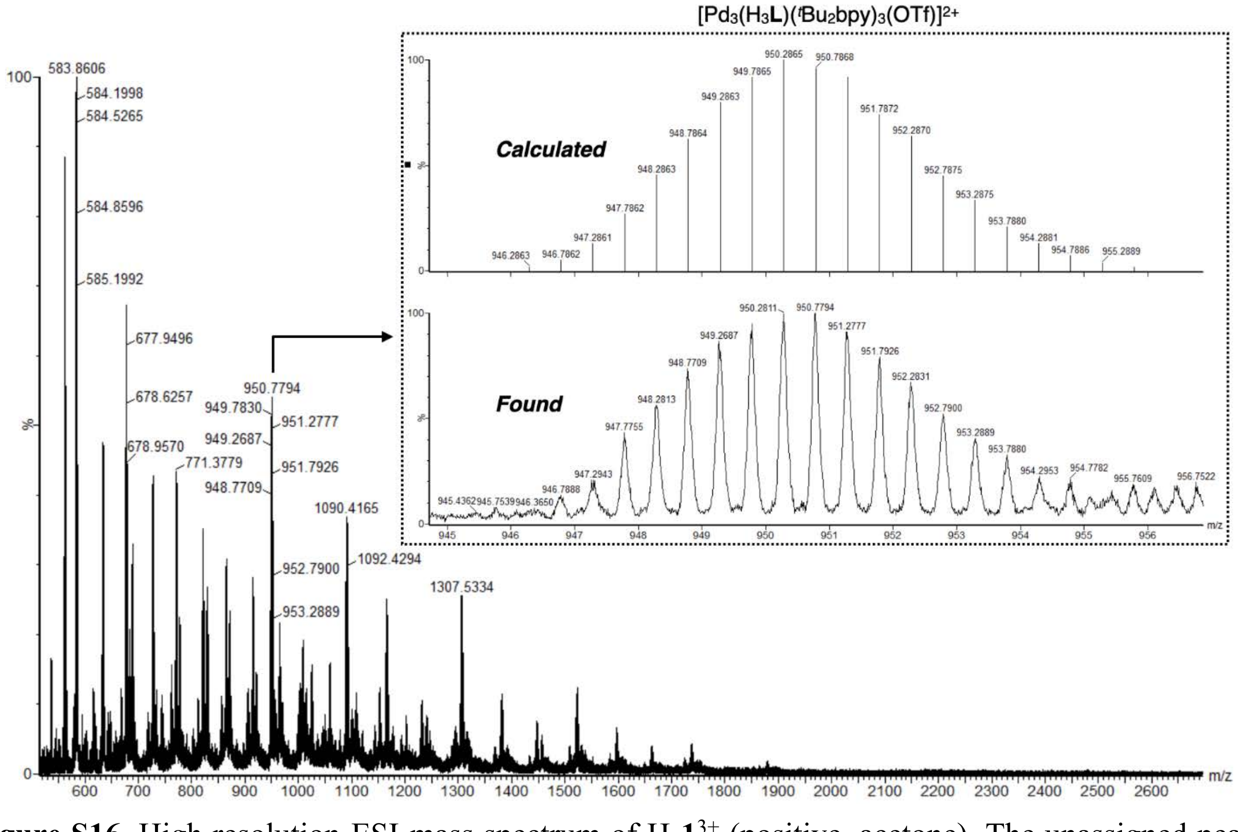

Figure S16. High resolution-ESI-mass spectrum of $H_3\mathbf{1}^{3+}$ (positive, acetone). The unassigned peaks in the higher mass ranges were possibly originated from coexisting salts such as NaOTf.

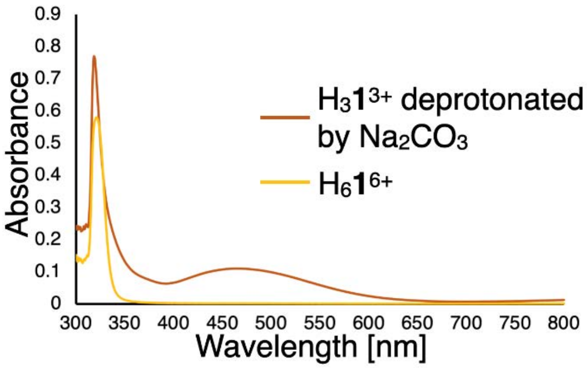

Figure S17. UV-vis spectra of (yellow line) $H_6\mathbf{1}^{6+}$ and (brown line) $H_3\mathbf{1}^{3+}$ deprotonated by Na_2CO_3 (acetone, 293 K, 96.3 μM, l = 0.2 cm). The sudden decrease of the absorbance around 320 nm is due to the absorption of acetone.

2.6 Preparation of $H_3\mathbf{1}^{3+}$ using proton sponge

To an acetone-d_6 solution (0.5 mL) of $H_6\mathbf{1}$·6OTf (0.33 mg, 0.12 μmol, 1.0 equiv.) was added solid 1,8-bis(dimethylamino)naphthalene (proton sponge, 0.12 mg, 7.5 equiv. calculated by the integral ratio in the ^{1}H NMR spectrum). The color of the solution was changed from colorless to orange. Formation of $H_3\mathbf{1}^{3+}$ was confirmed by ^{1}H NMR analysis shown in Figure S10 (c) even in the presence of a little excess amount of proton sponge.

2.7 Titration of proton sponge to $H_6\mathbf{1}$·6OTf in acetone-d_6

To confirm the deprotonation of three amine protons from $H_6\mathbf{1}^{6+}$, proton sponge was titrated to the acetone-d_6 solution of $H_6\mathbf{1}$·6OTf.

$H_6\mathbf{1}$·6OTf (0.23 mg, 0.085 μmol, 1.0 equiv.) was dissolved in acetone-d_6 (480 μL, 0.18 mM) and ^{1}H NMR spectroscopy of this solution was conducted. To this solution, an acetone-d_6 solution of proton sponge (15 mM) was titrated and ^{1}H NMR spectroscopy of the reaction solution was conducted. The equivalents of proton sponge to the Pd_3 complexes were calculated based on the integral ratio.

When less than 3 equiv. of base were added, the signals of trinuclear complexes became broadened, suggesting that proton exchange between the trinuclear complexes resulted in an equilibrium of $H_6\mathbf{1}^{6+}$, $H_5\mathbf{1}^{5+}$, $H_4\mathbf{1}^{4+}$, and $H_3\mathbf{1}^{3+}$.

The signals of unreacted proton sponge appeared in the NMR spectrum of $H_6\mathbf{1}^{6+}$, along with 2.77 equiv. of proton sponge involved in acid-base equilibrium.

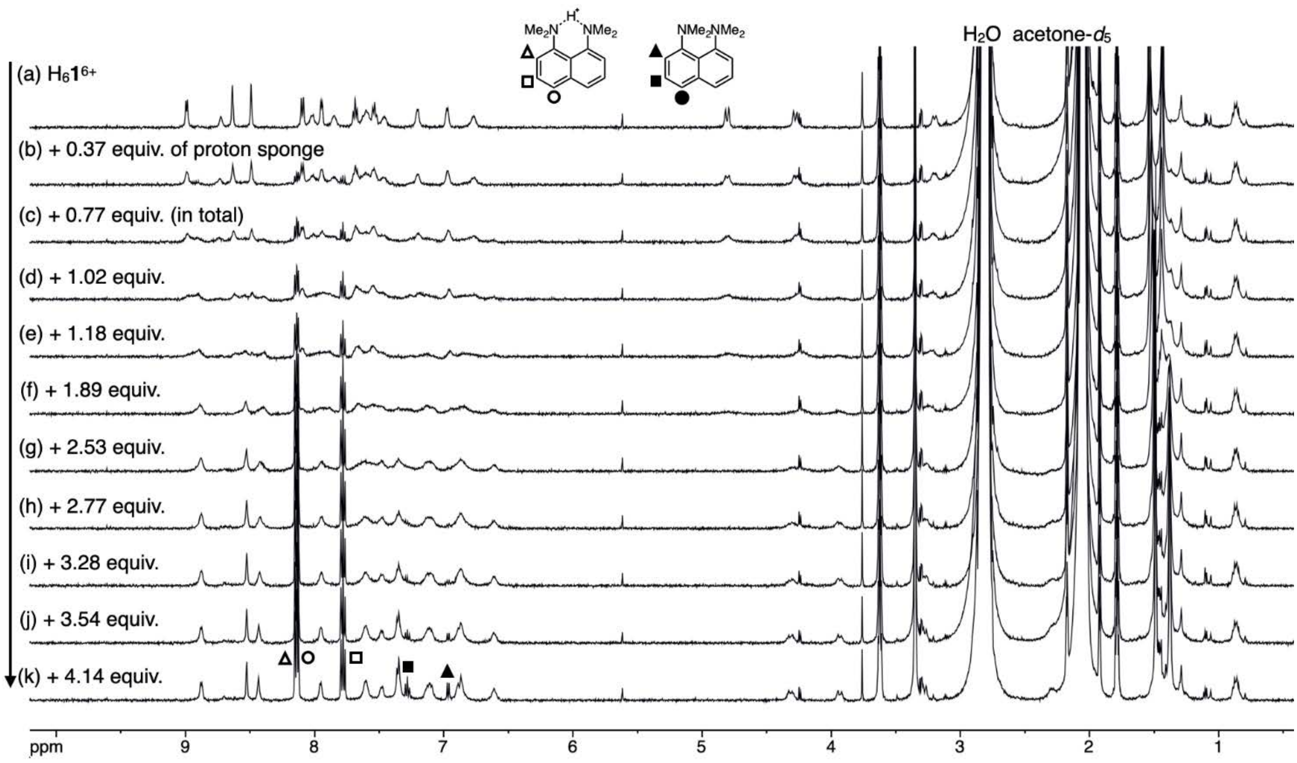

Figure S18. ^{1}H NMR spectra of $H_6\mathbf{1}^{6+}$ upon titration of proton sponge; (a) $H_6\mathbf{1}^{6+}$, (b) $H_6\mathbf{1}^{6+}$ with 0.37 equiv. of proton sponge, (c) 0.77 equiv., (d) 1.02 equiv., (e) 1.18 equiv., (f) 1.89 equiv., (g) 2.53 equiv., (h) 2.77 equiv., (i) 3.28 equiv., (j) 3.54 equiv., and (k) 4.14 equiv. (500 MHz, acetone-d_6, 300 K).

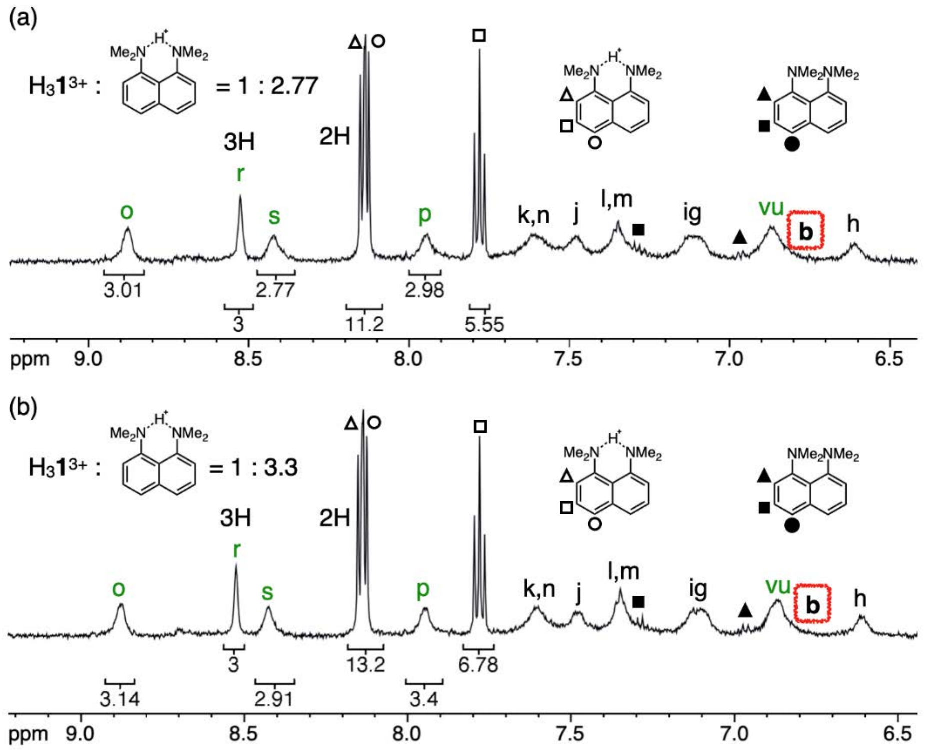

Figure S19. ^{1}H NMR spectra (500 MHz, acetone-d_6, 300 K) of $H_6\mathbf{1}^{6+}$ after addition of (a) 2.77 equiv. and (b) 3.28 equiv. of proton sponge and the molar ratio of $H_3\mathbf{1}^{3+}$ to protonated proton sponge.

2.8 Deprotonation of $H_6\mathbf{1}^{6+}$ using ^{t}BuOK as a stronger base

To check the deprotonation behavior of $H_6\mathbf{1}^{6+}$ with a stronger base, ^{t}BuOK was reacted with $H_6\mathbf{1}^{6+}$.

A dry THF suspension of ^{t}BuOK (18 mM, 60 µL, 1.1 µmol, 12 equiv.) was added to a dry THF suspension (0.8 mL) of $H_6\mathbf{1}\cdot 6OTf$ (0.09 µmol, 1.0 equiv.) and this suspension was sonicated for 7 min at room temperature under nitrogen atmosphere. After filtration to remove the precipitate, the resulting pale purple filtrate was analyzed by UV-vis-NIR spectroscopy under nitrogen atmosphere. To the pale purple solution, a dry THF solution of TfOH (118 mM, 5.0 µL, 6.5 equiv.) was added. The color of this solution was changed to colorless immediately, and the UV-vis-NIR spectroscopy of this solution was conducted.

In the UV-vis-NIR spectrum of the pale purple solution, two absorption bands around 870 and 510 nm were observed. Especially, the broad NIR absorption in this spectrum newly appeared and different from that of $H_3\mathbf{1}^{3+}$. After the addition of acid, the spectrum of the colorless solution was almost identical to that of $H_6\mathbf{1}^{6+}$, which deny the oxidation reaction during the deprotonation process. From these results, the formation of a further deprotonated complex, $H_n\mathbf{1}^{n+}$ (n = 2, 1, or 0), was suggested by the reaction of $H_6\mathbf{1}^{6+}$ with ^{t}BuOK. This is also supported by the TD-DFT calculation of **1** which reproduced the characteristic absorption spectrum (see Supplementary section 5.6 for the details of DFT calculations).

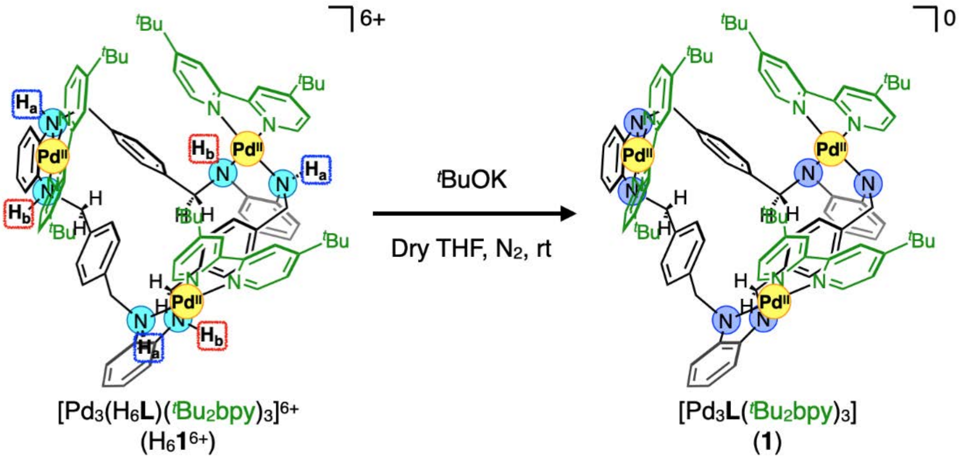

Figure S20. Scheme of the deprotonation reaction with ^{t}BuOK. The product of $[Pd_3\mathbf{L}(^t Bu_2bpy)_3]$ = **1** in the scheme was shown as a typical product.

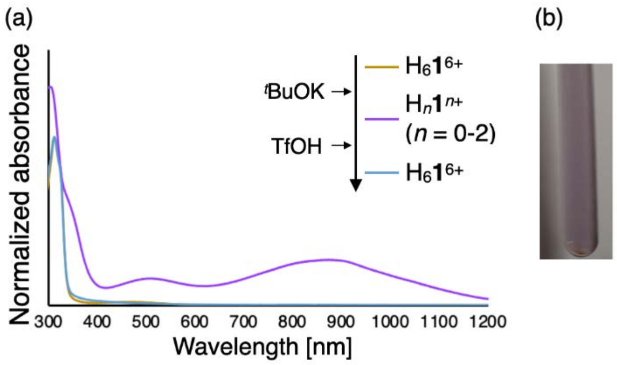

Figure S21. (a) UV-vis spectra of (yellow line) $\mathbf{1}^{6+}$, (purple line) the pale purple solution of $H_n\mathbf{1}^{n+}$ (n = 2, 1, or 0) deprotonated by ^{t}BuOK and (blue line) the colorless solution of the recovered $H_6\mathbf{1}^{6+}$ (dry THF, 293 K, l = 0.2 cm). The concentration of the solutions was not determined correctly because some of trinuclear Pd^{II} complexes were removed by filtration. (b) A picture of the pale purple solution of $H_n\mathbf{1}^{n+}$ (n = 2, 1, or 0) in THF.

3. Synthesis of model Pd complexes

3.1 Synthesis of $H_2\mathbf{2}\cdot 2OTf$

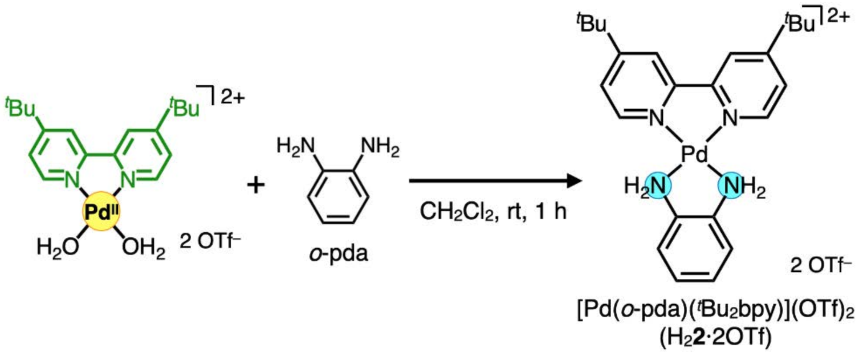

Figure S22. Synthetic scheme of [Pd(o-pda)(^{t}Bu$_2$bpy)](OTf)$_2$ = $H_2\mathbf{2}\cdot 2OTf$.

To a CH_2Cl_2 solution of [Pd(^{t}Bu$_2$bpy)(OH_2)$_2$](OTf)$_2$ (10.00 mg, 13.4 µmol, 1.0 equiv.) was added a CH_2Cl_2 solution of *ortho*-phenylenediamine (o-pda, 1.80 mg, 16.7 µmol, 1.2 equiv.). This reaction solution was stirred at room temperature for 1 h and a colorless precipitate was formed. After filtering the colorless solid was recrystallized from acetone solution by vapor diffusion of Et_2O to afford $H_2\mathbf{2}\cdot 2OTf$ as colorless crystals (7.96 mg, 10.2 µmol, 76%).
Mp: > 262 °C (decomp.).^{1}H NMR (500 MHz, acetone-d_6, 300 K): δ = 8.78 (d, J = 1.5 Hz, 2H), 8.71 (d, J = 6.0 Hz, 2H), 7.93 (dd, J = 6.0, 2.0 Hz, 2H), 7.74 (brs, 4H) 7.61 (m, 2H), 7.46 (m, 2H), 1.48 (s, 18H). ^{13}C NMR (126 MHz, acetone-d_6, 300 K): δ = 168.4, 157.1, 152.2, 139.5, 129.8, 127.5, 125.9, 125.8, 123.3, 122.7, 120.8, 37.0, 30.3. IR (ATR, cm^{-1}): 3141 (br), 3063 (br), 2966 (br), 1620, 1496, 1489, 1418, 1280, 1217, 1160, 1025, 901, 842, 780, 634. HRMS (ESI-TOF): m/z = 631.1212 as [Pd(o-

pda)(^{t}Bu$_2$bpy)](OTf)]$^+$ (calcd 631.1191). UV-vis (EtOH, 295 K, 53.0 μM): λ_{max} (nm) (ε (M^{-1} cm^{-1})) = 307.5 (1.46 × 10^4).

Crystal data for [Pd(*o*-pda)(^{t}Bu$_2$bpy)](OTf)$_2$: $C_{26}H_{32}F_6N_4O_6PdS_2$, F_w = 781.07, crystal dimensions 0.550 × 0.071 × 0.068 mm^3, monoclinic, space group $P2_1/c$, a = 11.5335(2), b = 9.42261(18), c = 29.0801(5) Å, β = 92.4753(16)°, V = 3157.35(10) Å^3, Z = 4, ρ_{calcd} = 1.643 g cm^{-3}, μ = 67.15 cm^{-1}, T = 93.15 K, λ(CuKα) = 1.54184 Å, 2θ_{max} = 136.454°, 15151/5765 reflections collected/unique (R_{int} = 0.0395), R_1 = 0.0335 ($I > 2\sigma(I)$), wR_2 = 0.0870 (for all data), GOF = 1.036, largest diff. Peak and hole 0.741/–0.703 eÅ^{-3}. CCDC deposit number 2315860.

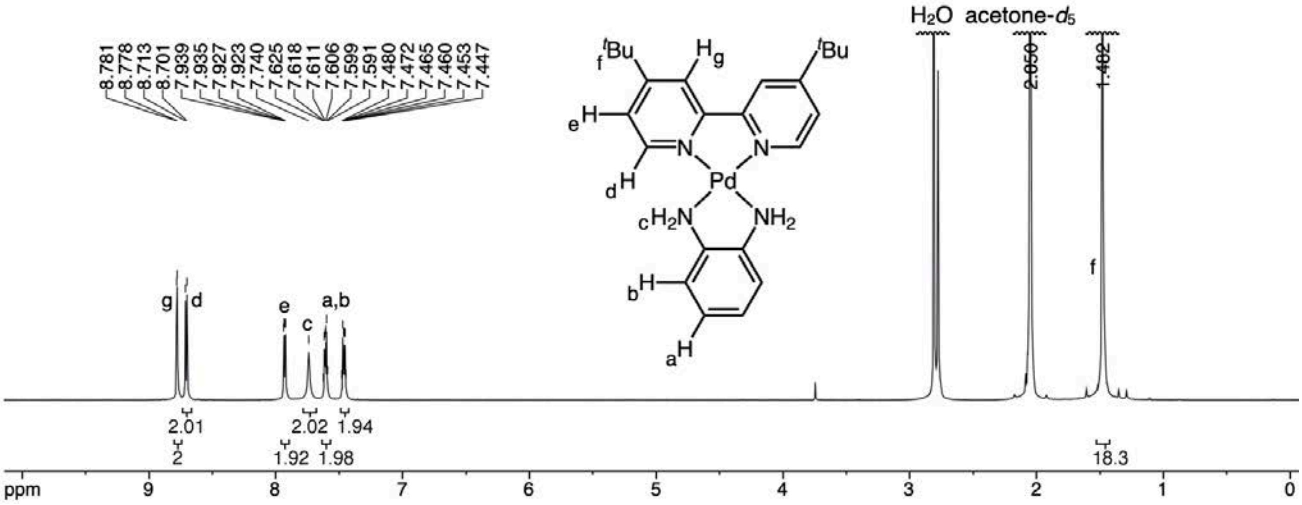

Figure S23. ^{1}H NMR spectrum of $H_2\mathbf{2}^{2+}$ (500 MHz, acetone-d_6, 300 K).

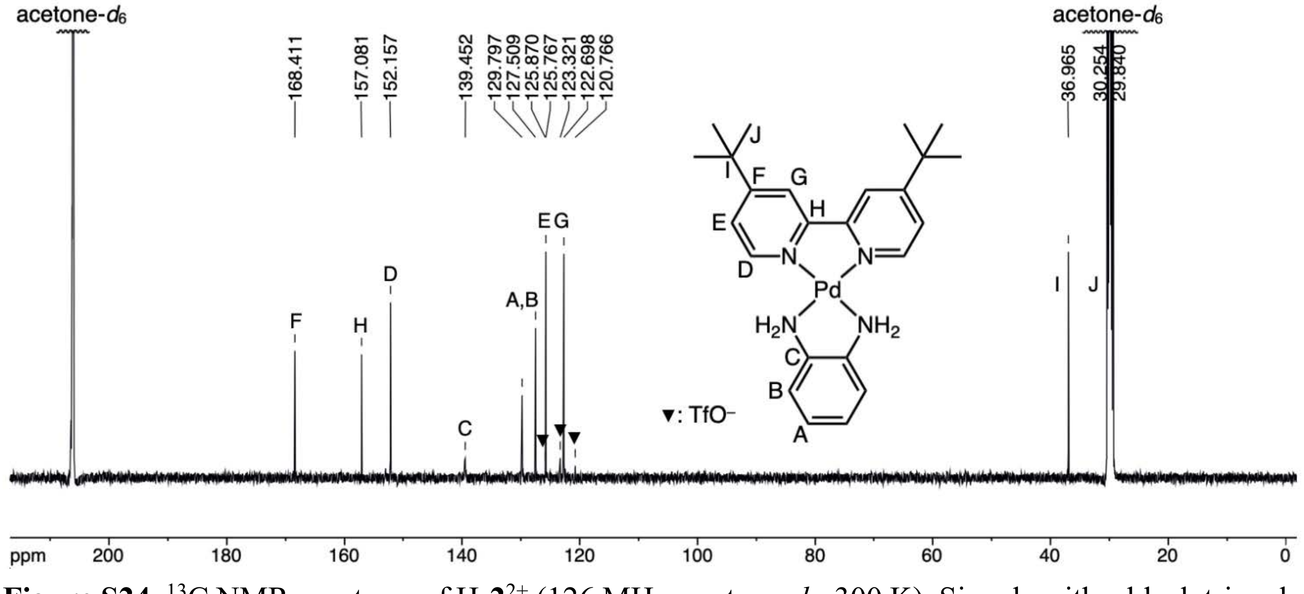

Figure S24. ^{13}C NMR spectrum of $H_2\mathbf{2}^{2+}$ (126 MHz, acetone-d_6, 300 K). Signals with a black triangle are assigned to TfO$^–$.

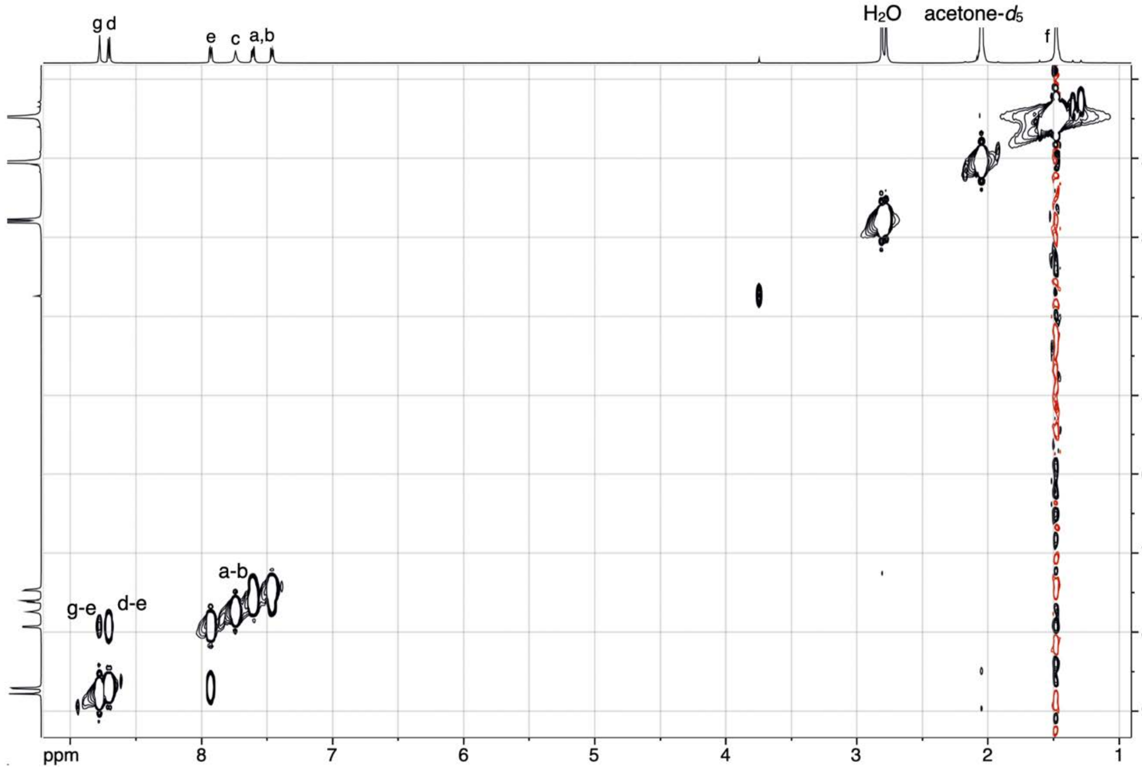

Figure S25. ^{1}H-^{1}H COSY NMR spectrum of $H_2\mathbf{2}^{2+}$ (500 MHz, acetone-d_6, 300 K).

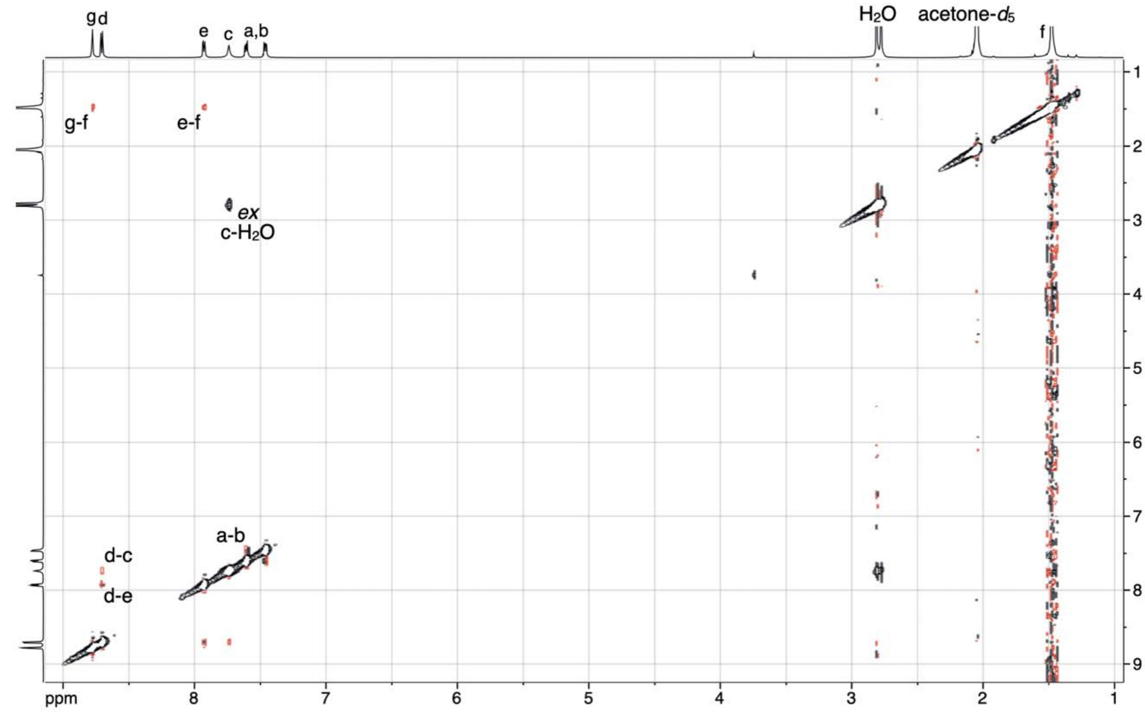

Figure S26. ^{1}H-^{1}H NOESY NMR spectrum of $H_2\mathbf{2}^{2+}$ (500 MHz, acetone-d_6, 300 K).

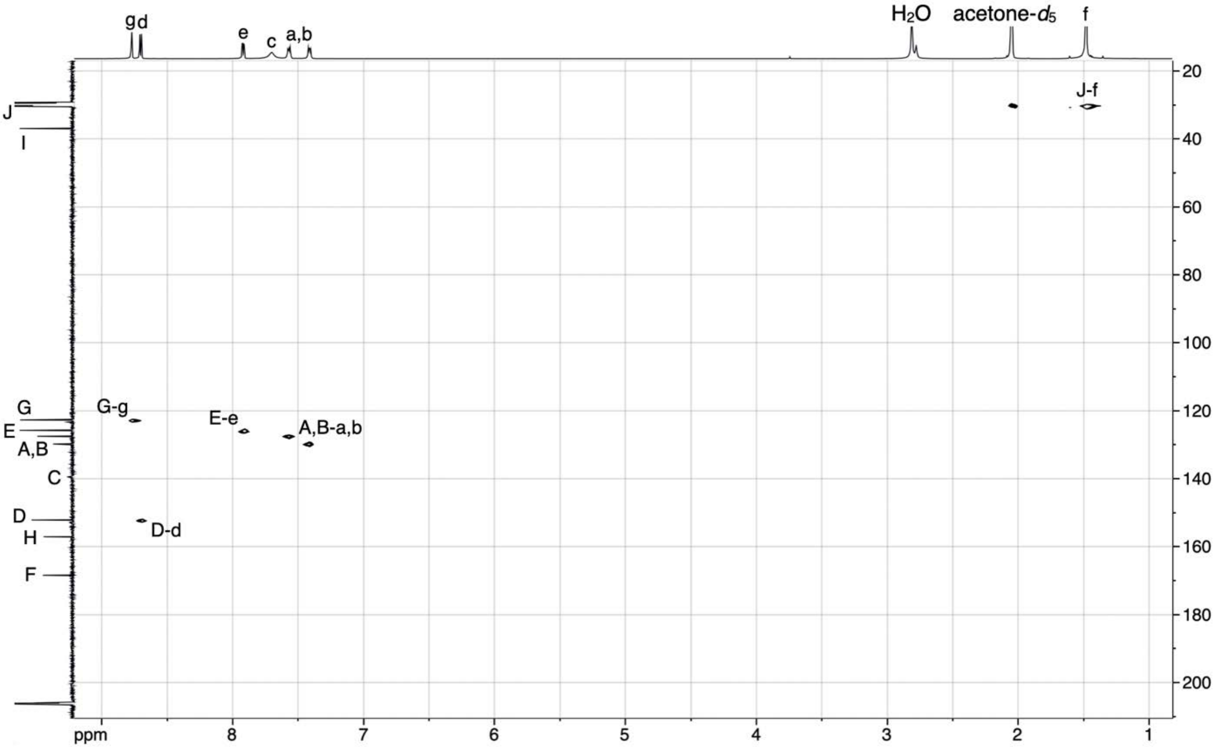

Figure S27. ^{1}H-^{13}C HSQC NMR spectrum of $H_2\mathbf{2}^{2+}$ (500 MHz for ^{1}H and 126 MHz for ^{13}C, acetone-d_6, 300 K).

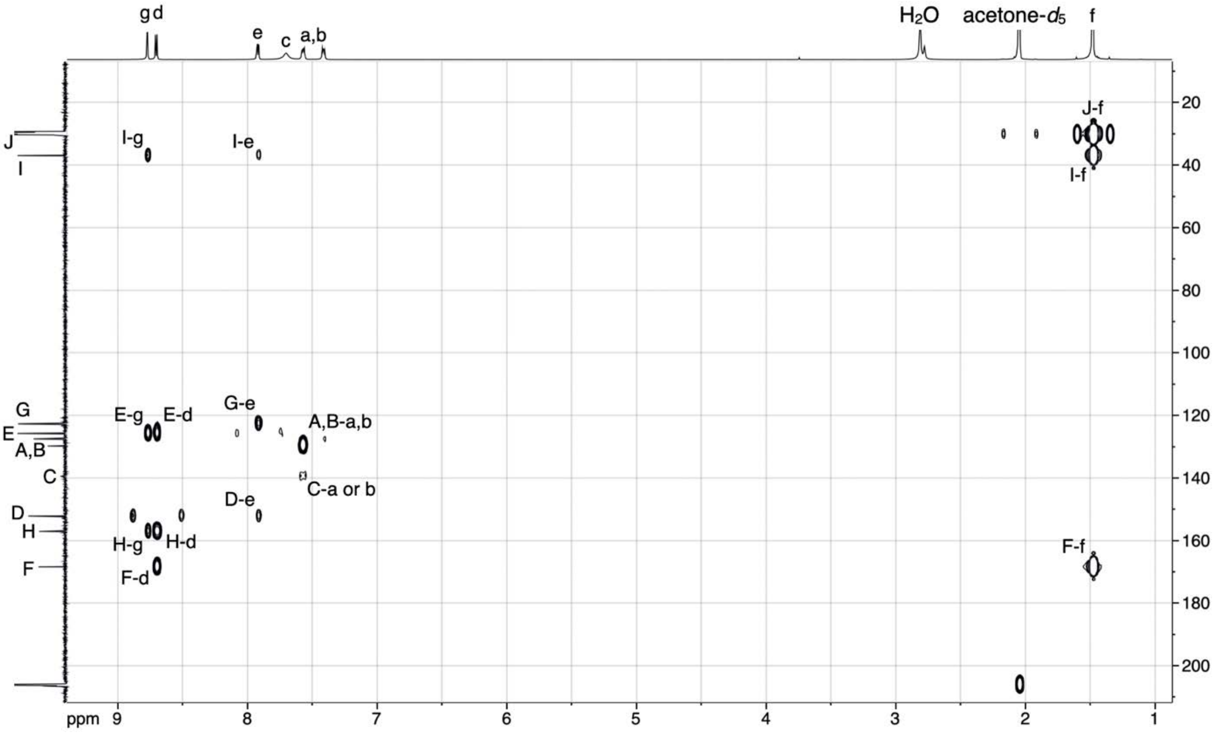

Figure S28. ^{1}H-^{13}C HMBC NMR spectrum of $H_2\mathbf{2}^{2+}$ (500 MHz for ^{1}H and 126 MHz for ^{13}C, acetone-d_6, 300 K).

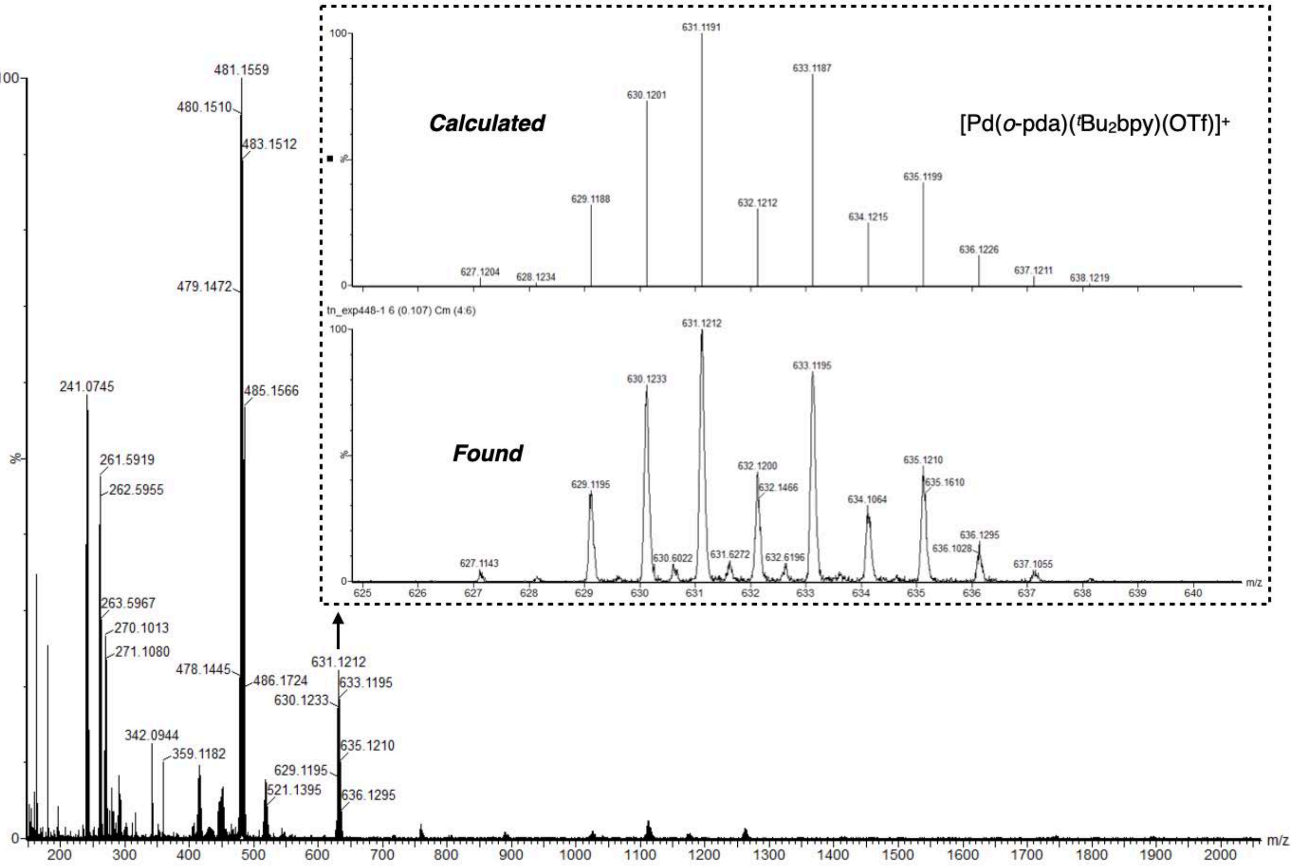

Figure S29. High-resolution ESI-mass spectrum of $H_2\mathbf{2}^{2+}$ (positive, acetone).

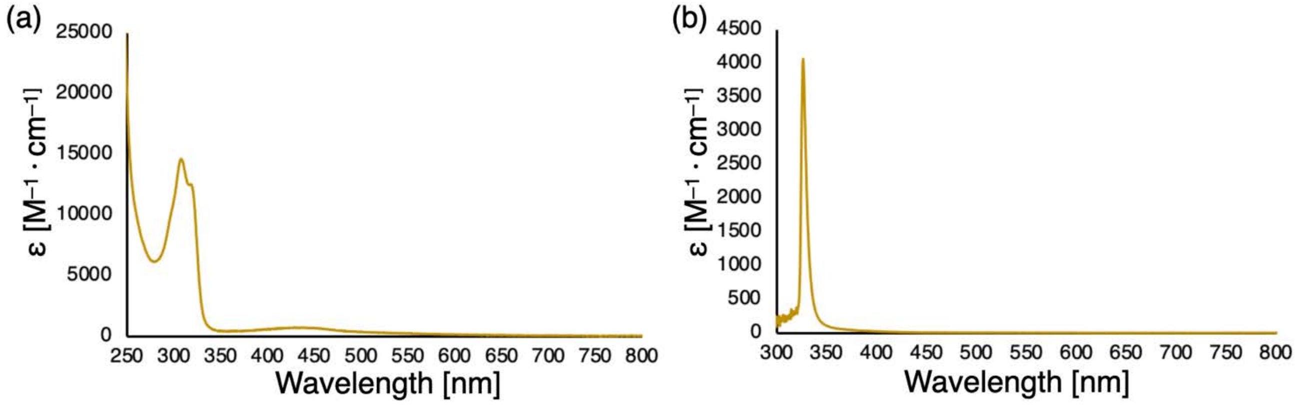

Figure S30. UV-vis spectrum of $H_2\mathbf{2}^{2+}$ (a) in EtOH (295 K, 53.0 µM, l = 0.2 cm). Because of the high acidity of amine proton in EtOH, $H_2\mathbf{2}^{2+}$ had an absorption around 430 nm in the UV-vis spectrum measured in EtOH. (b) in acetone (293 K, 259 µM, l = 1.0 cm). The sudden decrease of the absorbance around 320 nm is due to the absorption of acetone.

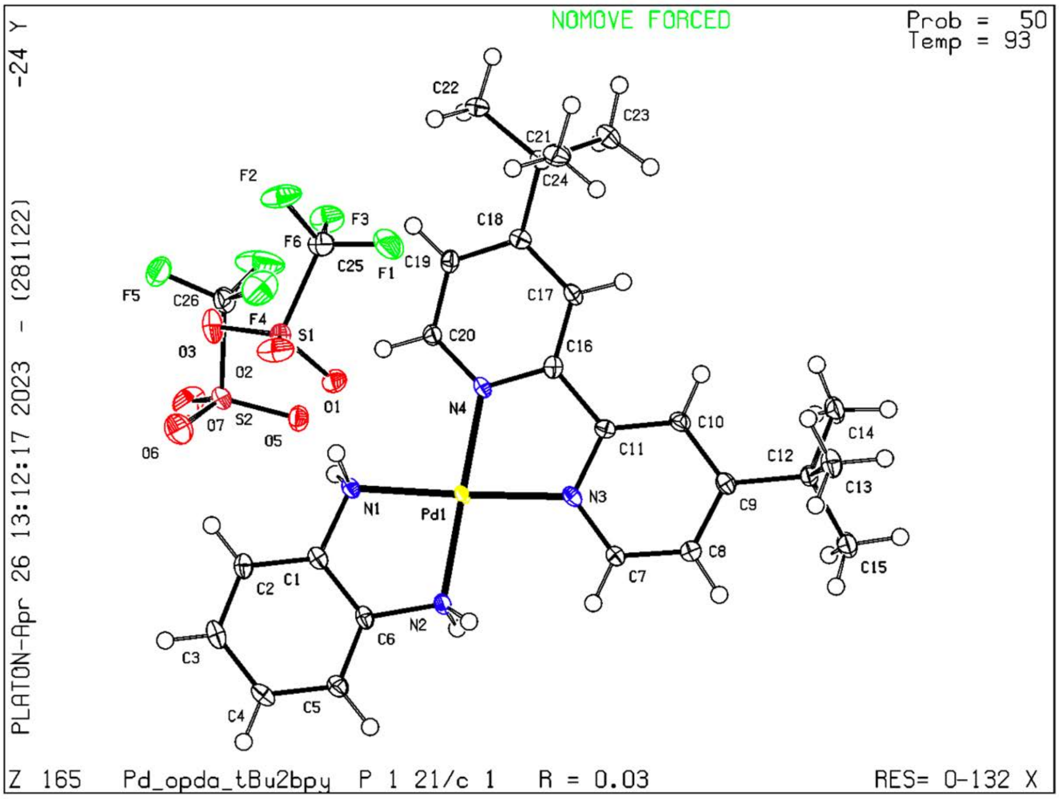

Figure S31. ORTEP drawing of H_2**2**·2OTf at the 50% probability level. Color: C black, N blue, O red, F yellow-green, S dark red, and Pd yellow. CCDC deposit number of H_2**2**·2OTf is 2315860. This figure was produced by the checkCIF report of the International Union of Crystallography.

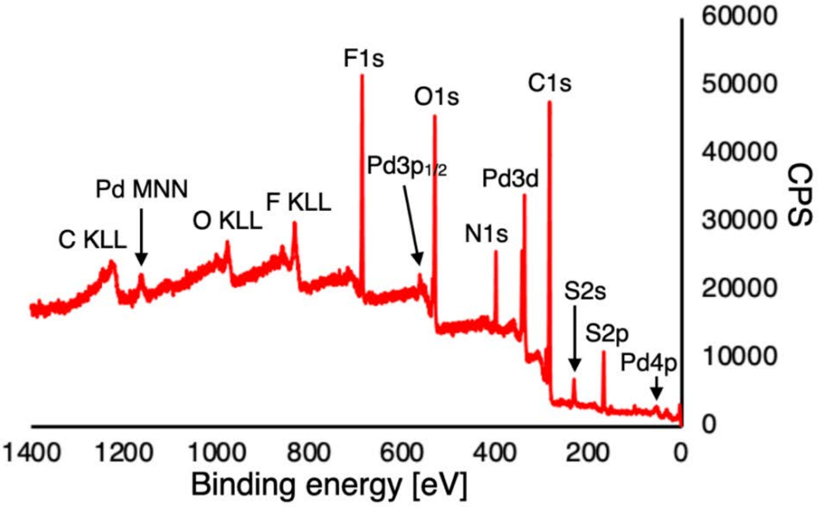

Figure S32. XPS spectrum (survey scan) of H_2**2**·2OTf.

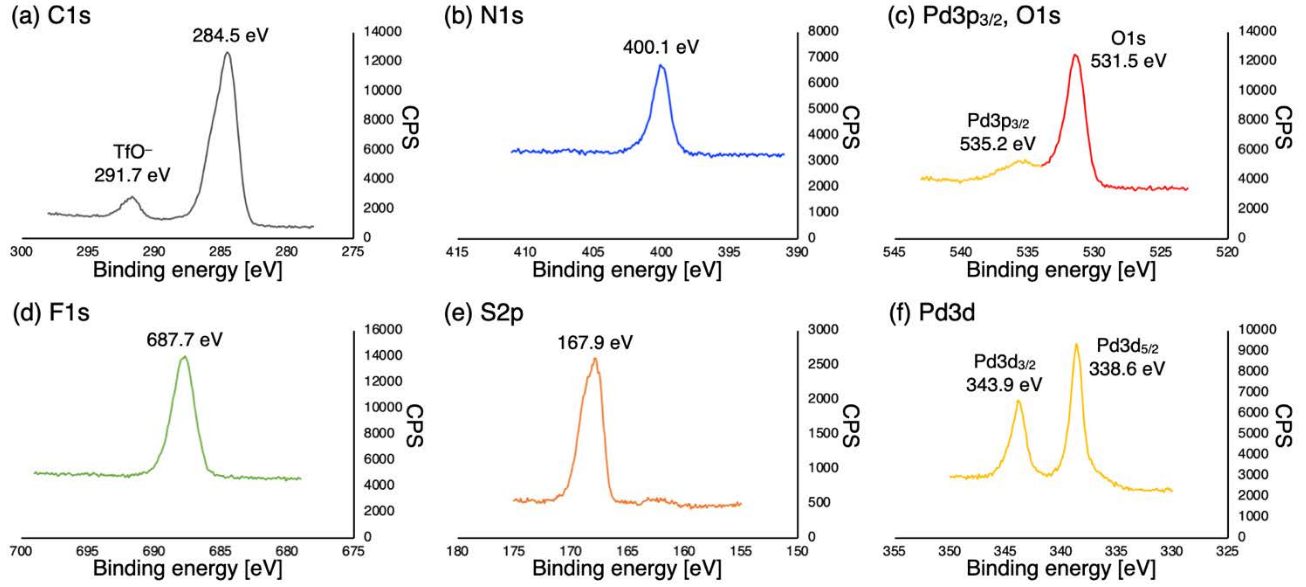

Figure S33. XPS spectra of (a) C1s, (b) N1s, (c) Pd3p3/2 and O1s, (d) F1s, (e) S2p and (f) Pd3d of H_2**2**·2OTf.

	C1s	N1s	O1s	F1s	S2p	Pd3d
Observed ratio	29.5	3.9	7.4*	6.4	2.0	1.0
Theoretical chemical formula	26	4	6	6	2	1

Figure S34. Table of the ratio of each element measured by XPS of H_2**2**·2OTf. *: The ratio of O1s was analyzed by deconvolution because of the overlapping with the Pd3p3/2 signal.

3.2 Synthesis of H2·OTf

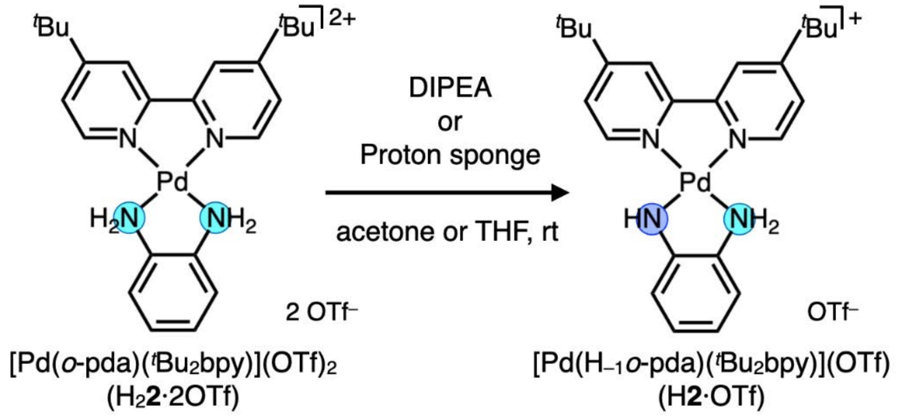

Figure S35. Synthetic scheme of [Pd(H_{-1}*o*-pda)(^{t}Bu$_2$bpy)](OTf) = H**2**·OTf.

To an acetone solution of H_2**2**·2OTf (11.86 mg, 15.2 µmol, 1.0 equiv.) was added DIPEA (3.2 µL, 18.4 µmol, 1.2 equiv.). The color of this solution was changed from colorless to orange. H**2**·OTf was crystallized by vapor diffusion of Et_2O in a refrigerator. After drying *in vacuo*, orange crystals of H**2**·OTf were obtained (8.07 mg, 12.8 µmol, 84%).

Orange crystals of H$\mathbf{2}^+$ were also prepared by the addition of proton sponge shown below. To an acetone solution (5 mL) of H_2**2**·2OTf (9.36 mg, 12.0 µmol, 1.0 equiv.) was added solid proton sponge (3.08 mg, 12.8 µmol, 1.1 equiv.). After filtering, H**2**·OTf was crystallized by vapor diffusion of Et_2O in an explosion-proof refrigerator to afford orange crystals (5.94 mg, 9.41 µmol, 79%).

It was confirmed by ^{1}H NMR spectroscopy that the orange crystals of H**2**·OTf deprotonated by both DIPEA and proton sponge were chemically identical. The obtained H**2**·OTf was analyzed by 1D ^{1}H and ^{13}C, 2D ^{1}H-^{1}H COSY and NOESY, ^{1}H-^{13}C HSQC and HMBC NMR spectroscopy, high-resolution ESI-TOF mass spectrometry, UV-vis spectroscopy, IR spectroscopy, and elemental analysis.

Single-crystal XRD analysis was performed on a crystal separately prepared by the following procedure. H_2**2**·2OTf (11.71 mg, 15.0 µmol, 1.0 equiv.) was dissolved in THF. To this solution, DIPEA (3.4 µL, 19.5 µmol. 1.3 equiv.) was slowly added. Then, this solution was stood at room temperature overnight to afford red-orange needle crystals of H**2**·OTf (3.17 mg, 5.0 µmol, 34%), which was analyzed by single-crystal XRD.

To confirm that these two crystallization procedures afforded crystallographically identical crystals of H**2**·OTf, powder XRD (pXRD) analyses of H**2**·OTf crystallized in both procedures were conducted.

Mp: > 204 °C (decomp.).^{1}H NMR (500 MHz, acetone-d_6, 300 K): δ = 8.73 (d, J = 6.0 Hz, 2H), 8.70 (d, J = 2.0 Hz, 2H), 7.85 (dd, J = 6.0, 2.0 Hz, 2H), 6.74 (m, 2H), 6.51 (m, 2H), 6.09 (brs, 3H), 1.47 (s, 18H). ^{13}C NMR (126 MHz, acetone-d_6, 300 K): δ = 166.7, 156 (supported by ^{1}H-^{13}C HMBC), 150.9, 125.3, 122.0, 121 (supported by ^{1}H-^{13}C HSQC), 36.7, 30.1. The signals of TfO^- and one quaternary carbon of $[H_{-1}\mathbf{2}]^+$ were not observed due to low solubility. IR (ATR, cm^{-1}): 3228, 2963, 1622, 1604, 1488, 1416, 1283, 1245, 1219, 1156, 1026, 842, 744, 635. HRMS (ESI-TOF): m/z = 481.1599 as $[Pd(H_{-1}o\text{-pda})(^tBu_2bpy)]^+$ (calcd 481.1593). UV-vis (acetone, 293 K, 336 µM) : λ_{max} (nm) (ε (M^{-1} cm^{-1})) = 495.5 (3.02 × 10^3). Anal. Calcd for $C_{25}H_{31}F_3N_4O_3PdS$ {$[Pd(H_{-1}o\text{-pda})(^tBu_2bpy)](OTf)$}: C 47.59, H 4.95, N 8.88; found: C 47.42, H 5.07, N 8.80.

Crystal data for $[Pd(H_{-1}o\text{-pda})(^tBu_2bpy)](OTf)$: $C_{25}H_{31}F_3N_4O_3PdS$, F_w = 631.00, crystal dimensions 0.109 × 0.033 × 0.022 mm^3, monoclinic, space group $P2_1/n$, a = 6.74739(11), b = 18.7128(3), c = 20.9720(4) Å, β = 96.2758(16)°, V = 2632.11(8) Å^3, Z = 4, ρ_{calcd} = 1.592 g cm^{-3}, μ = 69.20 cm^{-1}, T = 93.15 K, λ(CuKα) = 1.54184 Å, 2θ_{max} = 136.454°, 20677/4789 reflections collected/unique (R_{int} = 0.0539), R_1 = 0.0357 ($I > 2\sigma(I)$), wR_2 = 0.0925 (for all data), GOF = 1.062, largest diff. peak and hole 0.758/–1.589 eÅ^{-3}. CCDC deposit number 2316025.

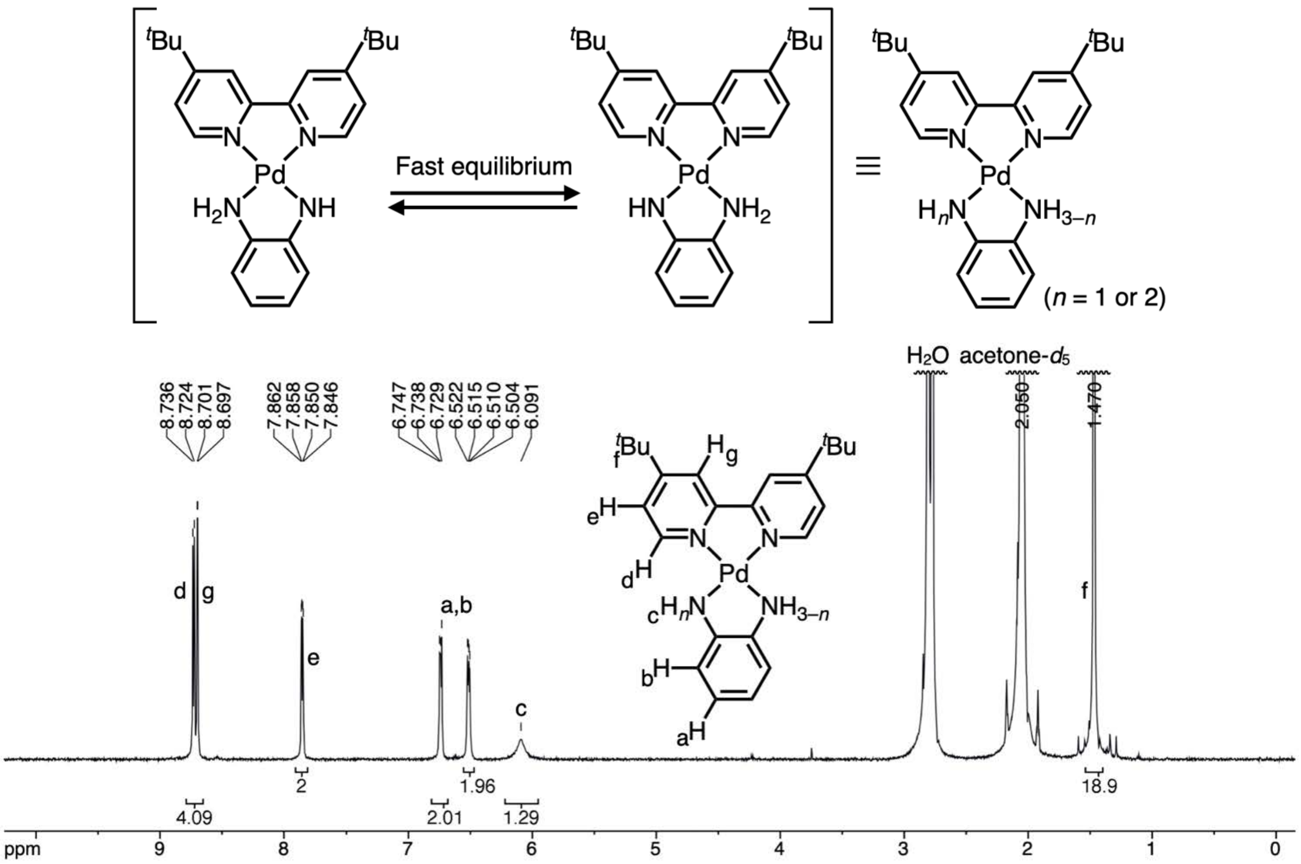

Figure S36. ^{1}H NMR spectrum of H**2**$^+$ (n = 1 or 2) (500 MHz, acetone-d_6, 300 K). Because of the fast equilibrium as shown in the scheme, the NMR signals were observed as the higher symmetric structure and the chemical formula of H**2**$^+$ in NMR analyses are drawn as shown in the upper right.

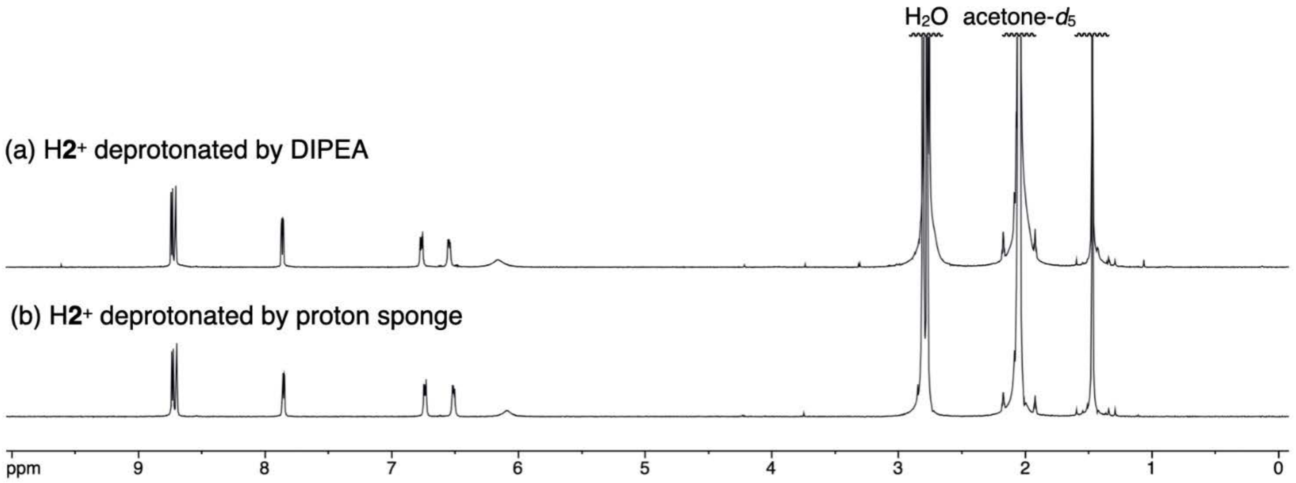

Figure S37. ^{1}H NMR spectra of H**2**$^+$ crystals prepared with (a) DIPEA and (b) proton sponge (500 MHz, acetone-d_6, 300 K).

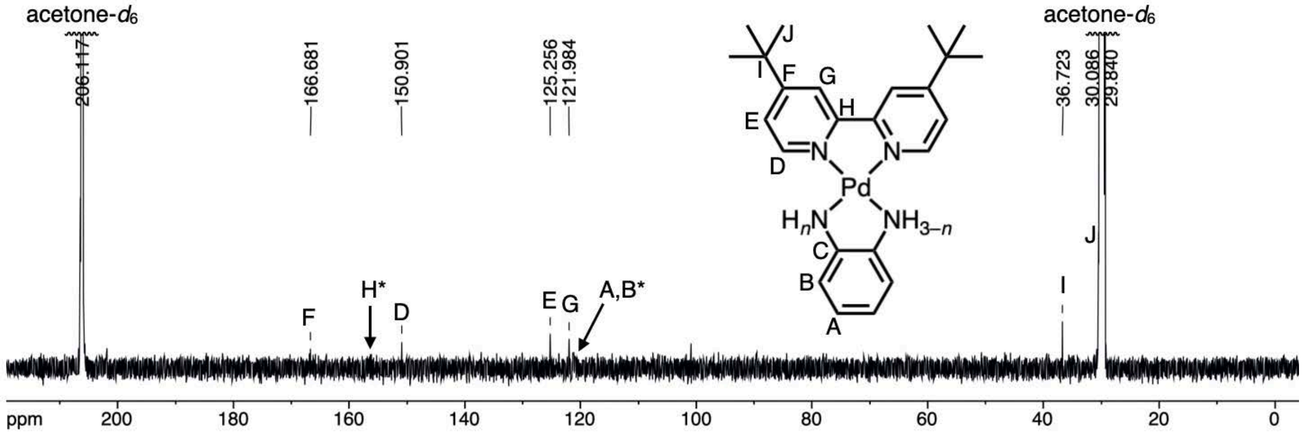

Figure S38. ^{13}C NMR spectrum of H**2**$^+$ (n = 1 or 2) (126 MHz, acetone-d_6, 300 K). Signals with * were deduced using ^{1}H-^{13}C HSQC and HMBC NMR analyses.

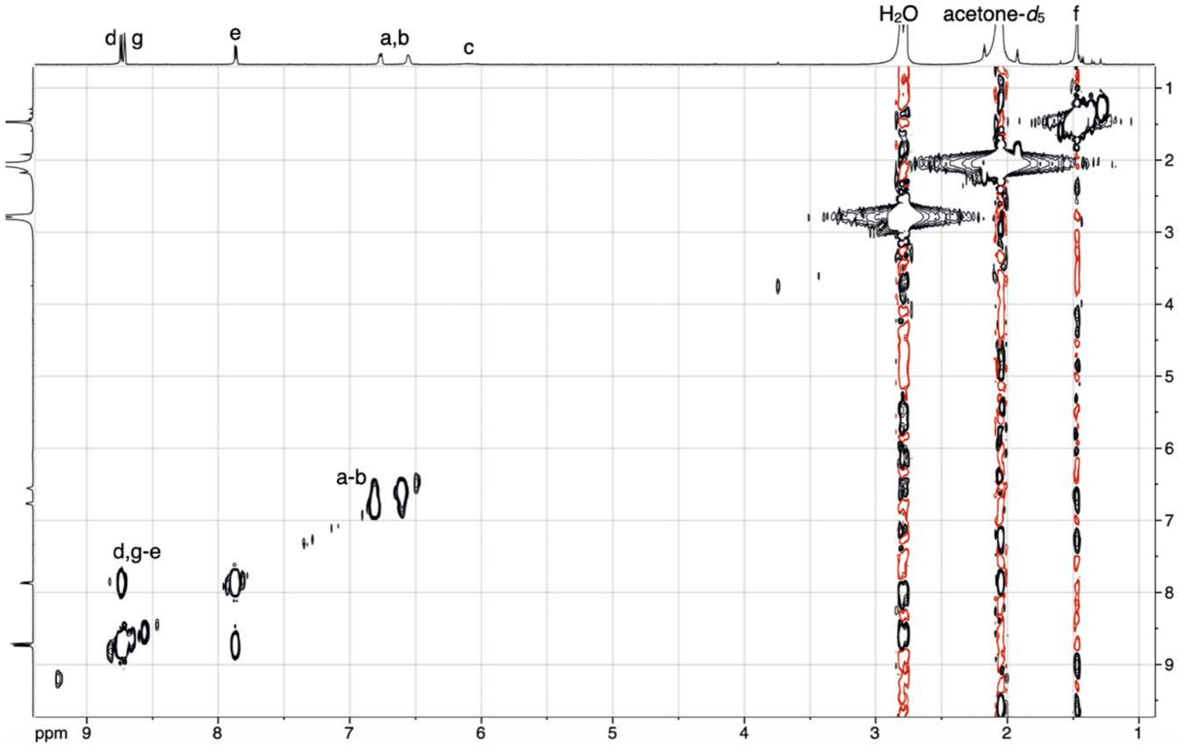

Figure S39. ^{1}H-^{1}H COSY NMR spectrum of H**2**$^+$ (500 MHz, acetone-d_6, 300 K).

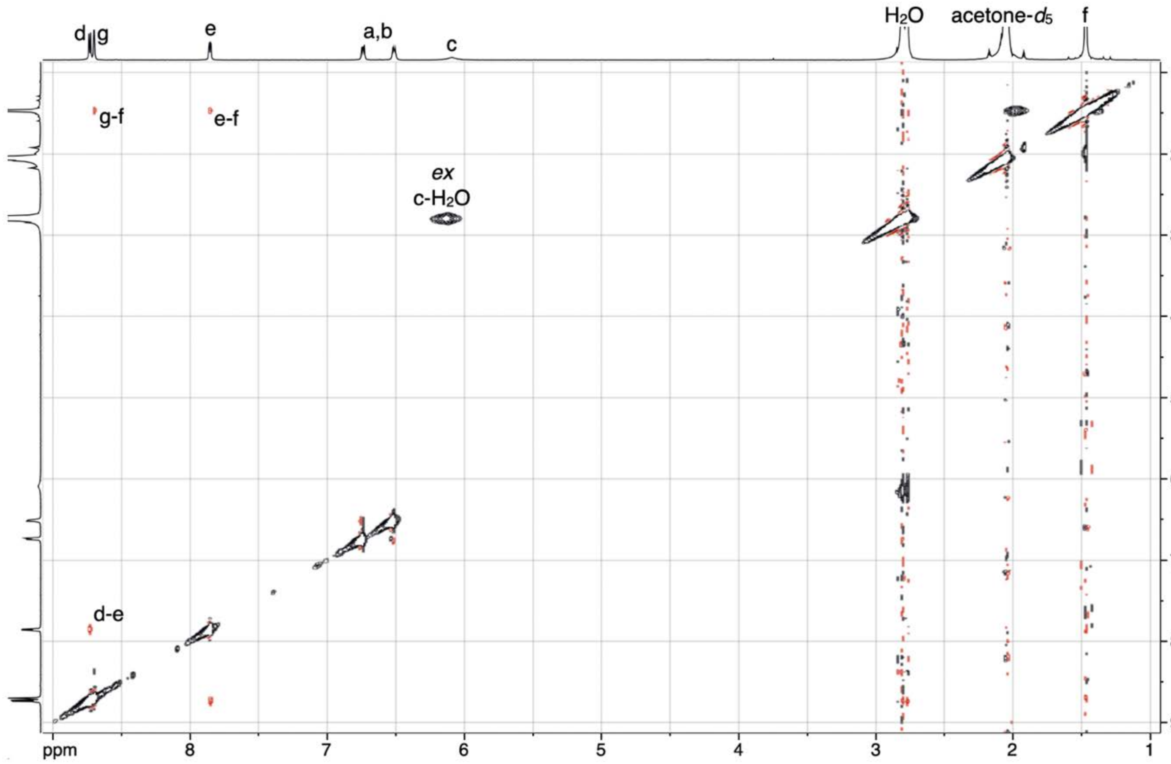

Figure S40. ^1H-^1H NOESY NMR spectrum of H**2**$^+$ (500 MHz, acetone-d_6, 300 K).

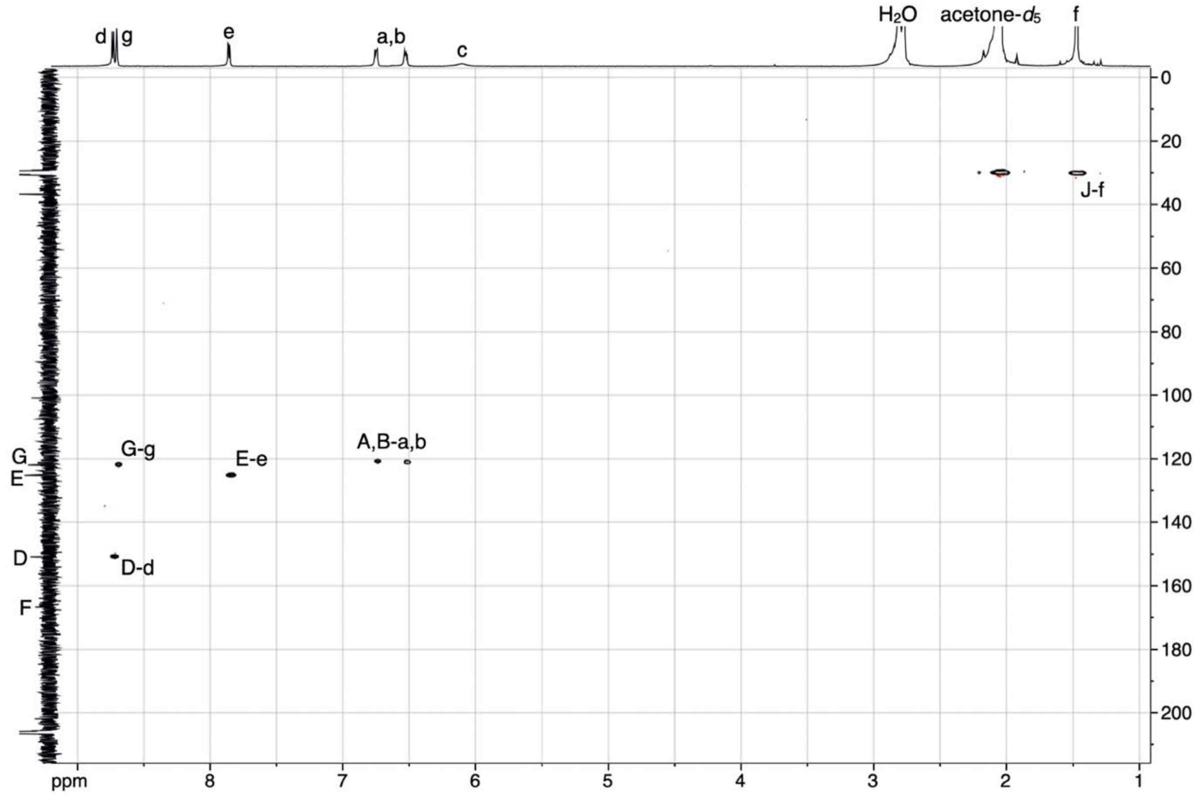

Figure S41. ^1H-^{13}C HSQC NMR spectrum of H**2**$^+$ (500 MHz for ^1H and 126 MHz for ^{13}C, acetone-d_6, 300 K).

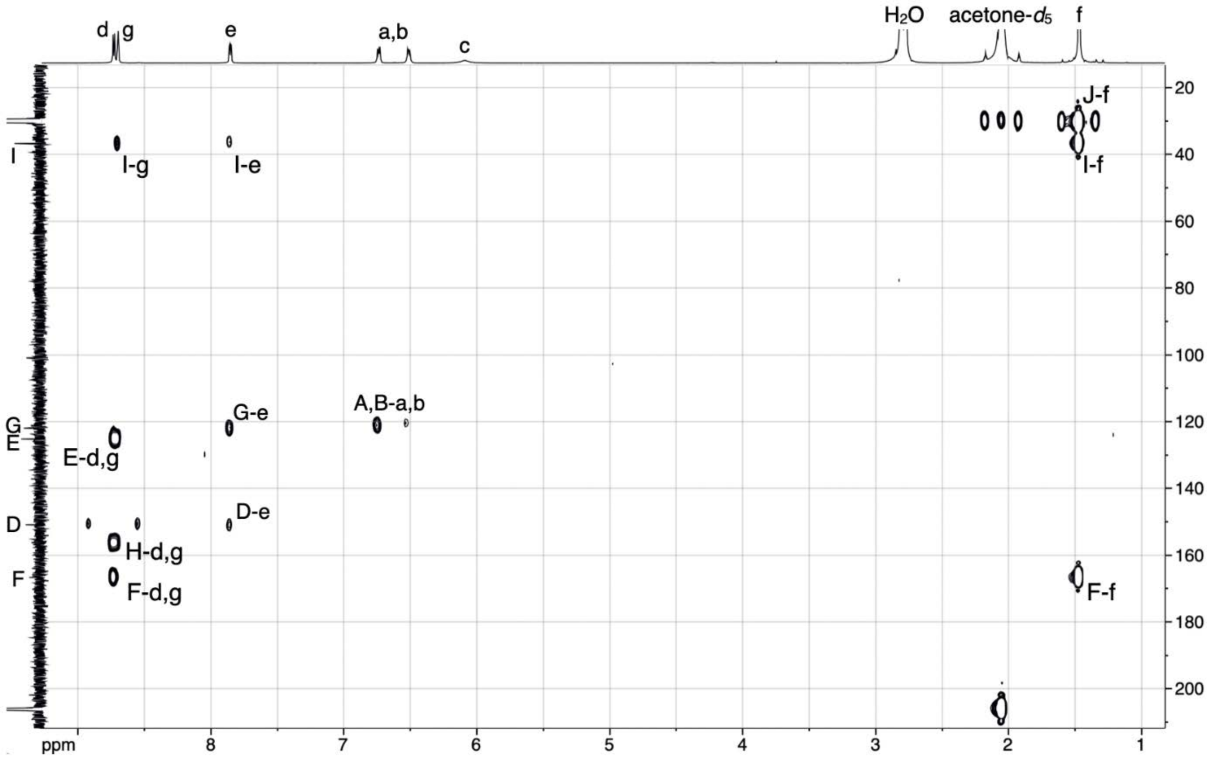

Figure S42. ^{1}H-^{13}C HMBC NMR spectrum of H**2**$^{+}$ (500 MHz for ^{1}H and 126 MHz for ^{13}C, acetone-d_6, 300 K).

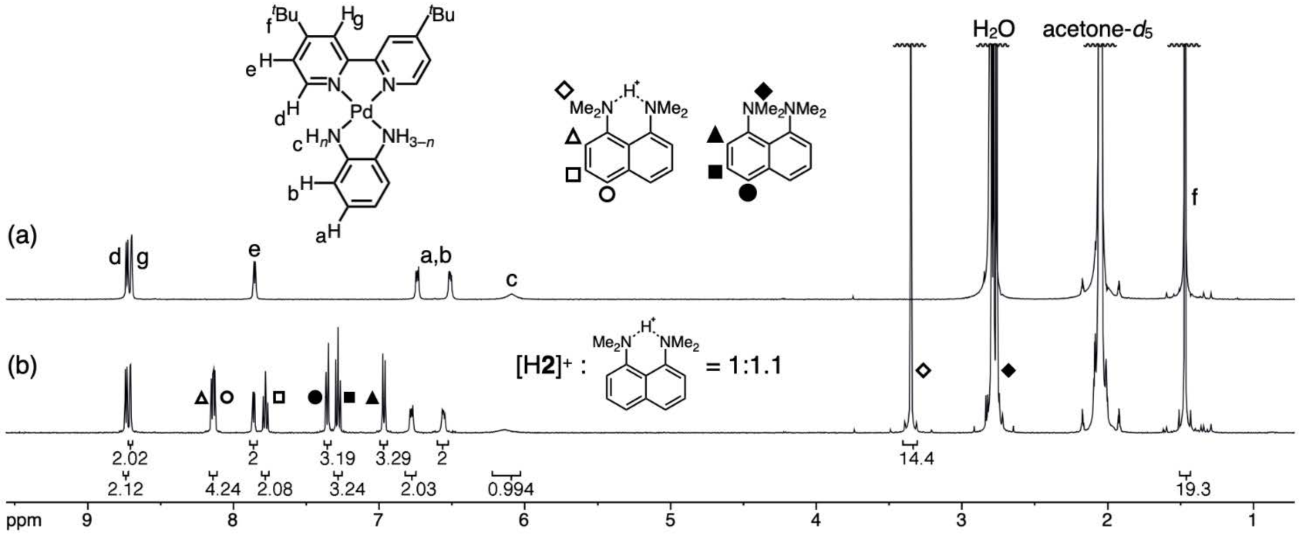

Figure S43. ^{1}H NMR spectra of (a) H**2**$^{+}$ (n = 1 or 2) and (b) a mixture of H_2**2**$^{2+}$ and 2.6 equiv. of proton sponge.

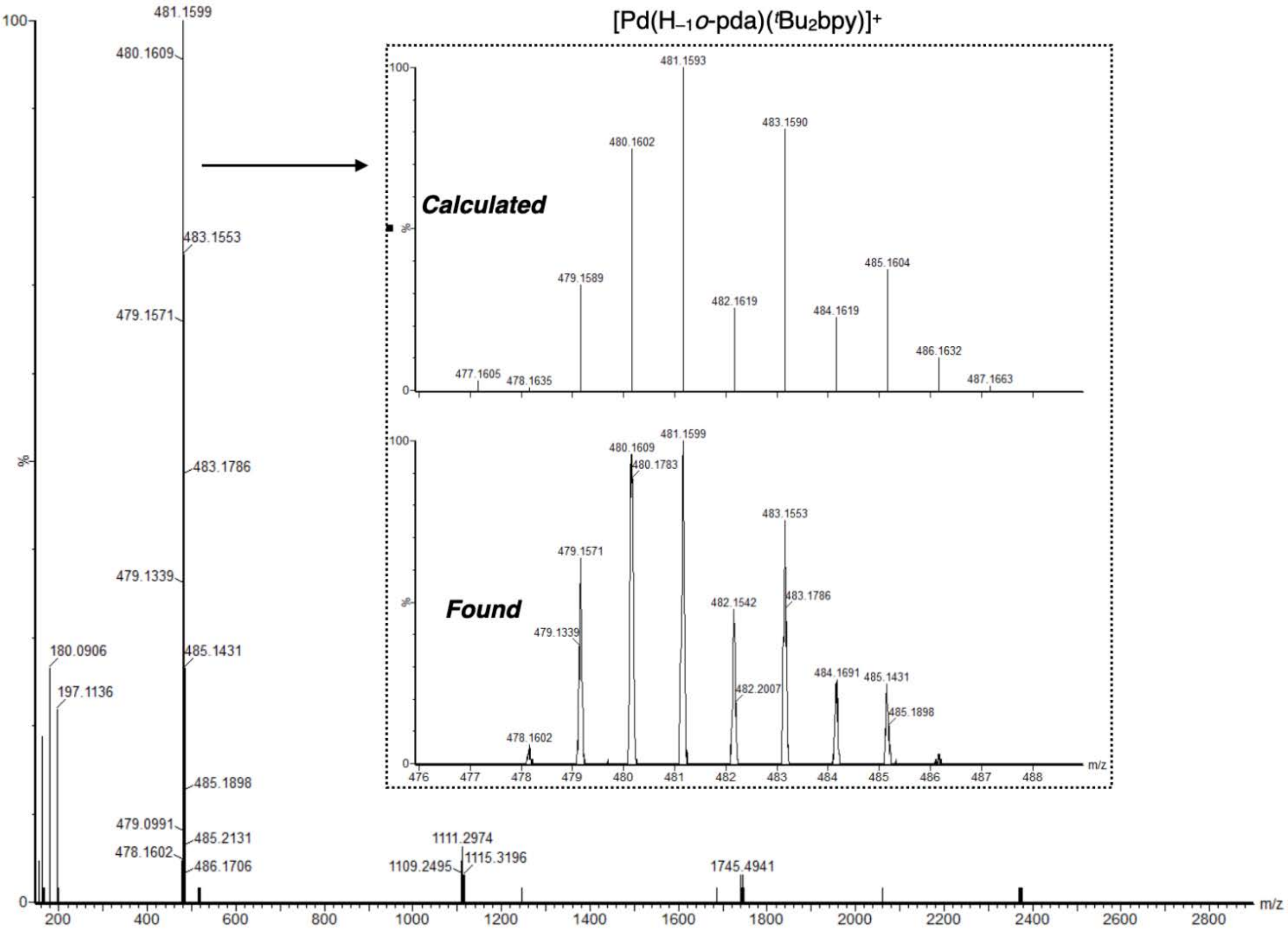

Figure S44. High-resolution ESI-mass spectrum of H**2**$^{+}$ (positive, acetone). The capillary voltage during the high-resolution ESI-mass measurement was decreased to 150 V because H**2**$^{+}$ was easily oxidized and deprotonated during the measurement under normal voltage conditions (3000 V).

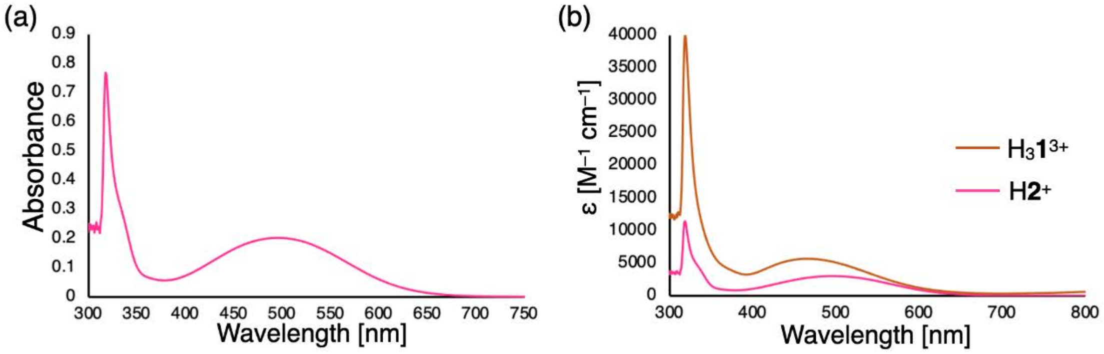

Figure S45. (a) UV-vis spectrum of H**2**$^{+}$ (acetone, 293 K, 336 μM, l = 0.2 cm). (b) UV-vis spectra of (pink) [H$_{-1}$**2**]$^{+}$ (acetone, 293 K, 336 μM, l = 0.2 cm) and (brown) H$_3$**1**$^{3+}$ deprotonated by Na_2CO_3 (acetone, 293 K, 96.3 μM, l = 0.2 cm). The sudden decrease of the absorbance around 320 nm is due to the absorption of acetone.

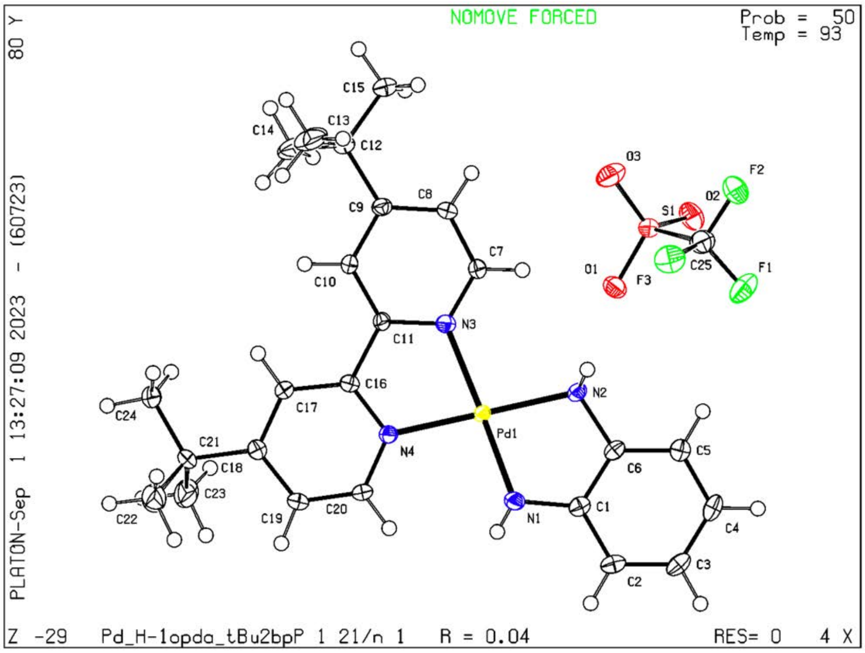

Figure S46. ORTEP drawing of H**2**·OTf at the 50% probability level. Color: C black, N blue, O red, F yellow-green, S dark red, and Pd yellow. CCDC deposit number of H**2**·OTf is 2316025. This figure was produced by the checkCIF report of the International Union of Crystallography.

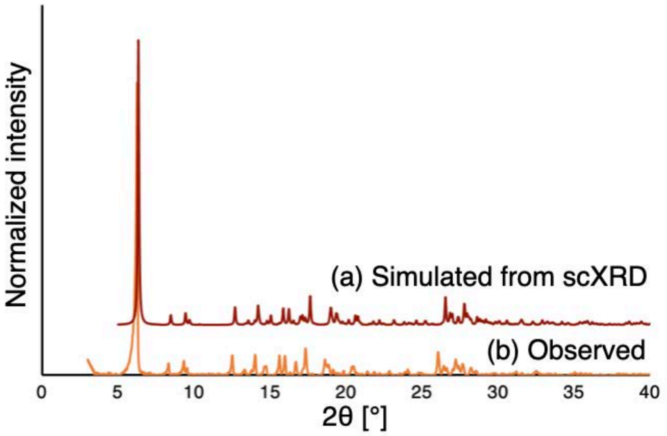

Figure S47. (a) pXRD pattern simulated from single-crystal XRD (scXRD) measured at –180 °C and (b) observed pXRD pattern of H**2**·OTf measured at room temperature. H**2**·OTf for scXRD analysis was crystallized from the THF solution of H_2**2**·2OTf and DIPEA, and H**2**·OTf for pXRD measurement was crystallized from the acetone solution of H_2**2**·2OTf and proton sponge by vapor diffusion of Et_2O.

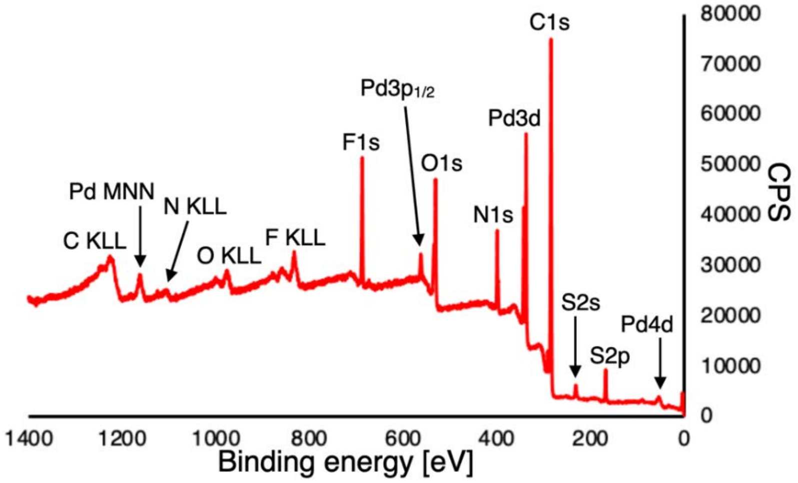

Figure S48. XPS spectrum (survey scan) of H**2**·OTf.

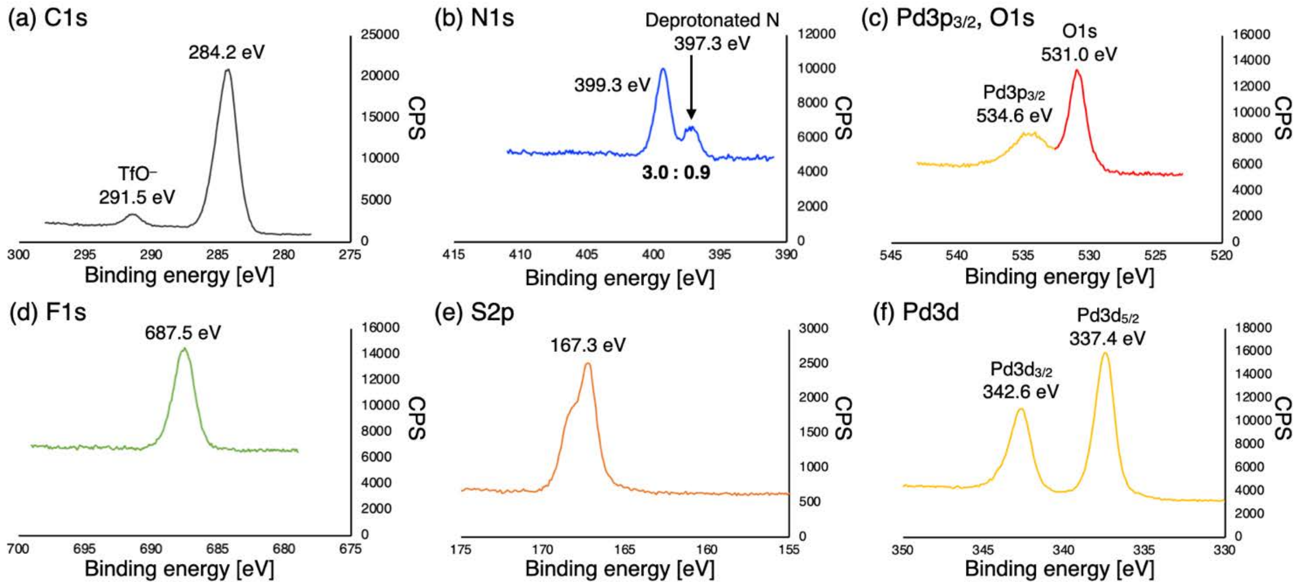

Figure S49. XPS spectra of (a) C1s, (b) N1s, (c) Pd3p$_{3/2}$ and O1s, (d) F1s, (e) S2p and (f) Pd3d of H**2**·OTf. H**2**·OTf for XPS measurement was prepared by the crystallization from the THF solution of **2**·2OTf and DIPEA.

	C1s	N1s	O1s	F1s	S2p	Pd3d
Observed ratio	25.9	3.9	3.3*	2.9	1.0	1.0
Theoretical chemical formula	25	4	3	3	1	1

Figure S50. Table of the ratio of each element measured by XPS of H**2**·OTf. *: The ratio of O1s was analyzed by deconvolution because of the overlapping with the Pd3p$_{3/2}$ signal.

3.3 Titration of DIPEA to $H_2$2·2OTf in acetone and estimation of the relative acid dissociation constant of $H_2 2^{2+}$

3.3.1 Theoretical analysis of the relative acid dissociation constant of $H_2 2^{2+}$

$$H_2\mathbf{2}^{2+} + \mathrm{DIPEA} \underset{}{\overset{K}{\rightleftharpoons}} H\mathbf{2}^{+} + \mathrm{DIPEA{\cdot}H^{+}}$$

We consider the deprotonation reaction of $H_2\mathbf{2}^{2+}$ upon the addition of DIPEA as a base to estimate the equilibrium constant. In this model, the equilibrium constant *K* is given below:

$$K = \frac{[\mathrm{H}\mathbf{2}^{+}][\mathrm{BH}^{+}]}{[\mathrm{H}_2\mathbf{2}^{2+}][\mathrm{B}]} \qquad \cdots(3\text{-}1)$$

where $[\mathrm{H}_2\mathbf{2}^{2+}]$ and $[\mathrm{H}\mathbf{2}^{+}]$ are the concentration of $H_2\mathbf{2}^{2+}$ and $H\mathbf{2}^{+}$, respectively, and [B] and $[\mathrm{BH}^{+}]$ are the concentration of a base and its protonated base, DIPEA and [DIPEA·H^{+}], respectively. During the titration experiment, the total amount of Pd complexes was constant to the initial concentration, C_0, and the total amount of the added DIPEA ($[\mathrm{B}_{tot}]$) was the sum of [B] and $[\mathrm{BH}^{+}]$, which leads to the equation (3-2) and (3-3).

$$C_0 = [\mathrm{H}_2\mathbf{2}^{2+}] + [\mathrm{H}\mathbf{2}^{+}] \qquad \cdots(3\text{-}2)$$

$$[\mathrm{B}_{tot}] = [\mathrm{B}] + [\mathrm{BH}^{+}] \qquad \cdots(3\text{-}3)$$

From the charge balance, the equation (3-4) is given below:

$$[\mathrm{BH}^{+}] = [\mathrm{H}\mathbf{2}^{+}] \qquad \cdots(3\text{-}4)$$

Substitution of equations (3-2) to (3-4) into equation (3-1) gives a quadratic equation in [H$\mathbf{2}^{+}$] (3-5).

$$(K-1)[\mathrm{H}\mathbf{2}^{+}]^2 - K(C_0 + [\mathrm{B}_{tot}])[\mathrm{H}\mathbf{2}]^{+}] + KC_0[\mathrm{B}_{tot}] = 0 \qquad \cdots(3\text{-}5)$$

Solving this quadratic equation gives [H$\mathbf{2}^{+}$] below.

$$[\mathrm{H}\mathbf{2}^{+}] = \frac{K(C_0+[\mathrm{B}_{tot}]) - \sqrt{K^2(C_0+[\mathrm{B}_{tot}])^2 - 4(K-1)KC_0[\mathrm{B}_{tot}]}}{2(K-1)} \qquad \cdots(3\text{-}6)$$

Here, the other solution of [H$\mathbf{2}^{+}$] is ignored because [H$\mathbf{2}^{+}$] must be lower than C_0. The equations (3-2) and (3-6) are substituted into the equation (3-7), and the *K* value of H$\mathbf{2}^{+}$ was estimated by curve fitting analysis of titration UV-vis spectroscopy based on the equation below (3-7). The error of the fitting analysis was calculated as a standard error.

$$Abs. = \varepsilon'\ l[\mathrm{H}_2\mathbf{2}^{2+}] + \varepsilon l[\mathrm{H}\mathbf{2}^{+}] \qquad \cdots(3\text{-}7)$$

3.3.2 Titration of DIPEA to $H_2 2^{2+}$ in acetone

DIPEA was titrated to an acetone solution of H_2**2**·2OTf to estimate the relative acid dissociation constant (*K*) of $H_2\mathbf{2}^{2+}$ to DIPEA.

H_2**2**·2OTf (1.01 mg, 1.29 μmol) was dissolved in acetone (5 mL, 259 μM). 4 mL of this acetone solution was transferred to a quartz UV-vis cell and UV-vis spectroscopy of this solution was performed. To this solution was added an acetone solution of DIPEA (6.34 mg, 49.1 mM), and UV-

vis spectroscopy was performed. The addition of DIPEA and measurement of UV-vis spectra were repeated until the absorption around 500 nm was saturated.

After plotting the absorbance at 496 nm versus the equivalents of DIPEA to $H_2\mathbf{2}^{2+}$, curve fitting analysis was performed based on equation (3-7) where the absorption coefficients of $H_2\mathbf{2}^{2+}$ and $H\mathbf{2}^{+}$ at 496 nm were substituted for ε' and ε, respectively.

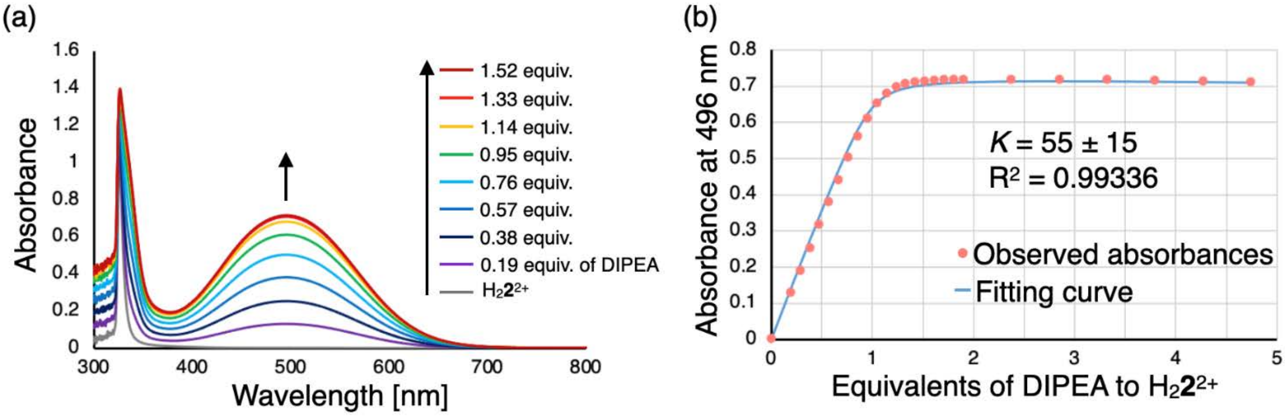

Figure S51. (a) UV-vis spectra of $H_2\mathbf{2}^{2+}$ upon titration of DIPEA (acetone, 293 K, l = 1.0 cm, 259 μM). (b) Plot of the absorbance of the model Pd complex at 496 nm versus equivalents of DIPEA to $H_2\mathbf{2}^{2+}$: (pink points) measured absorbances and (light blue line) fitting curve based on the equation (3-7).

4. Estimation of the rate of helicity inversion by EXSY NMR

4.1 Theoretical analysis of the rate of helicity inversion based on ^{1}H-^{1}H EXSY NMR measurements[5]

^{1}H-^{1}H EXSY NMR measurement was conducted with a Bruker AVANCE500 (500 MHz) spectrometer at 300 K using the standard pulse sequence for phase-sensitive NOESY measurements programmed in XWinNMR Bruker software. Relaxation decay (D1) was set to 2 s, and appropriate mixing times (τ_m = 50–1000 ms) for each trinuclear metallocycle were chosen.

When populations of both exchangeable (*P*/*M*)-isomers were equal, rate constants of exchange processes between two spectroscopically-distinct states (k) are derived according to the equations:

$$k = \frac{1}{\tau_m} \ln \frac{r+1}{r-1}$$

$$r = \frac{I_{AA}+I_{BB}}{I_{AB}+I_{BA}}$$

where τ_m is the mixing time of NOESY measurement, I_{AA} and I_{BB} are the integral values of diagonal peaks, and I_{AB} and I_{BA} are those of cross peaks. After phase and baseline were corrected in both dimensions, the integral values of the diagonal and cross peaks were calculated using iNMR software. Using these calculated integral values, the rate constant (k) was calculated from the slope of the plots

of ln[$(r + 1)/(r - 1)$] versus mixing time (τ_m) by linear regression analysis. In these measurements, the rate constant of helicity inversion (k) was defined as the sum of the rate constants of helicity inversion from (M)- to (P)-isomers (k_{MP}) and from (P)- to (M)-isomers (k_{PM}). Linear regression analysis and estimation of the standard error were conducted by StatPlus software.[6]

In the EXSY analyses of trinuclear complexes described below, the helicity inversion rate can be estimated using combinations of chemical exchange signals, such as H_d and H_f, H_o and H_v, and H_p and H_u. Some of these exchange signal combinations overlapped with other signals and were therefore unsuitable for estimating inversion rates. In such cases, the estimated inversion rates were similar for both combinations of exchange signals, so other non-overlapping combinations of exchange signals were used. The values shown in the EXSY spectra were the integrals of the diagonal and exchange signals calculated by the iNMR software.

4.2 Theoretical analysis of activation parameters of helicity inversion based on VT EXSY NMR measurements

The enthalpy of activation ($\Delta H^{\ddagger}$) and the entropy of activation ($\Delta S^{\ddagger}$) were derived according to the Eyring equation;

$$\ln\frac{k}{T} = -\frac{\Delta H^{\ddagger}}{R}\frac{1}{T} + \ln\frac{k_B}{h} + \ln\frac{\Delta S^{\ddagger}}{R}$$

where R is the gas constant, k_B is the Boltzmann constant, and h is the Planck constant. Using these two activation parameters, the Gibbs energy of activation ($\Delta G^{\ddagger}$) was calculated according to the equation:

$$\Delta G^{\ddagger} = \Delta H^{\ddagger} - T\Delta S^{\ddagger}$$

The rate constants (k) of helicity inversion at variable temperatures were estimated by VT EXSY measurements. Based on the plots of ln(k/T) versus $1/T$ (Eyring plot), $\Delta H^{\ddagger}$ and $\Delta S^{\ddagger}$ were estimated from the slope and the y-intercept of the plot, respectively.

4.3 VT EXSY NMR measurements of $H_3\mathbf{1}^{3+}$ in acetone-d_6 and estimation of the rate and activation parameters of the helicity inversion

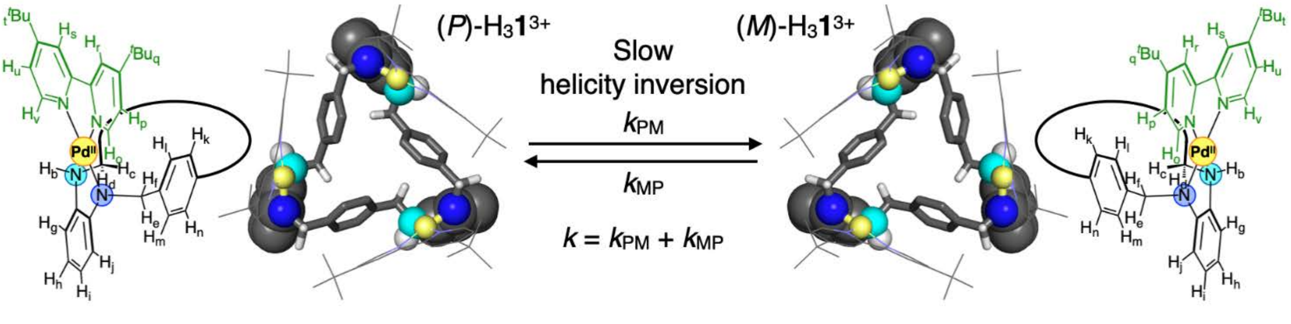

Figure S52. Helicity inversion between (P)- and (M)-$H_3\mathbf{1}^{3+}$ with their chemical structural formula.

H_6**1**·6OTf (1.85 mg, 0.68 μmol) was dissolved in acetone-d_6 (2.5 mL, 0.27 mM). Then, 0.5 mL of this solution was transferred to a vial and solid Na_2CO_3 (0.36 mg, 3.4 μmol, 25 equiv.) was added. This reaction suspension was sonicated at room temperature for 5 min and was centrifuged to remove precipitate. The supernatant was transferred to an NMR tube, and 2D ^{1}H-^{1}H EXSY NMR measurements of this red-orange solution of $[H_{-3}\mathbf{1}]^{3+}$ (0.27 mM) were conducted at 310 K.

In the same manner, solid Na_2CO_3 (0.35 mg, 3.3 μmol, 24 equiv. and 0.37 mg, 3.5 μmol, 26 equiv.) was reacted with the acetone-d_6 solutions (0.5 mL) of H_6**1**·6OTf prepared above and EXSY of the deprotonated trinuclear Pd^{II} complex $H_3\mathbf{1}^{3+}$ was conducted at 300 K and 290 K, respectively.

To estimate the rate constant of helicity inversion of $H_3\mathbf{1}^{3+}$, three sets of chemical exchange signals between H_d and H_f, between H_o and H_v, and between H_p and H_u can be used. However, since the ^{1}H NMR signals of H_v and H_u overlapped with other proton signals at 290 K, the signals between H_d and H_f were used to estimate the rate constant (k) and activation parameters. Because $H_3\mathbf{1}^{3+}$ decomposes slowly at high temperatures and the inversion is too slow at low temperatures to observe the exchange signals, we estimated the activation parameters of $H_3\mathbf{1}^{3+}$ using Eyring plots based on the EXSY measurements at 290, 300, and 310 K. As we described in the preparation of $H_3\mathbf{1}^{3+}$ using Na_2CO_3, 3 equiv. of NaOTf remained in the acetone-d_6 solution of $H_3\mathbf{1}^{3+}$.

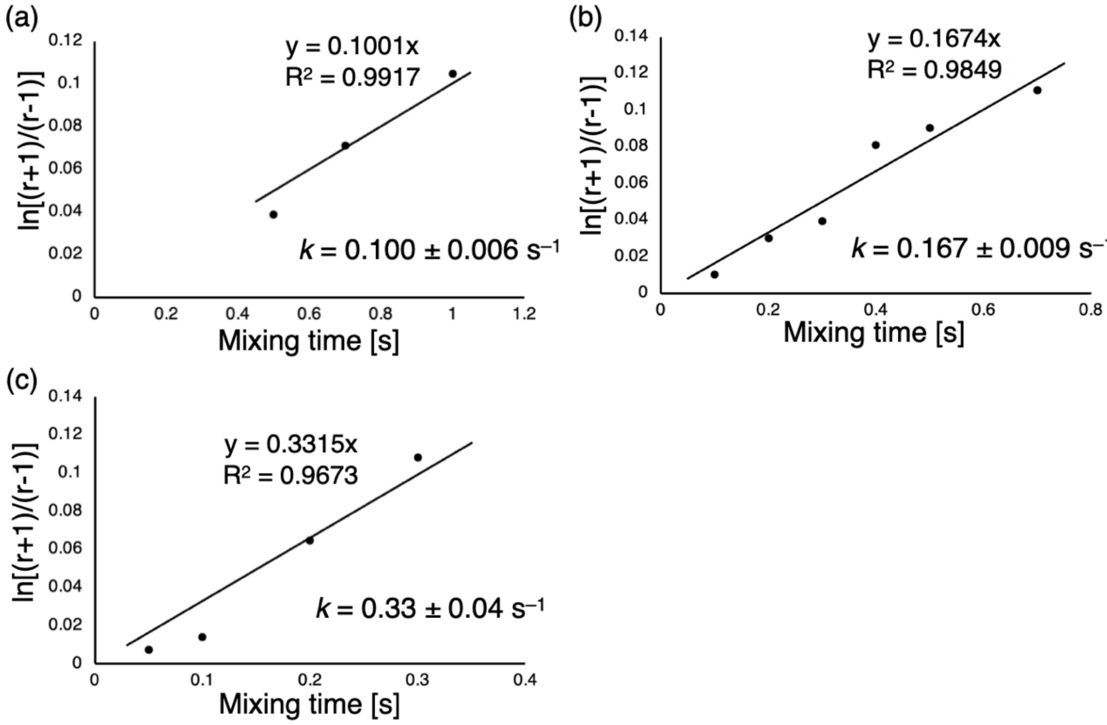

Figure S53. Plots of $\ln[(r + 1)/(r - 1)]$ versus mixing time τ_m for $H_3\mathbf{1}^{3+}$ in acetone-d_6 from ^{1}H-^{1}H VT EXSY spectra (500 MHz, acetone-d_6, 0.27 mM) recorded with different mixing times (0.05–1.0 s) and their fitting line with a formula based on chemical exchange signals between H_d and H_f; (a) at 290 K, (b) 300 K, and (c) 310 K.

(a)

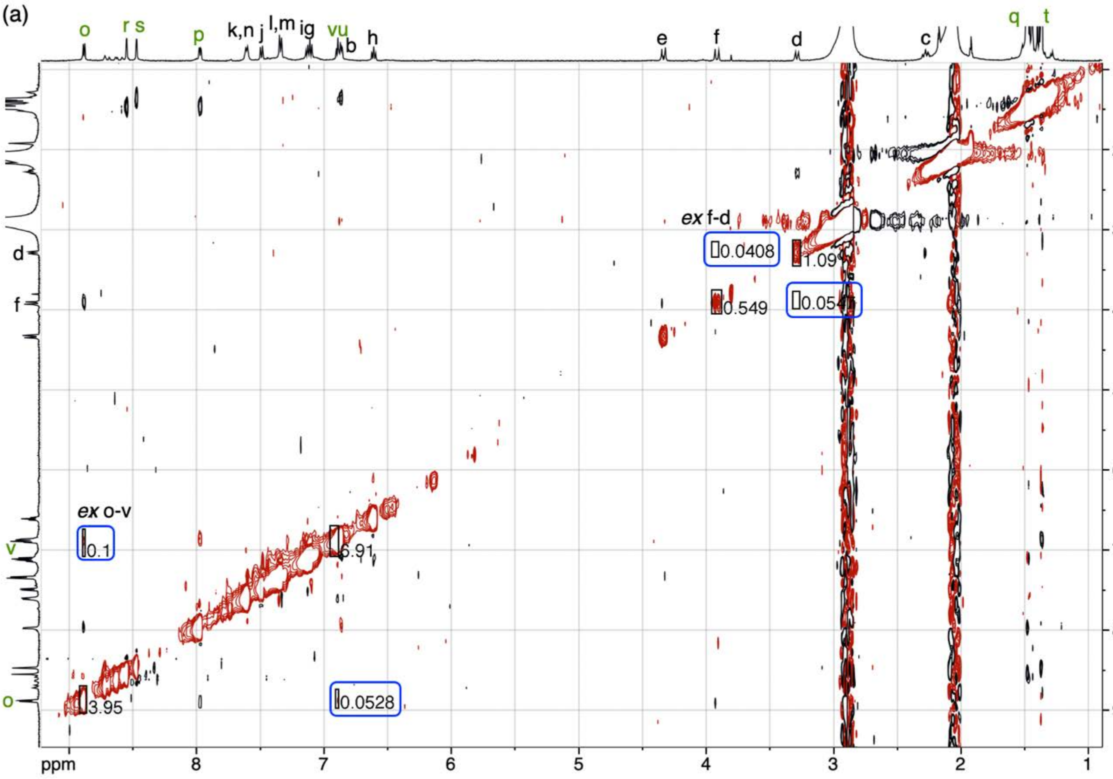
o r s p k,n j l,m ig vu b h e f d c q t
ex f-d
0.0408
1.09
0.549
0.054
ex o-v
0.1
6.91
3.95
0.0528
ppm

(b)

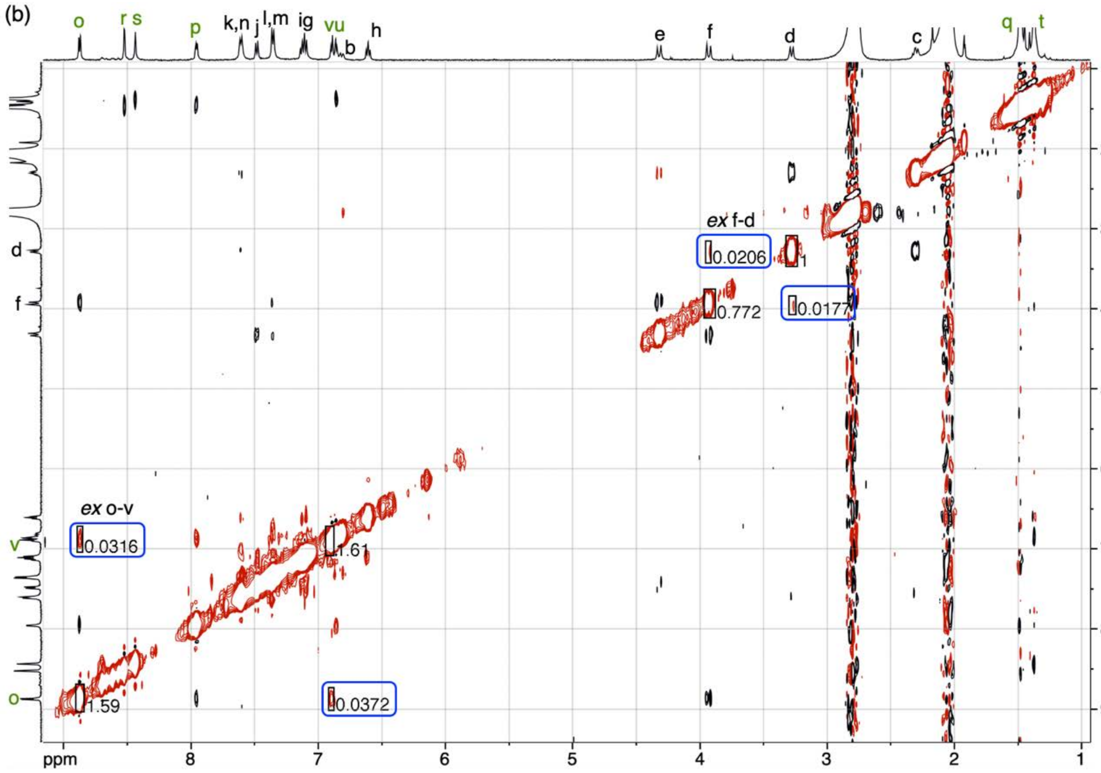
o r s p k,n j l,m ig vu b h e f d c q t
ex f-d
0.0206
1
0.772
0.0177
ex o-v
0.0316
1.61
1.59
0.0372
ppm

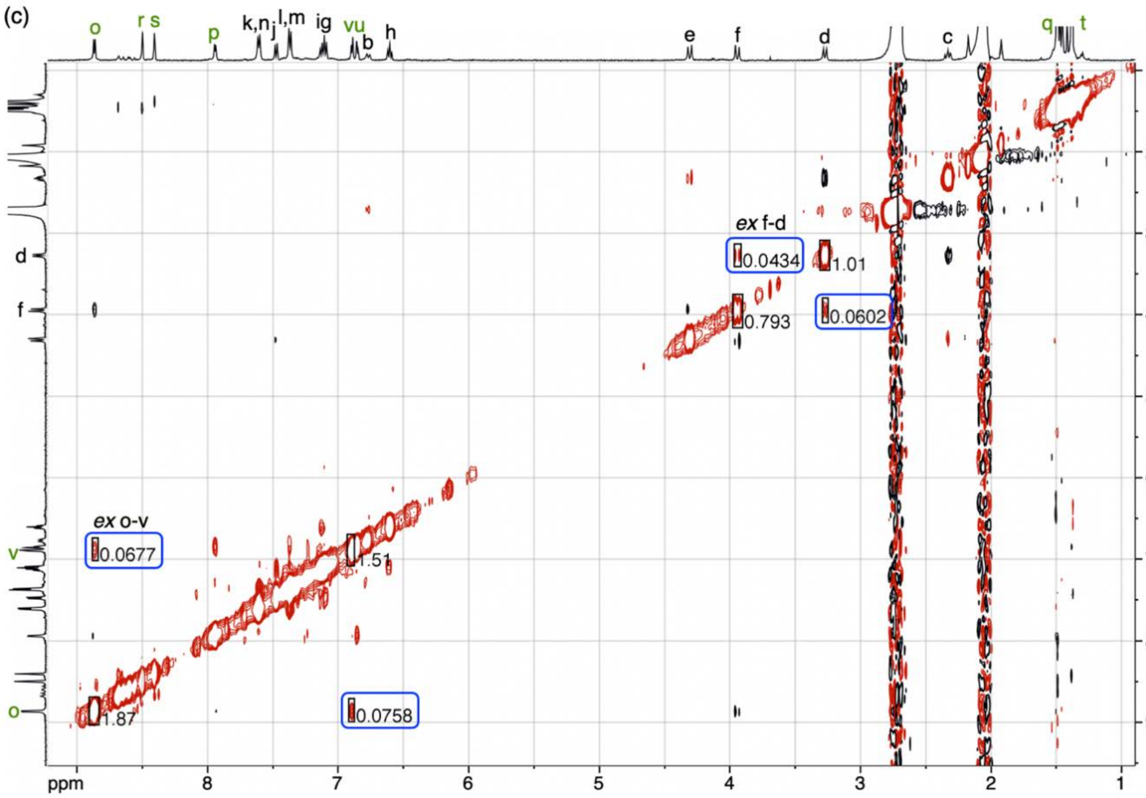

Figure S54. VT ^{1}H-^{1}H EXSY NMR spectra of $H_3\mathbf{1}^{3+}$ (500 MHz, acetone-d_6, 0.27 mM). Blue squares indicate the chemical exchange signals; (a) measured at 290 K and mixing time τ_m = 1.0 s, (b) 300 K and τ_m = 0.3 s, and (c) 310 K and τ_m = 0.3 s.

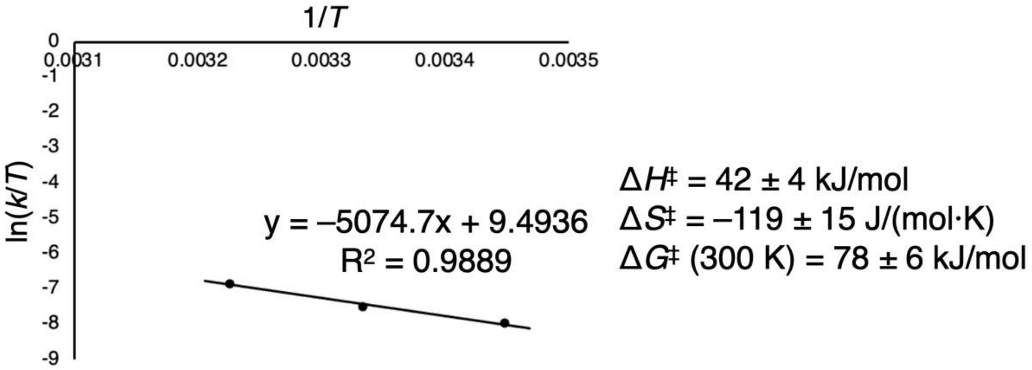

Figure S55. Eyring plot for $H_3\mathbf{1}^{3+}$ in acetone-d_6 and the enthalpy ($\Delta H^{‡}$), entropy ($\Delta S^{‡}$), and Gibbs energy of activation at 300 K ($\Delta G^{‡}$) calculated from the plot.

4.4 Estimation of the helicity inversion rate of $H_3\mathbf{1}^{3+}$ in distilled acetone-d_6

The distilled acetone-d_6 was prepared by following the procedure below. Activated molecular sieves 4A was added to undried acetone-d_6. This acetone-d_6 was stood at room temperature under an Ar atmosphere for 4.5 h and then distilled under an Ar atmosphere.

To H_6**1**·6OTf (0.36 mg, 0.13 µmol, 1.0 equiv.) and Na_2CO_3 (0.32 mg, 3.0 µmol, 23 equiv.), the distilled acetone-d_6 (450 µL, 0.29 mM) was added. This reaction suspension was sonicated at room temperature for 5 min. After the precipitate was removed by centrifugation, the supernatant was transferred to an NMR tube, and 2D ^{1}H-^{1}H EXSY NMR measurements of the red-orange solution were conducted at 300 K. The amount of water was estimated by comparing the integral ratio of water to H_3**1**$^{3+}$.

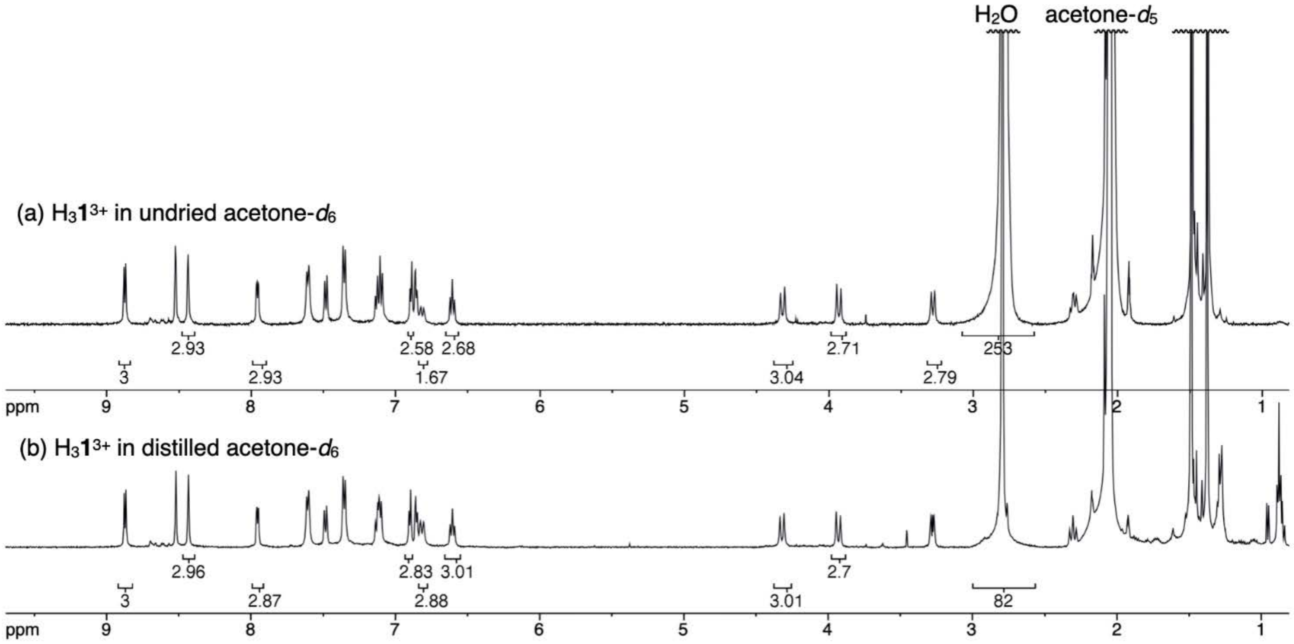

Figure S56. ^{1}H NMR spectra of H_3**1**$^{3+}$ in (a) undried acetone-d_6 (0.27 mM) and (b) distilled acetone-d_6 (0.29 mM) (500 MHz, 300 K).

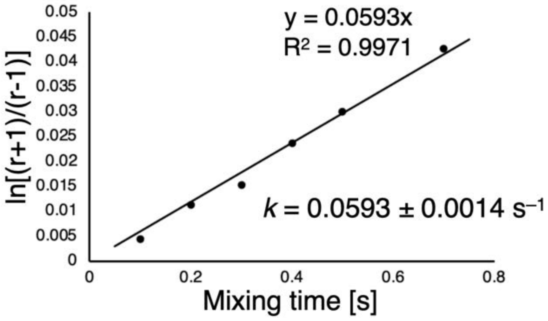

Figure S57. Plots of ln[$(r + 1)/(r – 1)$] versus mixing time τ_m for H_3**1**$^{3+}$ from ^{1}H-^{1}H VT EXSY spectra (500 MHz, distilled acetone-d_6, 300 K, 0.29 mM) recorded with different mixing times (0.1–0.7 s) and their fitting line with a formula based on chemical exchange signals between H_o and H_v.

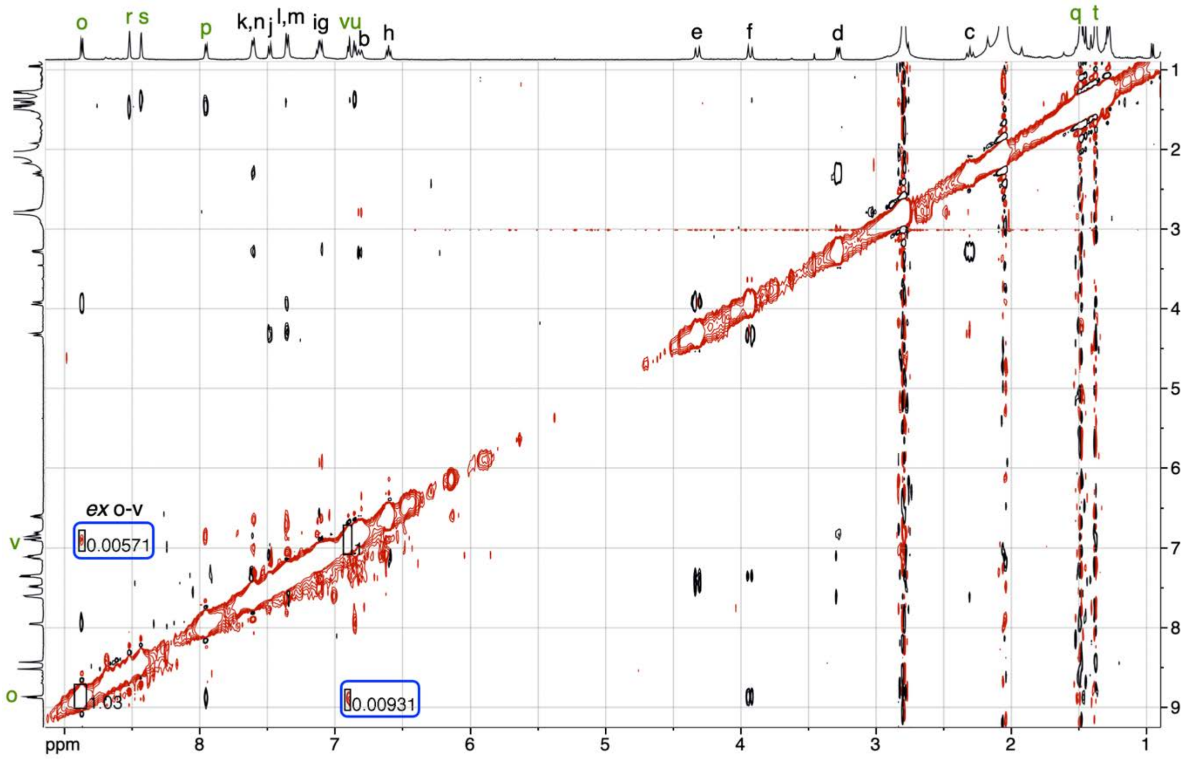

Figure S58. ^1H-^1H EXSY NMR spectra of $H_3\mathbf{1}^{3+}$ (500 MHz, distilled acetone-d_6, 300 K, mixing time τ_m = 0.3 s, 0.29 mM). Blue squares indicate the chemical exchange signals.

4.5 Kinetic isotope effect on the helicity inversion of $H_3\mathbf{1}^{3+}$ with water

To an undried acetone-d_6 solution (0.6 mL) of $H_6\mathbf{1}\cdot 6OTf$ (0.44 mg, 0.16 μmol, 1.0 equiv.) was added Na_2CO_3 (0.71 mg, 6.7 μmol, 41 equiv.). This suspension was sonicated at room temperature for 5 min and centrifuged to remove precipitate. The supernatant (500 μL) was transferred to an NMR tube and H_2O (5 μL) was added. 2D ^1H-^1H EXSY NMR spectroscopy of this acetone-d_6:H_2O = 100:1 solution of $H_3\mathbf{1}^{3+}$ (505 μL, 0.27 mM) was conducted.

In the same manner, an acetone-d_6:D_2O = 100:1 solution of $H_3\mathbf{1}^{3+}$ (505 μL, 0.27 mM) was also prepared, and 2D ^1H-^1H EXSY spectroscopy was conducted.

To estimate the rate constant of helicity inversion of $H_3\mathbf{1}^{3+}$, three sets of chemical exchange signals between H_d and H_f, between H_o and H_v, and between H_p and H_u can be used. However, since the ^1H NMR signals of H_v and H_d overlapped with other signals, the signals between H_p and H_u were used to estimate the rate constant (k). Then, the rate constants in acetone-d_6:H_2O = 100:1 (k_H) and in acetone-d_6:D_2O = 100:1 (k_D) were compared to evaluate the kinetic isotope effect on the helicity inversion of $H_3\mathbf{1}^{3+}$. As we described in the preparation of $H_3\mathbf{1}^{3+}$, 3 equiv. of NaOTf remained in the acetone-d_6 solution of $H_3\mathbf{1}^{3+}$.

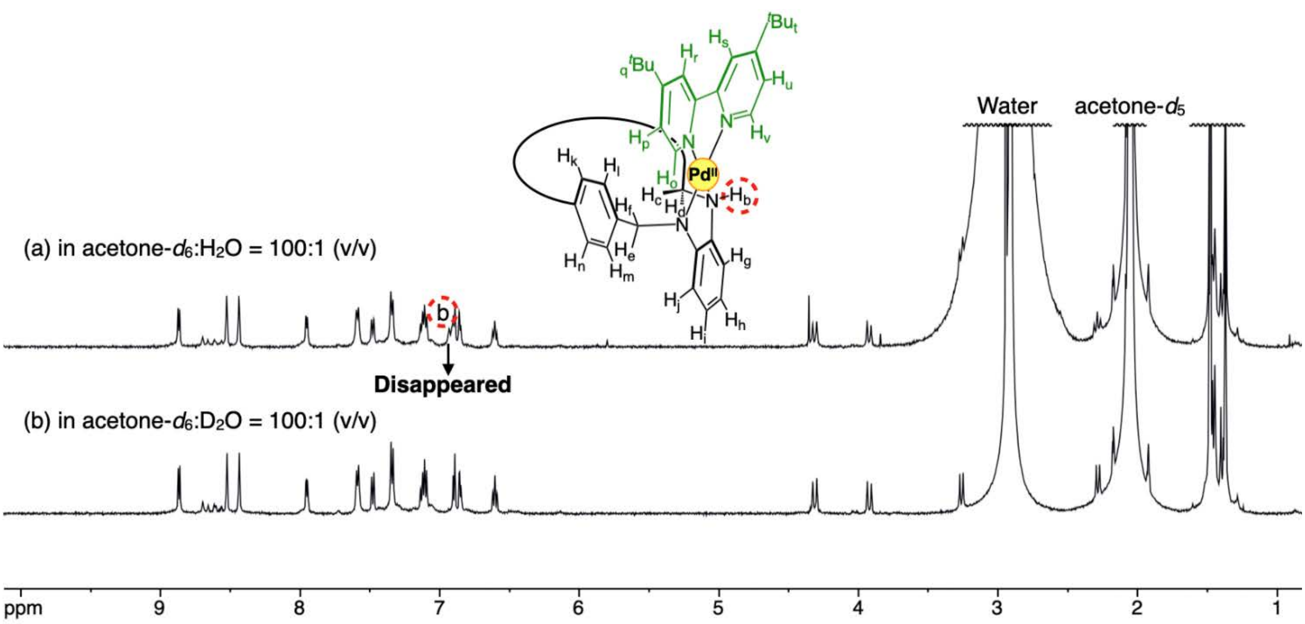

Figure S59. ^{1}H NMR spectra of $H_3\mathbf{1}^{3+}$ (a) in acetone-d_6:H_2O = 100:1 (v/v) and (b) in acetone-d_6:D_2O = 100:1 (v/v) (500 MHz, 300 K).

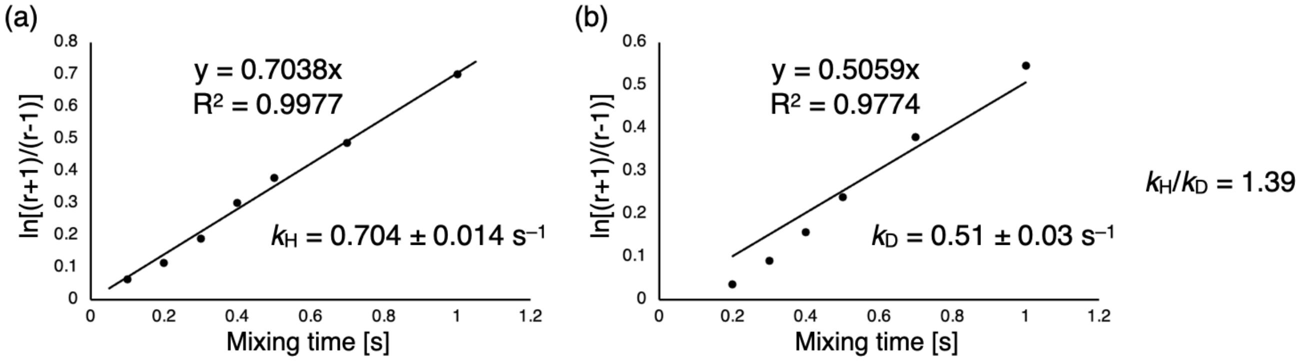

Figure S60. Plots of ln[(r + 1)/(r – 1)] versus mixing time τ_m for $H_3\mathbf{1}^{3+}$ from ^{1}H-^{1}H VT EXSY spectra (500 MHz, 300 K, 0.27 mM) recorded with different mixing times (0.1–1.0 s) and their fitting line with a formula based on chemical exchange signals between H_p and H_u; (a) in acetone-d_6:H_2O = 100:1 and (b) in acetone-d_6:D_2O = 100:1. The relatively large difference of the plots from the regression line in the shorter mixing times is due to the decomposition of $H_3\mathbf{1}^{3+}$ during the measurements.

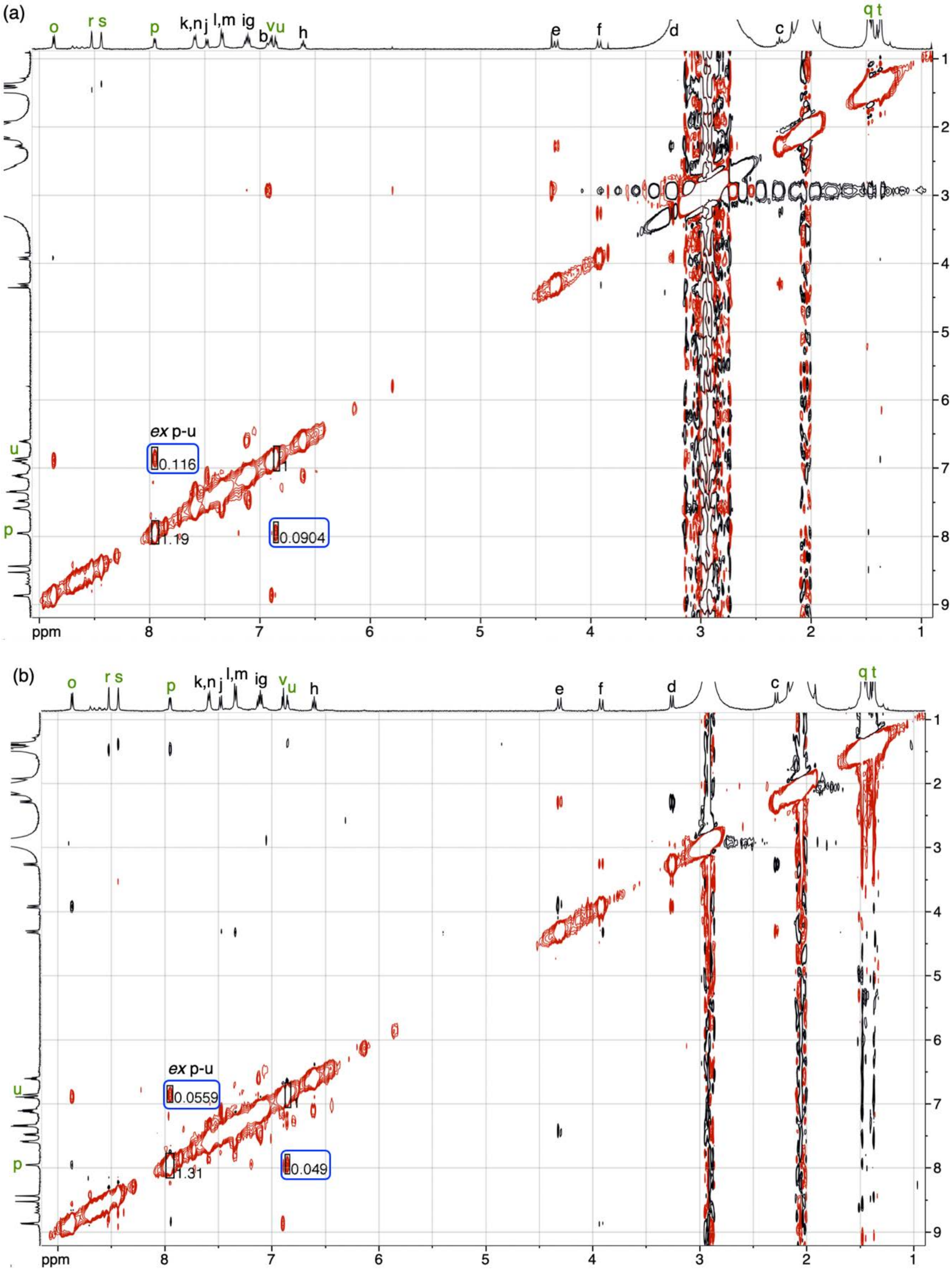

Figure S61. ^{1}H-^{1}H EXSY NMR spectra of $H_3\mathbf{1}^{3+}$ (500 MHz, 300 K, mixing time $\tau_m = 0.3$ s, 0.27 mM). Blue squares indicate the chemical exchange signals; (a) in acetone-d_6:H_2O = 100:1 and (b) in acetone-d_6:D_2O = 100:1.

4.6 Kinetic isotope effect on the helicity inversion of $H_3\mathbf{1}^{3+}$ with methanol

The kinetic isotope effects of the helicity inversion of $H_3\mathbf{1}^{3+}$ was investigated with methanol.

To a wet acetone-d_6 solution (0.6 mL) of $H_6\mathbf{1}\cdot 6OTf$ (0.43 mg, 0.16 μmol, 1.0 equiv.) was added solid Na_2CO_3 (0.72 mg, 6.8 μmol, 43 equiv.). This suspension was sonicated at room temperature for 5 min and centrifuged to remove the precipitate. The supernatant (500 μL) was transferred to an NMR tube and CH_3OH (5 μL) was added. 2D ^1H-^1H EXSY NMR spectroscopy of the acetone-d_6:MeOH = 100:1 solution of $H_3\mathbf{1}^{3+}$ (505 μL, 0.26 mM) was conducted. The inversion rate was estimated using the chemical exchange signals between H_o and H_v.

Similarly, an acetone-d_6:CD_3OD = 100:1 solution of $H_3\mathbf{1}^{3+}$ (505 μL, 0.26 mM) was also prepared and subjected to 2D ^1H-^1H EXSY spectroscopy. The inversion rate was estimated using the chemical exchange signals between H_o and H_v. The rate constants in acetone-d_6:CH_3OH = 100:1 (k_H) and acetone-d_6:CD_3OD = 100:1 (k_D) were then compared to evaluate the kinetic isotope effect on the helicity inversion of $H_3\mathbf{1}^{3+}$. As we described in the preparation of $H_3\mathbf{1}^{3+}$, 3 equiv. of NaOTf remained in the acetone-d_6 solution of $H_3\mathbf{1}^{3+}$.

The observed k_H/k_D value (k_H/k_D = 1.73) also confirms that the proton transfer via hydrogen recombination is involved in the rate-determining step of the helicity inversion of $H_3\mathbf{1}^{3+}$, although the possibility that other inversion mechanisms may also be involved cannot be ruled out.

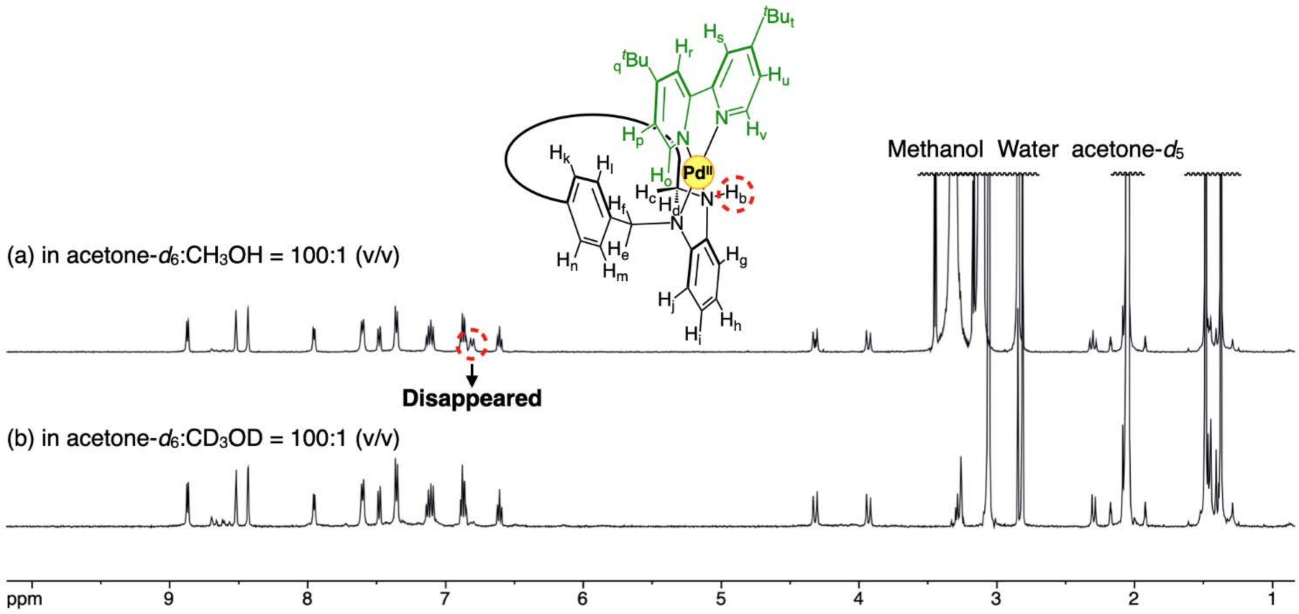

Figure S62. ^1H NMR spectra of $H_3\mathbf{1}^{3+}$ (a) in acetone-d_6:CH_3OH = 100:1 (v/v) and (b) in acetone-d_6:CD_3OD = 100:1 (v/v) (500 MHz, 300 K).

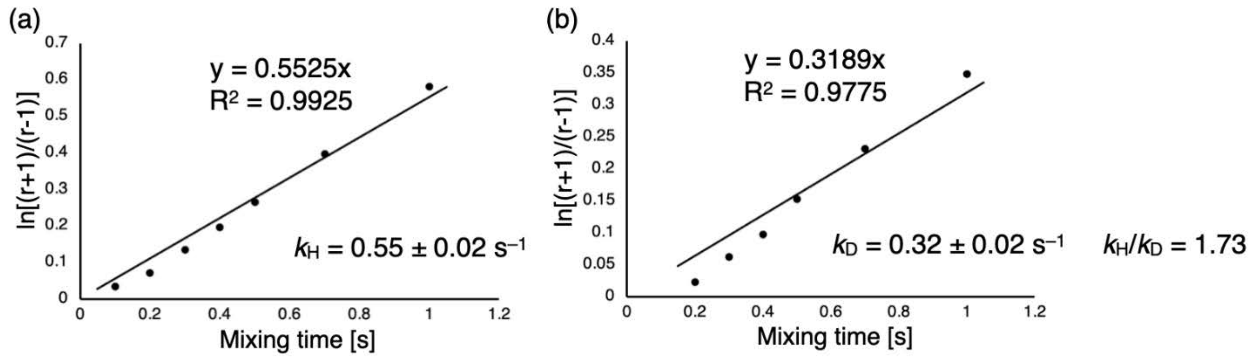

Figure S63. Plots of ln[(r + 1)/(r – 1)] versus mixing time τ_m based on ^{1}H-^{1}H EXSY spectra of $H_3\mathbf{1}^{3+}$ (500 MHz, 300 K, 0.26 mM) recorded at different mixing times (0.1–1.0 s), with fitting lines with based on chemical exchange signals between H_o and H_v: (a) in acetone-d_6:CH_3OH = 100:1 and (b) in acetone-d_6:CD_3OD = 100:1.

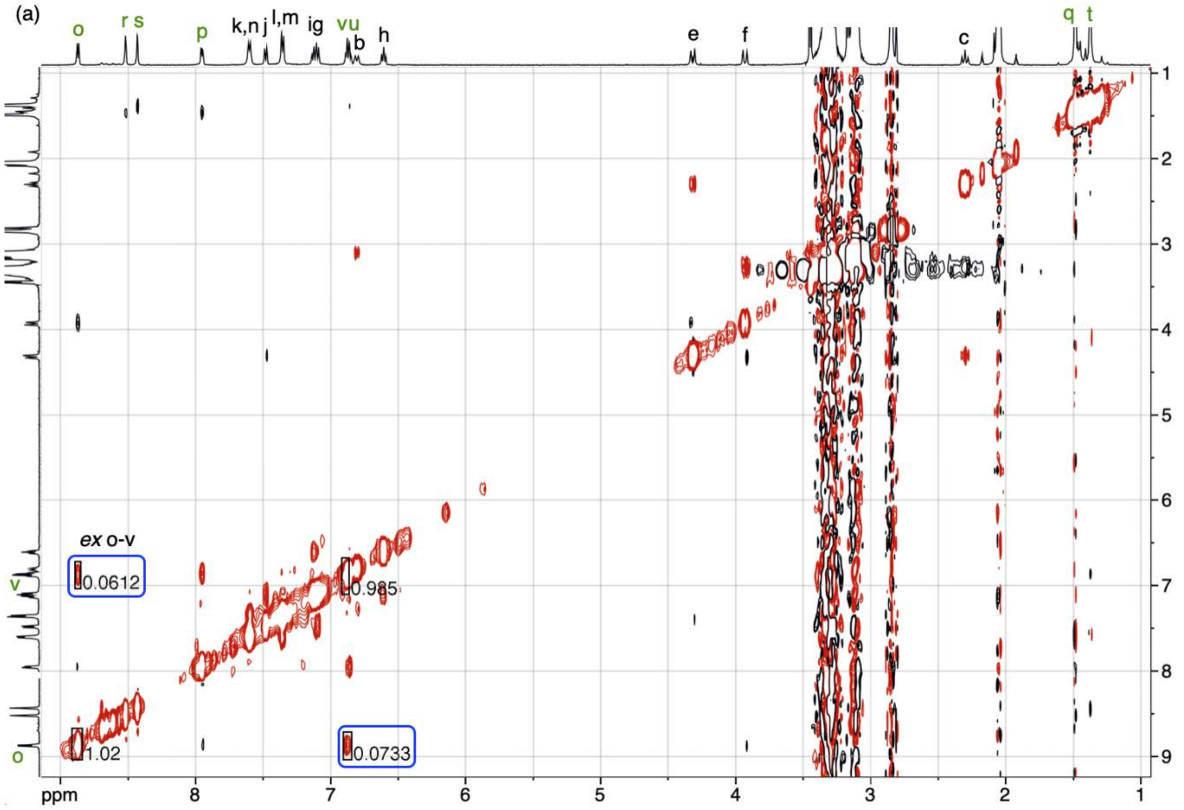

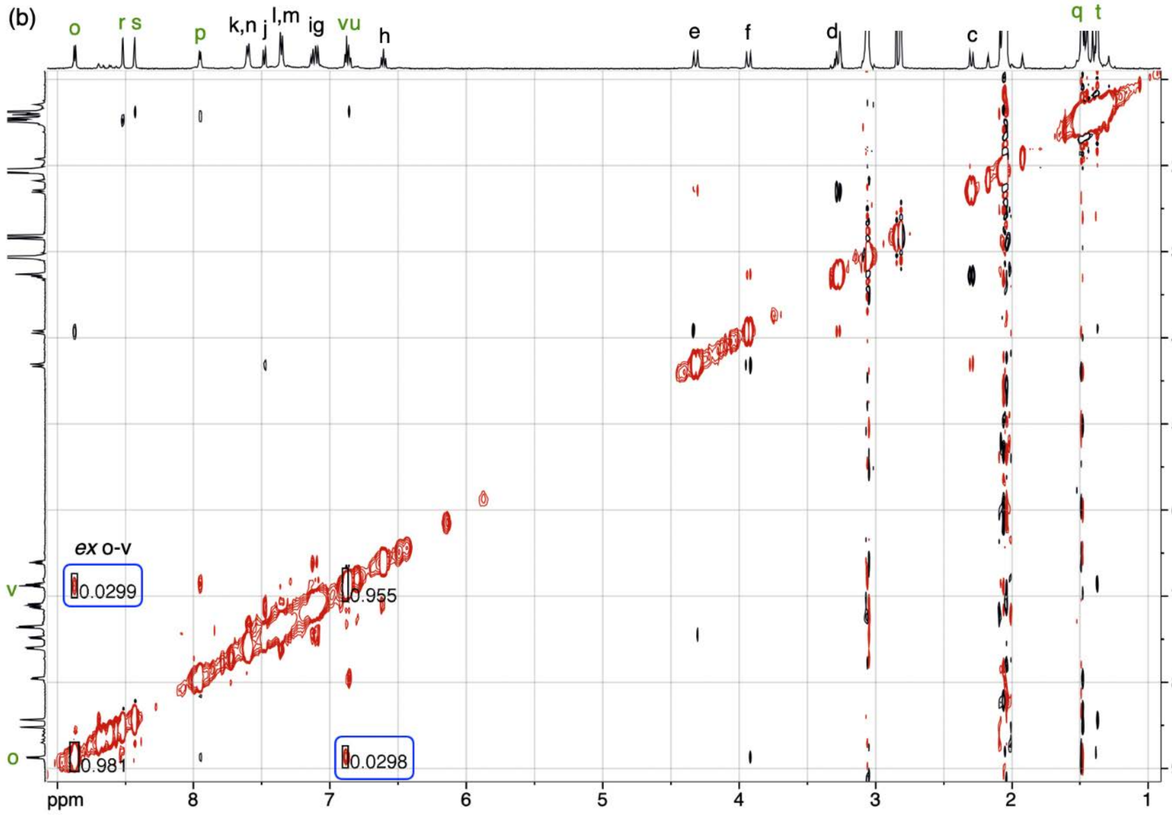

Figure S64. ^{1}H-^{1}H EXSY NMR spectra of $H_3\mathbf{1}^{3+}$ (500 MHz, 300 K, mixing time τ_m = 0.3 s, 0.26 mM). Blue boxes indicate the chemical exchange signals; (a) in acetone-d_6:CH_3OH = 100:1 and (b) in acetone-d_6:CD_3OD = 100:1.

4.7 Kinetic isotope effect on the helicity inversion of $H_6\mathbf{1}^{6+}$

As a control experiment, the kinetic isotope effect of $H_6\mathbf{1}^{6+}$ was investigated in the same manner as $H_3\mathbf{1}^{3+}$.

To an undried acetone-d_6 solution (0.5 mL) of $H_6\mathbf{1}\cdot 6OTf$ (0.37 mg, 0.14 μmol) was added H_2O or D_2O (5 μL). 2D ^{1}H-^{1}H EXSY NMR spectroscopy of these acetone-d_6:H_2O/D_2O = 100:1 solutions of $H_6\mathbf{1}^{6+}$ (505 μL, 0.27 mM) was conducted.

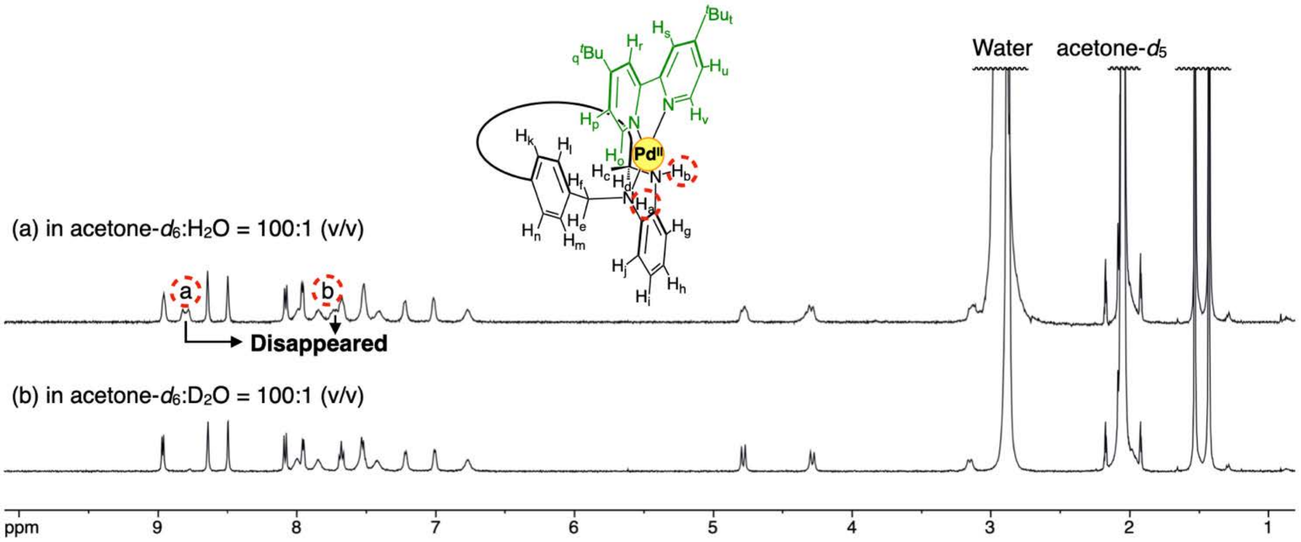

Figure S65. ^{1}H NMR spectra of $H_6\mathbf{1}^{6+}$ (a) in acetone-d_6:H_2O = 100:1 (v/v) and (b) in acetone-d_6:D_2O = 100:1 (v/v) (500 MHz, 300 K).

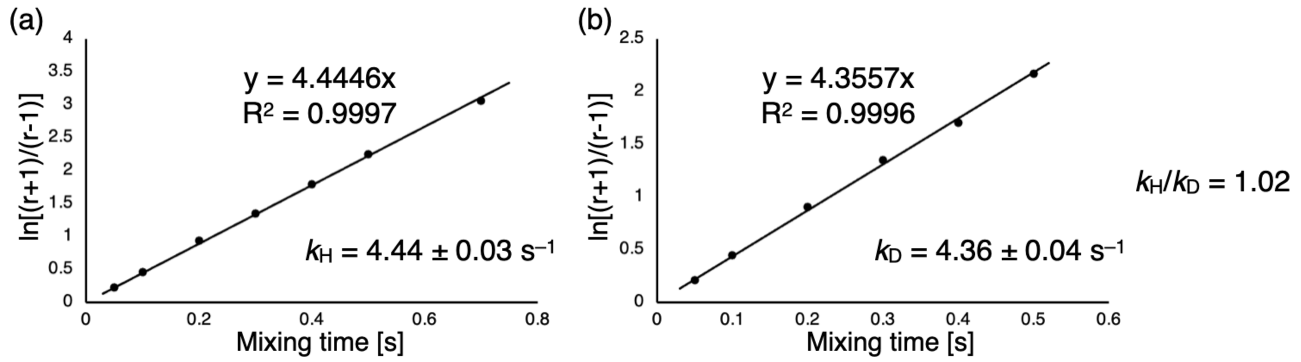

Figure S66. Plots of ln[$(r + 1)/(r – 1)$] versus mixing time τ_m for $H_6\mathbf{1}^{6+}$ from ^{1}H-^{1}H EXSY spectra (500 MHz, 300 K, 0.27 mM) recorded with different mixing times (0.05–0.7 s) and their fitting line with a formula based on chemical exchange signals between H_o and H_v; (a) in acetone-d_6:H_2O = 100:1 and (b) in acetone-d_6:D_2O = 100:1.

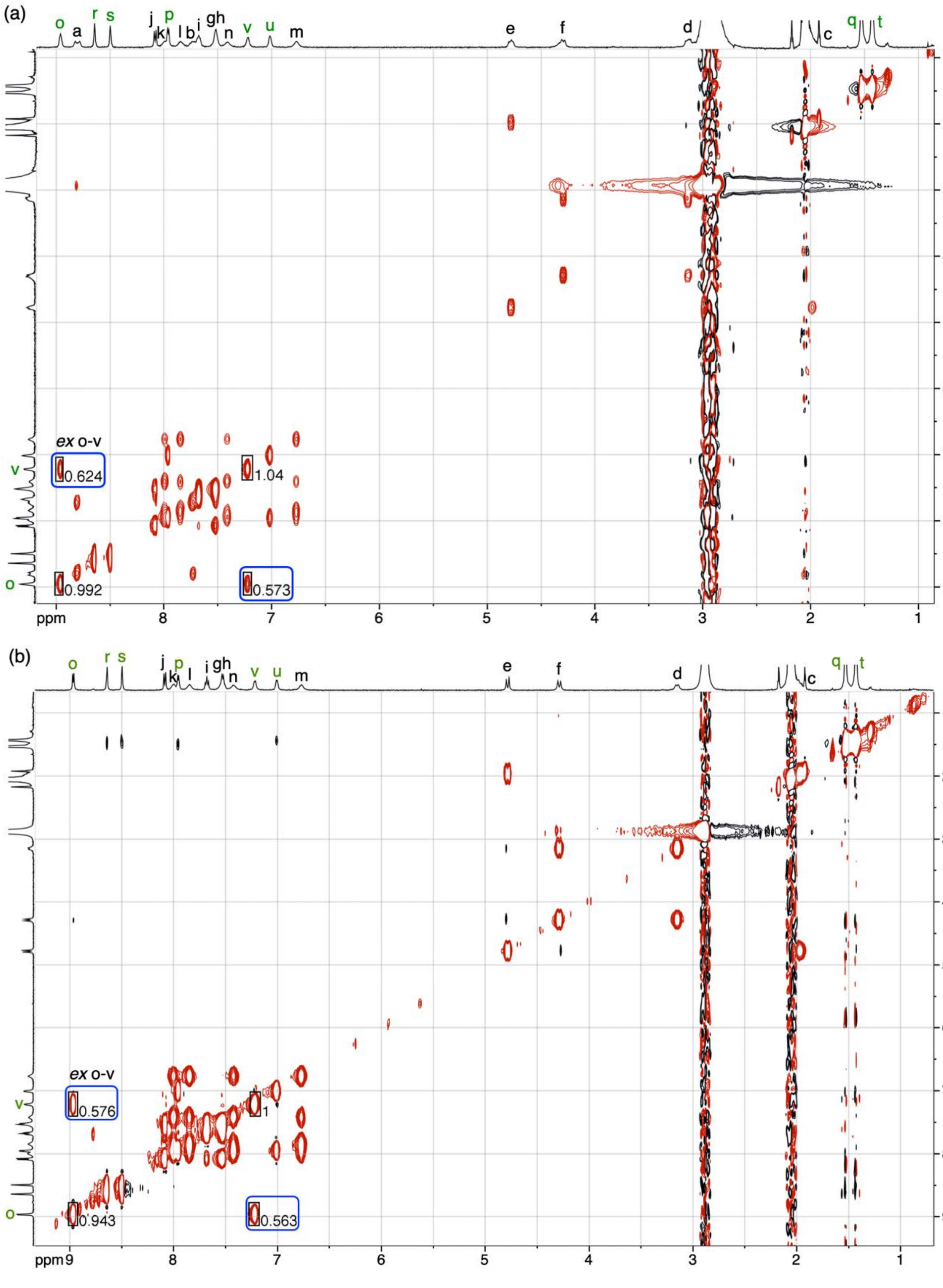

Figure S67. ${}^{1}H$-${}^{1}H$ EXSY NMR spectra of $H_6\mathbf{1}^{6+}$ (500 MHz, 300 K, mixing time τ_m = 0.3 s, 0.27 mM). Blue squares indicate the chemical exchange signals; (a) in acetone-d_6:H_2O = 100:1 and (b) in acetone-d_6:D_2O = 100:1.

4.8 The effect of the addition of CH_3CN on the helicity inversion rate of $H_3\mathbf{1}^{3+}$

Similar to the water and methanol cases, CH_3CN was added to an undried acetone-d_6 solution of $H_3\mathbf{1}^{3+}$ to investigate the effect of the aprotic solvent.

To an undried acetone-d_6 solution (0.6 mL) of $H_6\mathbf{1}\cdot 6OTf$ (0.41 mg, 0.15 μmol, 1.0 equiv.) was added Na_2CO_3 (0.69 mg, 6.5 μmol, 43 equiv.). This suspension was sonicated at room temperature for 5 min and centrifuged to remove the precipitate. The supernatant (500 μL) was transferred to an NMR tube and CH_3CN (5 μL) was added. 2D ^1H-^1H EXSY NMR spectroscopy of this acetone-d_6:CH_3CN = 100:1 solution of $H_3\mathbf{1}^{3+}$ (505 μL, 0.25 mM) was conducted. The chemical exchange signals between H_o and H_v were used to estimate the inversion rate.

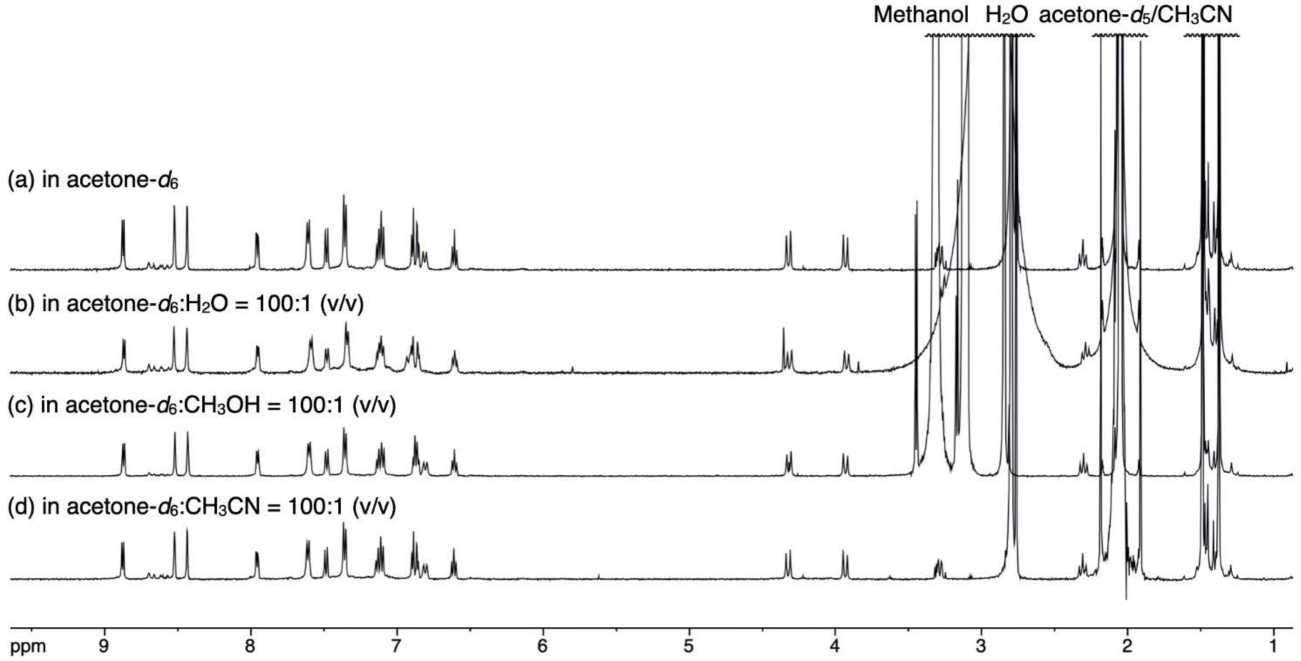

Figure S68. ^1H NMR spectra of $H_3\mathbf{1}^{3+}$ (500 MHz, 300 K) in (a) acetone-d_6, (b) acetone-d_6:H_2O = 100:1 (v/v), (c) acetone-d_6:CH_3OH = 100:1 (v/v), and (d) acetone-d_6:CH_3CN = 100:1 (v/v).

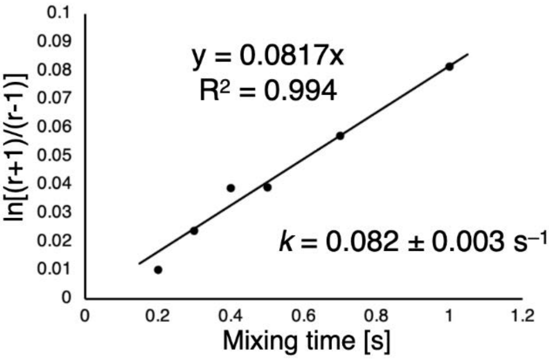

Figure S69. Plots of $\ln[(r+1)/(r-1)]$ versus mixing time τ_m for $H_3\mathbf{1}^{3+}$ from ^1H-^1H EXSY spectra (500 MHz, 300 K) recorded with different mixing times (0.2–1.0 s) and their fitting line with a formula based on chemical exchange signals between H_o and H_v in acetone-d_6:CH_3CN = 100:1.

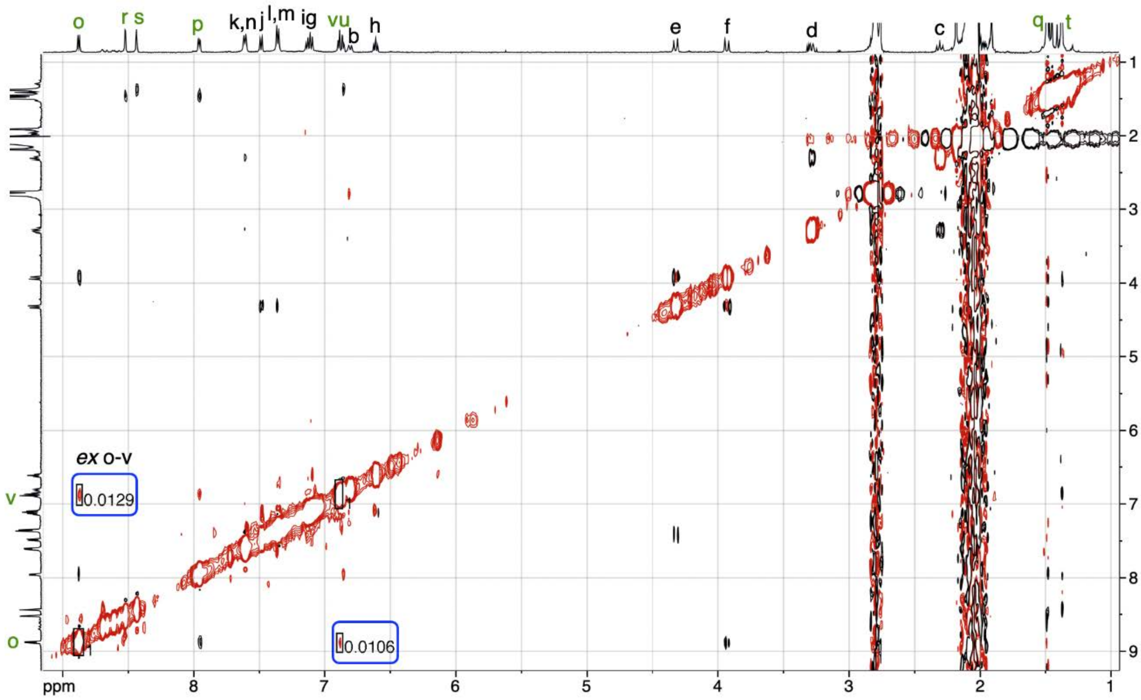

Figure S70. ^{1}H-^{1}H EXSY NMR spectra of $H_3\mathbf{1}^{3+}$ (500 MHz, 300 K, mixing time τ_m = 0.3 s). Blue squares indicate the chemical exchange signals in acetone-d_6:CH_3CN = 100:1.

4.9 Estimation of the helicity inversion rate of $H_3\mathbf{1}^{3+}$ deprotonated by proton sponge

To investigate the effect of the base on the helicity inversion rate of $H_3\mathbf{1}^{3+}$, proton sponge was used as a base for EXSY analysis.

To an undried acetone-d_6 solution (0.48 mL) of $H_6\mathbf{1}\cdot 6OTf$ (0.23 mg, 0.084 µmol, 0.18 mM, 1.0 equiv.) was added an acetone-d_6 solution of proton sponge (15 mM, 1.0 µL, 4.2 equiv. calculated by the integral ratio in the ^{1}H NMR spectrum). Then, 2D ^{1}H-^{1}H EXSY was conducted. The chemical exchange signals between H_c and H_e were used to estimate the inversion rate. The formation of $H_3\mathbf{1}^{3+}$ was confirmed by the chemical shift and the integral ratio in the ^{1}H NMR spectroscopy even in the presence of a little excess amount of proton sponge as discussed in Supplementary section 2.6.

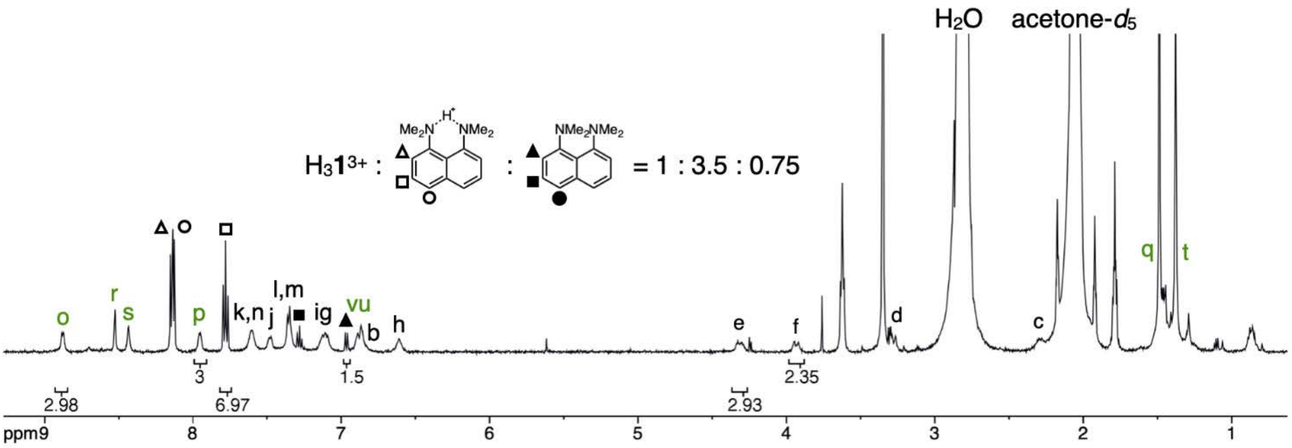

Figure S71. ^{1}H NMR spectrum of $H_3\mathbf{1}^{3+}$ deprotonated by proton sponge (500 MHz, acetone-d_6, 300 K, 0.18 mM).

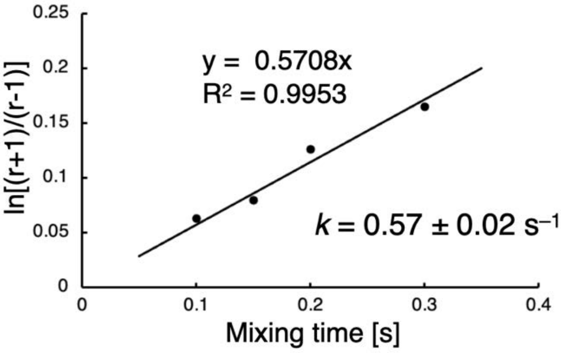

Figure S72. Plots of $\ln[(r+1)/(r-1)]$ versus mixing time τ_m for $H_3\mathbf{1}^{3+}$ from ^{1}H-^{1}H EXSY spectra (500 MHz, 300 K, 0.18 mM) recorded with different mixing times (0.1–0.3 s) and their fitting line with a formula based on chemical exchange signals between H_c and H_e.

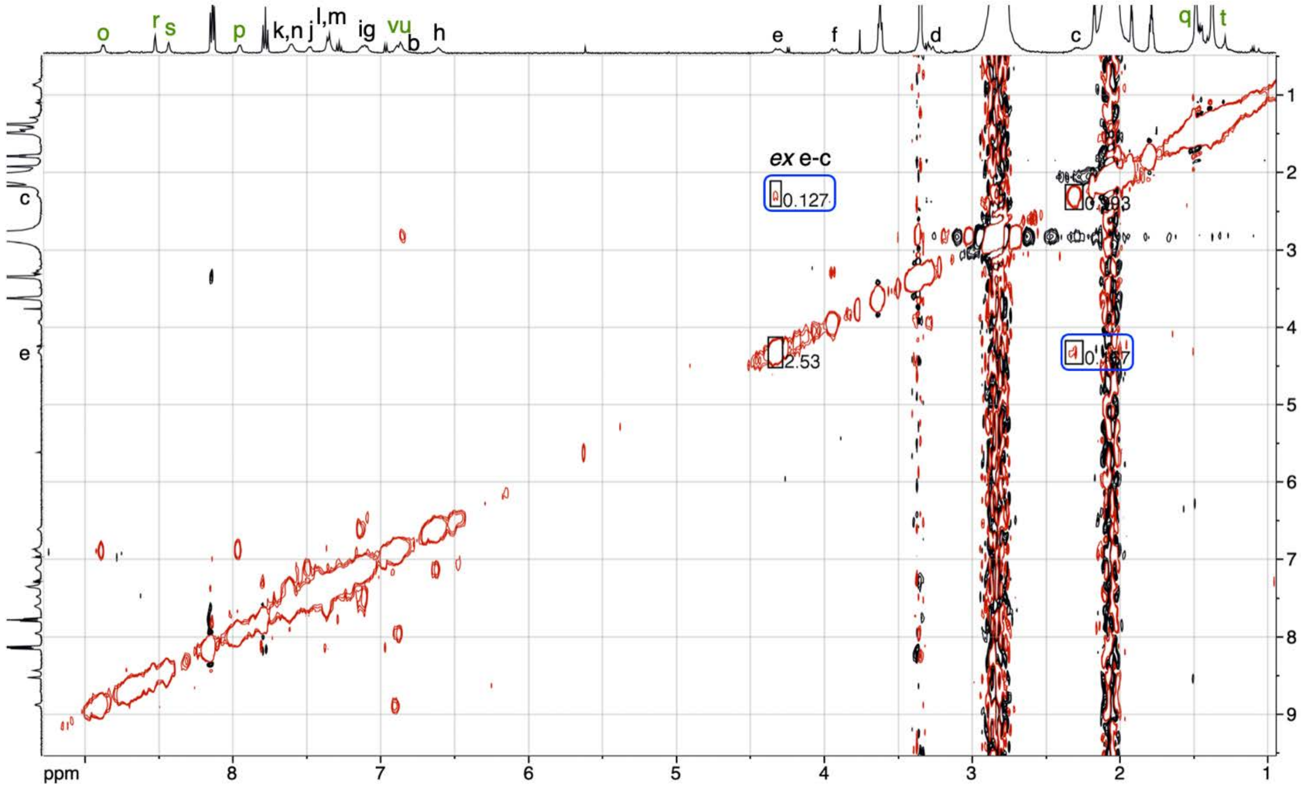

Figure S73. ^{1}H-^{1}H EXSY NMR spectra of $H_3\mathbf{1}^{3+}$ (500 MHz, acetone-d_6, 300 K, mixing time τ_m = 0.3 s, 0.18 mM). Blue squares indicate the chemical exchange signals.

Base	Na_2CO_3					proton sponge
Solvent	Distilled acetone-d_6	Undried acetone-d_6	acetone-d_6:**CH_3CN** = 100:1 (v/v)	acetone-d_6:**CH_3OH** = 100:1 (v/v)	acetone-d_6:**H_2O** = 100:1 (v/v)	Undried acetone-d_6
Helicity inversion rate	0.0593 s^{-1}	0.167 s^{-1}	0.082 s^{-1}	0.55 s^{-1}	0.704 s^{-1}	0.57 s^{-1}

Figure S74. Table of the helicity inversion rates of $H_3\mathbf{1}^{3+}$ measured under various conditions. The acetone-d_6 used in the mixed solvent systems was used without distillation.

5. Computational study

5.1 Calculation methods and conditions

DFT calculation

All DFT calculations were performed using Gaussian 16 [rev.C01] and [rev.C02] program.[7] The B3LYP-D3 functional[8,9,10] and mixed basis sets of def2svp[11] for Pd and 6-31G(d)[12,13] for other atoms were used for geometry optimization, vibrational frequency, and TD-DFT calculations of $H_3\mathbf{1}^{3+}$, $H_3\mathbf{1}_{\mathbf{iso}}{}^{3+}$, $[H_6\mathbf{1}\cdot OTf]^{5+}$ and **1**. The mixed basis sets of def2tzvp[11] for Pd, 6-311+G(d)[14,15] for N, and 6-311G(d)[14] for H and C were used in single point calculations of $H_3\mathbf{1}^{3+}$ and $H_3\mathbf{1}_{\mathbf{iso}}{}^{3+}$. No imaginary frequency was found for all optimized structures, confirming that each optimized structure reached a local minimum. The results of TD-DFT calculations were convoluted using GaussSum software to create simulated UV-vis spectra.[16] The solvent effect was included in TD-DFT and single point energy calculations by using SMD model.[17]

The structures of $H_3\mathbf{1}^{3+}$ and **1** were resulted in C_3-symmetry in the optimization and frequency calculations, but the point group was reduced to C_1-symmetry to complete the calculations correctly in the TD-DFT and single point calculations in acetone using the SMD model. In principle, molecules in C_3-symmetry have the molecular orbitals (MOs) of *a* and *e* symmetry. The symmetry of MOs calculated by DFT in SMD was determined by the orbital energy levels and the location of electron density, and the symmetry of excited states was determined by the calculated transition energies and oscillator strengths.

The orbital composition analyses with Mulliken partition were performed using Multifwn software Version 3.8(dev).[18]

The optimized structures were displayed using Mercury and Pymol software packages, and the Frontier orbitals were visualized using Avogadro1.2 program.[19]

NBO calculation

Natural bond orbital (NBO) calculations of the optimized structure were performed using NBO7.0 package[20] embedded in the Gaussian16 programs. The NBOs were visualized with the Avogadro1.2 program. The calculation conditions such as functional, basis sets and the solvent effects were the same as the single point calculation.

5.2 DFT calculations of $H_3\mathbf{1}^{3+}$

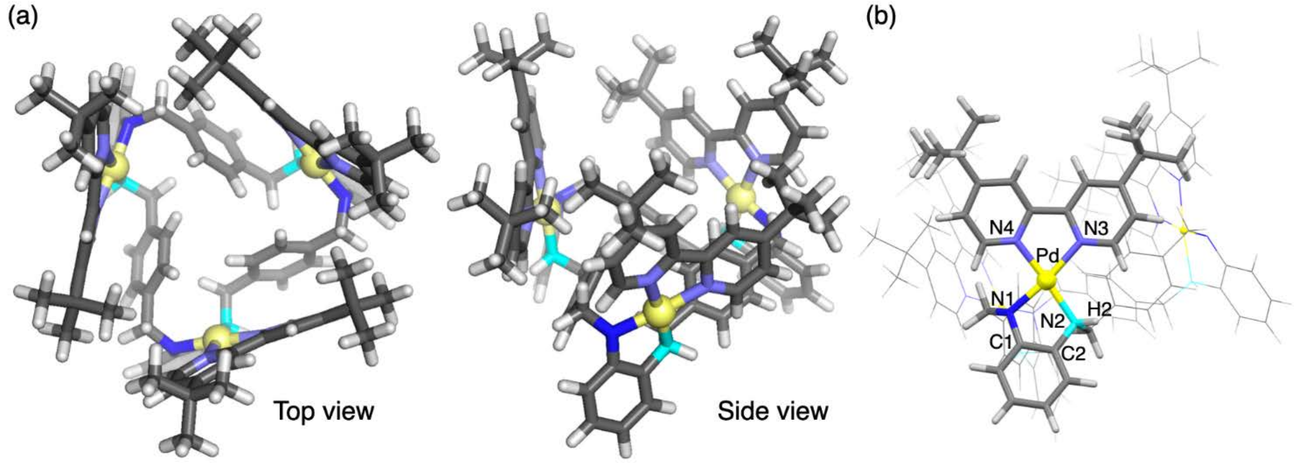

(c)

Bond length [Å]	**Pd-N1**	Pd-N2	Pd-N3	Pd-N4	**C1-N1**	C2-N2	N1-H2
$[H_3\mathbf{1}]^{3+}$	**2.006**	2.109	2.117	2.053	**1.413**	1.474	3.555

Figure S75. (a) Top and side views of the optimized structure of $H_3\mathbf{1}^{3+}$ [B3LYP-D3/def2svp for Pd, 6-31G* for other atoms], (b) a structure of $H_3\mathbf{1}^{3+}$ indicating the atom labels, and (c) table of bond lengths around a metal center and an *ortho*-phenylenediamine moiety.

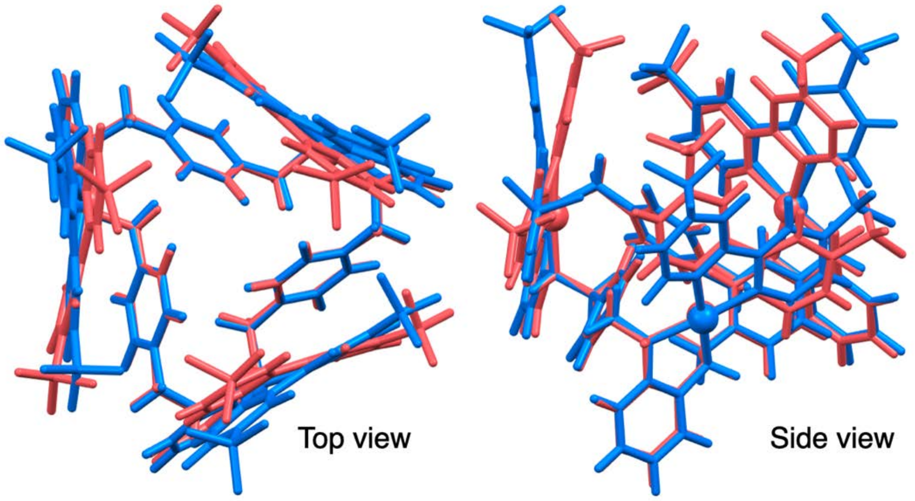

Figure S76. Overlaid views of (red) the crystal structure of $H_6\mathbf{1}^{6+}$ (anions and solvent molecules are omitted for clarity) and (blue) the optimized structure of $H_3\mathbf{1}^{3+}$. Protons of *tert*-butyl groups are omitted for clarity.

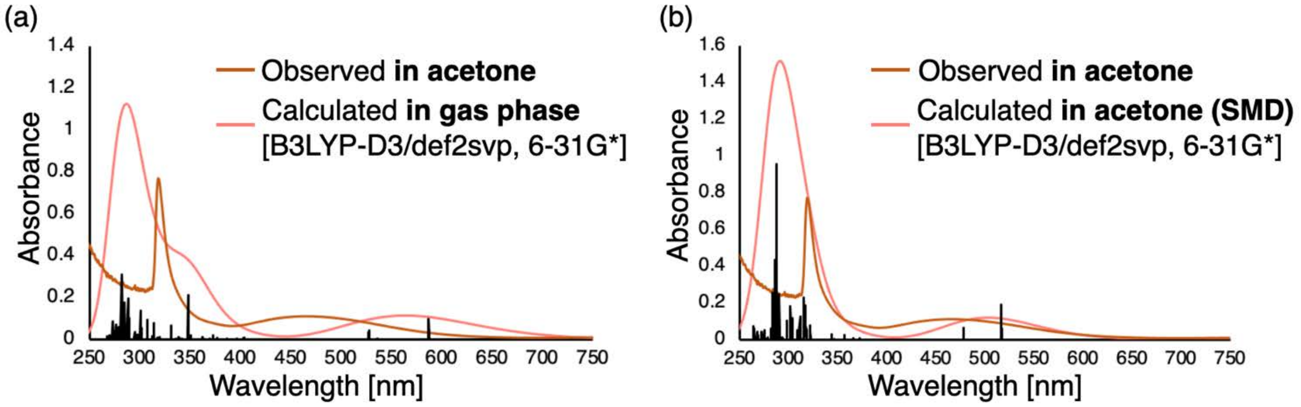

Figure S77. (brown line) The observed UV-vis spectra of $H_3\mathbf{1}^{3+}$ in acetone and (pink and black lines) calculated UV-vis spectra of $H_3\mathbf{1}^{3+}$ [B3LYP-D3/def2svp for Pd, 6-31G* for other atoms] (a) in gas phase and (b) in acetone (SMD model).

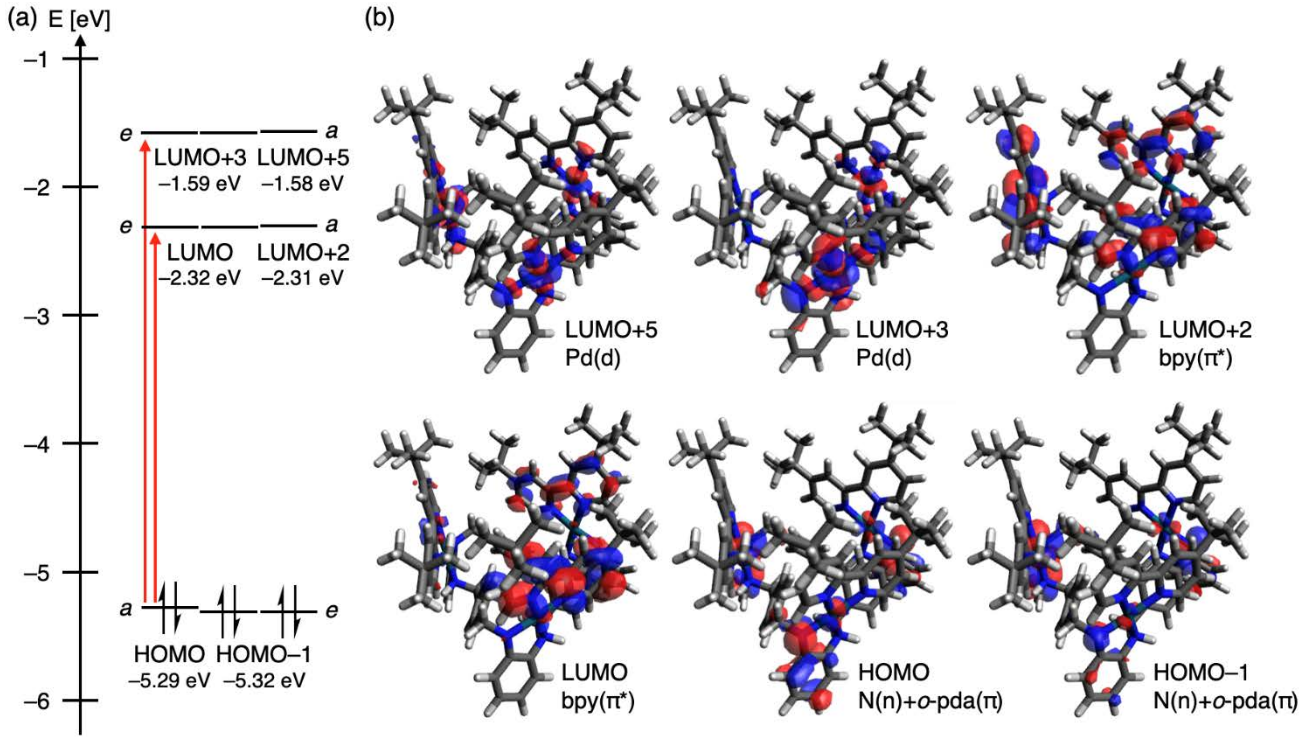

(c)

State	ΔE [eV]	λ [nm]	f	Character
E	2.399	516.8	0.029	HOMO(*a*)->LUMO(*e*) (39%), HOMO–1(*e*)->LUMO(*e*) (27%), HOMO–1(*e*)->LUMO+2(*a*) (26%)
A	2.401	516.5	0.047	HOMO–1(*e*)->LUMO(*e*) (60%), HOMO(*a*)->LUMO+2(*a*) (34%)
A	2.592	478.3	0.017	HOMO–1(*e*)->LUMO+3(*e*) (59%), HOMO(*a*)->LUMO+5(*a*) (29%)
E	2.594	478.0	0.027	HOMO–1(*e*)->LUMO+3(*e*) (38%), HOMO(*a*)->LUMO+3(*e*) (31%), HOMO–1(*e*)->LUMO+5(*a*) (21%)

(d)	LUMO+5	LUMO+3	LUMO+2	LUMO	HOMO	HOMO–1
Pd	48.8%	48.9%	2.9%	2.9%	10.5%	10.6%
N(n)	14.2%	14.3%	0.5%	0.5%	43.6%	43.9%
o-pda	2.8%	2.8%	0.2%	0.2%	32.6%	33.1%
bpy	21.8%	21.7%	92.7%	93.2%	2.8%	2.9%

Figure S78. (a) The orbital energy levels of $H_3\mathbf{1}^{3+}$ based on DFT calculation in acetone (SMD model) [B3LYP-D3/def2svp for Pd, 6-31G* for other atoms], (b) calculated frontier orbitals of $H_3\mathbf{1}^{3+}$ (isosurface value = 0.025), (c) assignment of the excited states with transition energy (ΔE), wavelength (λ) and oscillator strength (f). Generally, each E-excited state is doubly-degenerated in C_3 symmetry. In table (c), one of the two transition energies with lower energy was shown for an E-excited state due to the breakdown of symmetry in TD-DFT (SMD), while the oscillator strength of the E-state was listed as the sum of two components. (d) Orbital composition analysis with Mulliken partition of LUMO+5, LUMO+3, LUMO+2, LUMO, HOMO and HOMO–1 of $H_3\mathbf{1}^{3+}$ in acetone (SMD). Pd, N(n), *o*-pda and bpy indicate Pd atoms, lone pairs of amine nitrogen atoms, *ortho*-phenylenediamine and bipyridine moieties, respectively.

5.3 TD-DFT calculations using other basis sets and functionals

To check the dependence of the DFT functionals, the basis sets, and the solvent effects on the simulated UV-vis spectra, geometry optimization and TD-DFT calculations were conducted using the B3LYP-D3, CAM-B3LYP,[21] M06-D3,[22,10] MN15,[23] and ωB97[24] functionals and the 6-31+G(d)[12,13,15] basis sets for N atoms. In these calculations, the substitution of bipyridine moieties was simplified or modelled from *tert*-butyl to methyl groups to reduce the calculation cost.

Comparing these results, it was found that the calculated spectra highly depended on the solvent effects and the functionals, while the diffuse function of nitrogen atoms had minor effect on the calculated spectra. Among the tested calculation conditions, the B3LYP-D3 functional with solvent effect using the SMD model gave a better result than others. Thus, these calculation conditions were applied to the computational studies of $H_3\mathbf{1}^{3+}$, $H_3\mathbf{1}_{\mathbf{iso}}^{3+}$ and $[H_6\mathbf{1}\cdot OTf]^{5+}$.

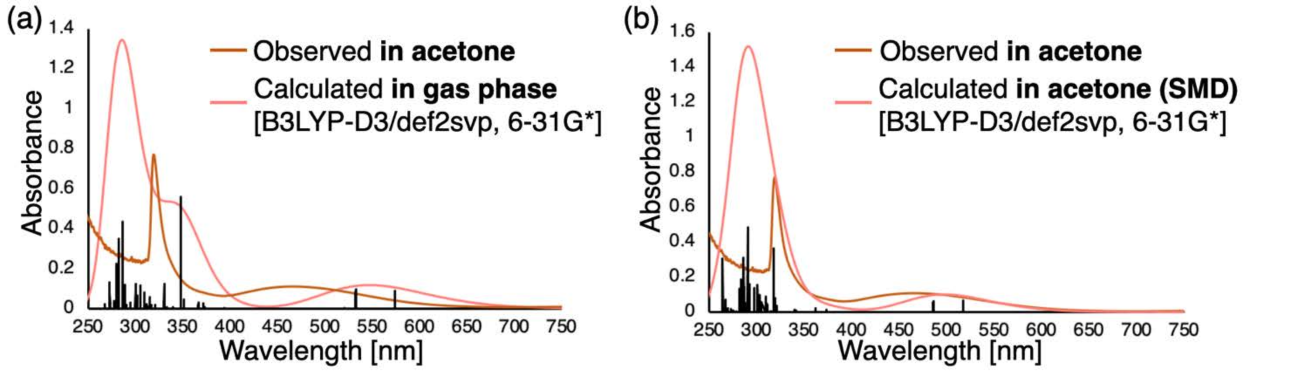

Figure S79. (brown line) The observed UV-vis spectra of $H_3\mathbf{1}^{3+}$ in acetone and (pink and black lines) calculated UV-vis spectra [B3LYP-D3/def2svp for Pd, 6-31G* for other atoms], (a) in gas phase and (b) in acetone (SMD model).

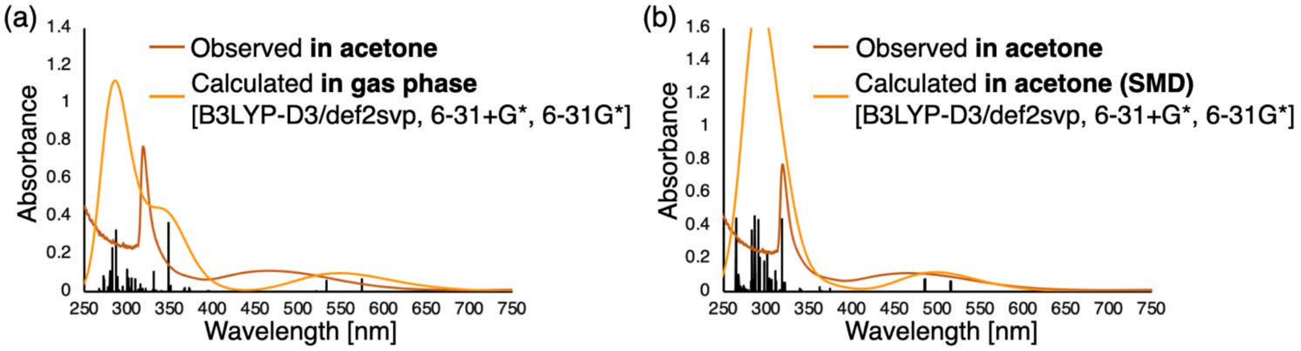

Figure S80. (brown line) The observed UV-vis spectra of $H_3\mathbf{1}^{3+}$ in acetone and (orange and black lines) calculated UV-vis spectra [B3LYP-D3/def2svp for Pd, 6-31+G* for N, 6-31G* for other atoms], (a) in gas phase and (b) in acetone (SMD model).

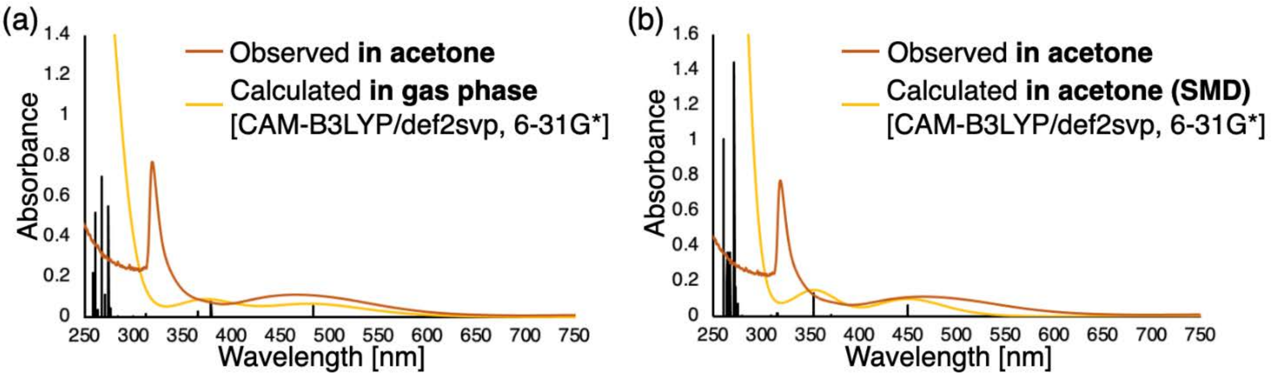

Figure S81. (brown line) The observed UV-vis spectra of $H_3\mathbf{1}^{3+}$ in acetone and (yellow and black lines) calculated UV-vis spectra [CAM-B3LYP/def2svp for Pd, 6-31G* for other atoms], (a) in gas phase and (b) in acetone (SMD model). The calculated transitions around 450 and 350 nm were assigned to the ligand-to-metal charge transfer (LMCT) and the interligand charge transfer (ILCT), respectively, as discussed in the main text.

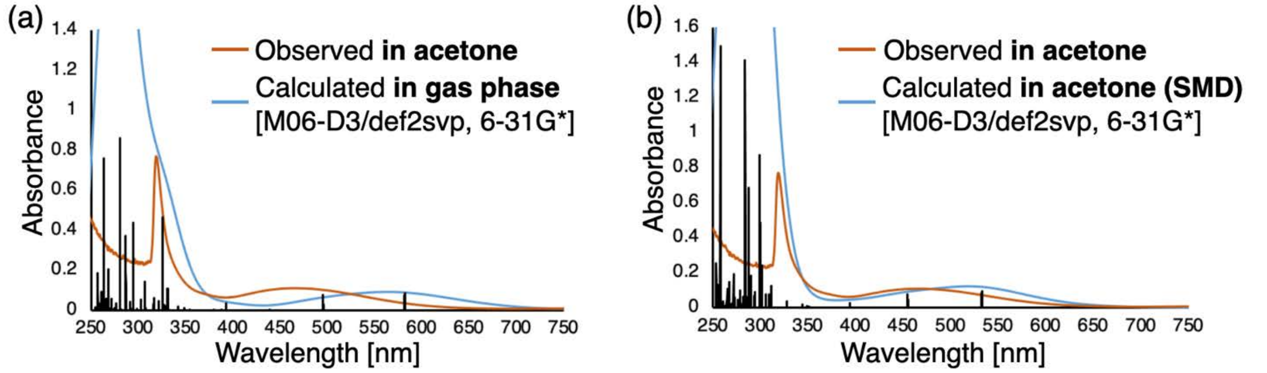

Figure S82. (brown line) The observed UV-vis spectra of $H_3\mathbf{1}^{3+}$ in acetone and (pale blue and black lines) calculated UV-vis spectra [M06-D3/def2svp for Pd, 6-31G* for other atoms], (a) in gas phase and (b) in acetone (SMD model).

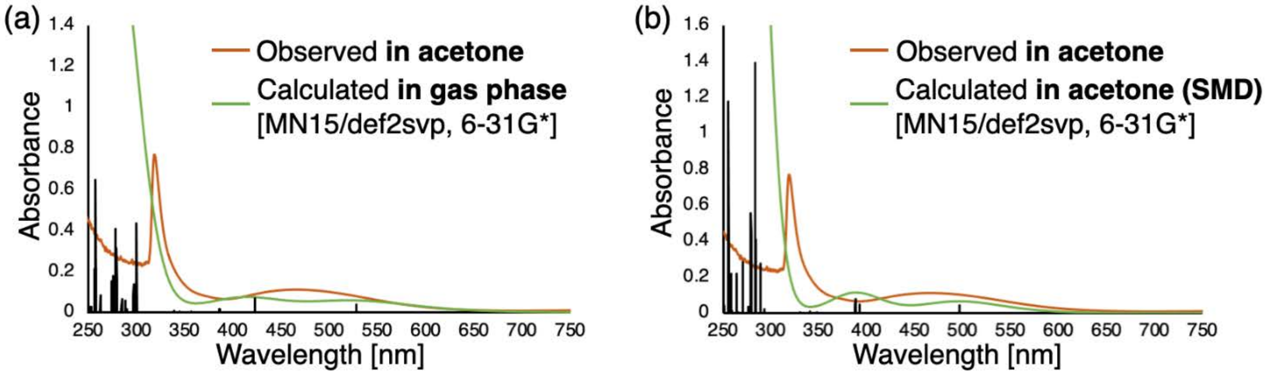

Figure S83. (brown line) The observed UV-vis spectra of $H_3\mathbf{1}^{3+}$ in acetone and (green and black lines) calculated UV-vis spectra [MN15/def2svp for Pd, 6-31G* for other atoms], (a) in gas phase and (b) in acetone (SMD model).

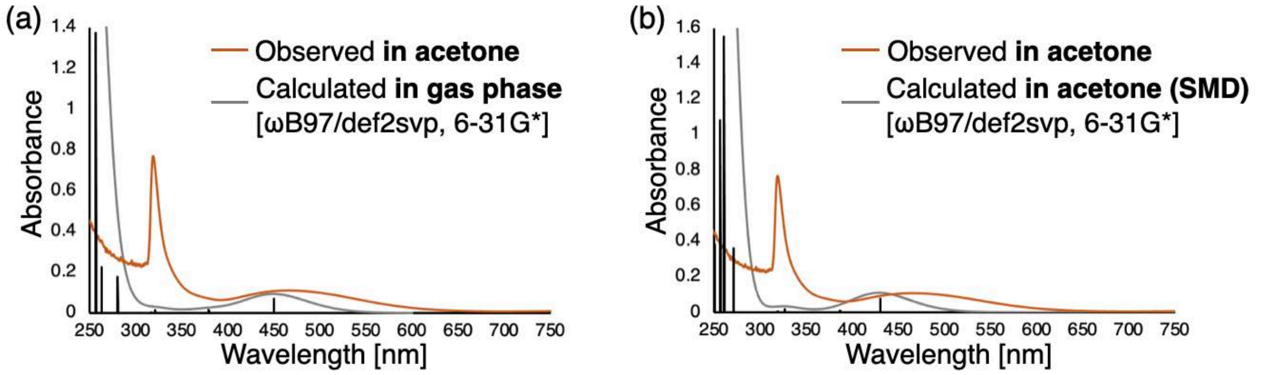

Figure S84. (brown line) The observed UV-vis spectra of $H_3\mathbf{1}^{3+}$ in acetone and (grey and black lines) calculated UV-vis spectra [ωB97/def2svp for Pd, 6-31G* for other atoms], (a) in gas phase and (b) in acetone (SMD model). The calculated transitions around 430 nm were assigned to LMCT.

5.4 Comparison between $H_3\mathbf{1}^{3+}$ and $H_3\mathbf{1}_{\mathbf{iso}}^{3+}$

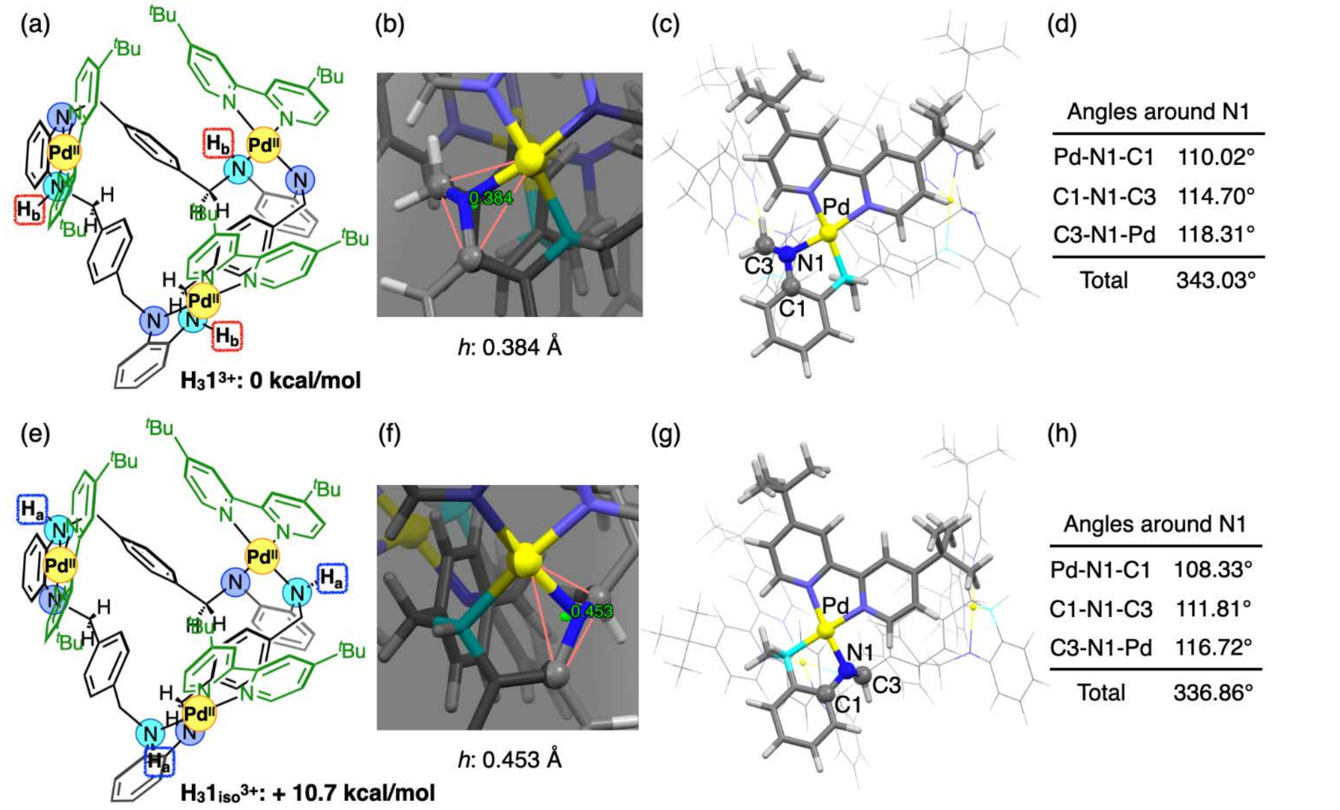

(d)

Angles around N1	
Pd-N1-C1	110.02°
C1-N1-C3	114.70°
C3-N1-Pd	118.31°
Total	343.03°

(h)

Angles around N1	
Pd-N1-C1	108.33°
C1-N1-C3	111.81°
C3-N1-Pd	116.72°
Total	336.86°

Figure S85. (a) The chemical formula of $H_3\mathbf{1}^{3+}$ with the relative Gibbs free energy, (b) an enlarged figure of the optimized $H_3\mathbf{1}^{3+}$ with the height *h* of the deprotonated nitrogen atom from the Pd-C-C plane, (c) the optimized structure of $H_3\mathbf{1}^{3+}$ with labels, (d) a table of angles around the N1 atom. (e) The chemical formula of $H_3\mathbf{1}_{\mathbf{iso}}^{3+}$ with the relative Gibbs free energy to $H_3\mathbf{1}^{3+}$, (f) an enlarged figure of the optimized $H_3\mathbf{1}_{\mathbf{iso}}^{3+}$ with the height *h* of the deprotonated nitrogen atom from the Pd-C-C plane, (g) the optimized structure of $H_3\mathbf{1}_{\mathbf{iso}}^{3+}$ with labels and (h) a table of angles around the N1 atom. Structures of $H_3\mathbf{1}^{3+}$ and $H_3\mathbf{1}_{\mathbf{iso}}^{3+}$ were obtained by DFT calculations [B3LYP-D3/def2tzvp for Pd, 6-311+G* for N, 6-311G* for other atoms in acetone (SMD model)//B3LYP-D3/def2svp for Pd, 6-31G* for other atoms].

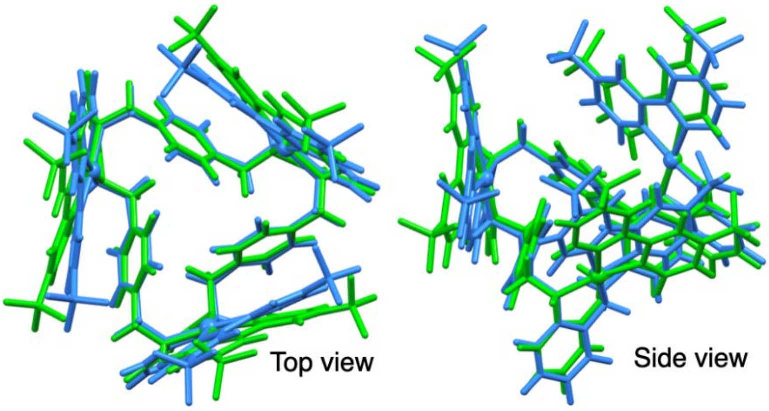

Figure S86. Overlaid view of (blue) the optimized structure of $H_3\mathbf{1}^{3+}$ and (green) the optimized structure of another deprotonation isomer $H_3\mathbf{1}_{\mathbf{iso}}^{3+}$. Protons of *tert*-butyl groups are omitted for clarity.

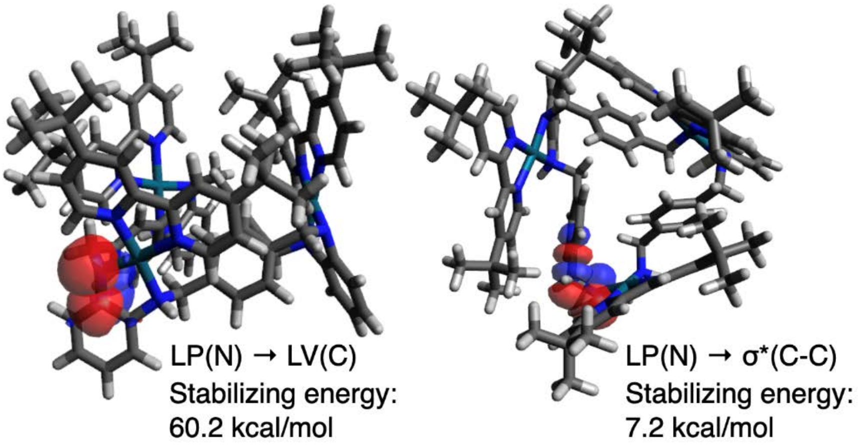

Figure S87. Superposition of (left) a donor (lone pair (LP) (N)) and an acceptor (lone vacancy (LV) (C)) and (right) a donor (LP (N)) and an acceptor (σ* (C-C)) NBOs (isosurface value = 0.03) of $H_3\mathbf{1}^{3+}$ with stabilizing energy of 60.2 and 7.2 kcal/mol, respectively [B3LYP-D3/def2tzvp for Pd, 6-311+G* for N, 6-311G* for other atoms in acetone (SMD model)//B3LYP-D3/def2svp for Pd, 6-31G* for other atoms].

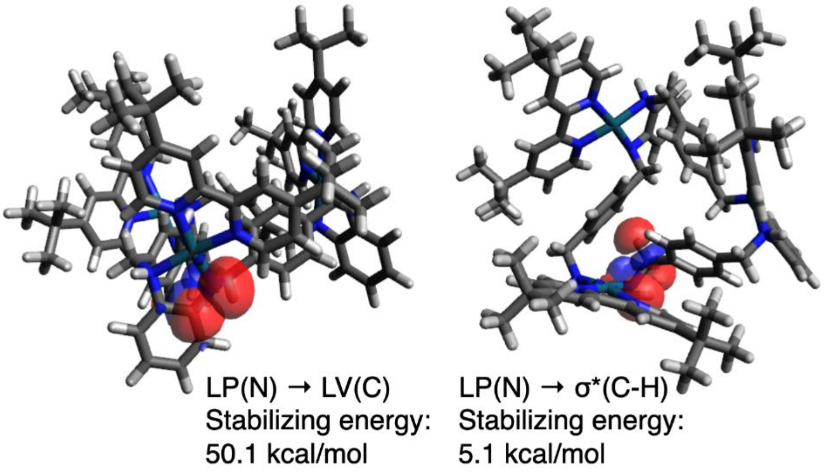

Figure S88. Superposition of (left) a donor (LP (N)) and an acceptor (LV (C)) and (right) a donor (LP (N)) and an acceptor (σ* (C-H)) NBOs (isosurface value = 0.03) of $H_3\mathbf{1}_{\mathbf{iso}}^{3+}$ with stabilizing energy of 50.1 and 5.1 kcal/mol, respectively [B3LYP-D3/def2tzvp for Pd, 6-311+G* for N, 6-311G* for other atoms in acetone (SMD model)//B3LYP-D3/def2svp for Pd, 6-31G* for other atoms].

5.5 DFT calculations of $[H_6\mathbf{1}\cdot OTf]^{5+}$

Geometry optimization, vibrational frequency and TD-DFT calculations of $[H_6\mathbf{1}\cdot OTf]^{5+}$ were also conducted to analyze the UV-vis spectrum of $H_6\mathbf{1}^{6+}$ and to compare the molecular orbitals and their energy levels with $H_3\mathbf{1}^{3+}$ under the same calculation conditions [B3LYP-D3/def2svp for Pd, 6-31G* for other atoms]. In the optimized structure, one TfO anion was located in the inner space surrounded

by three bipyridine moieties as observed in the crystal structure.[1] The TD-DFT calculations were performed in the gas phase, CH_3CN and acetone (SMD model).

The optimized structure of $[H_6\mathbf{1}\cdot OTf]^{5+}$ well agreed with the crystal structure as shown in Figure S86. Moreover, the UV-vis spectrum of $H_6\mathbf{1}^{6+}$ in CH_3CN was successfully reproduced by TD-DFT calculation of $[H_6\mathbf{1}\cdot OTf]^{5+}$ [B3LYP-D3/def2svp for Pd, 6-31G* for other atoms in CH_3CN (SMD)]. The calculated spectrum in acetone was not directly compared with the observed spectrum because the absorption in the UV region could not be measured due to the absorption of acetone but was similar to the observed spectrum in CH_3CN. These results suggested that the observed UV-vis spectra of $H_6\mathbf{1}^{6+}$ and $[H_{-3}\mathbf{1}]^{3+}$ were successfully reproduced by using the same functional and basis sets.

TD-DFT calculation of $[H_6\mathbf{1}\cdot OTf]^{5+}$ in CH_3CN suggested that the absorption around 310 nm observed in the CH_3CN solution is mainly assigned to π-π* transition of bipyridine moieties and interligand charge transfer from *para*-phenylene to bipyridine moieties. Comparing the transitions between $[H_6\mathbf{1}\cdot OTf]^{5+}$ and $H_3\mathbf{1}^{3+}$, the donor moieties were completely different. Especially, the absorption of $H_3\mathbf{1}^{3+}$ in the visible region is derived from the lone pair orbitals of amine moieties resulting from the deprotonation.

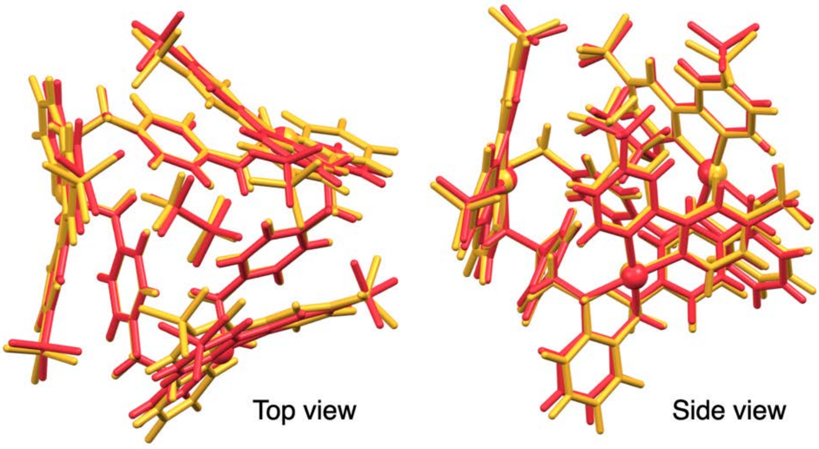

Figure S89. Overlaid view of (orange) the optimized structure of $[H_6\mathbf{1}\cdot OTf]^{5+}$ and (red) the crystal structure of $[H_6\mathbf{1}\cdot OTf]^{5+}$. Protons of *tert*-butyl groups are omitted for clarity.

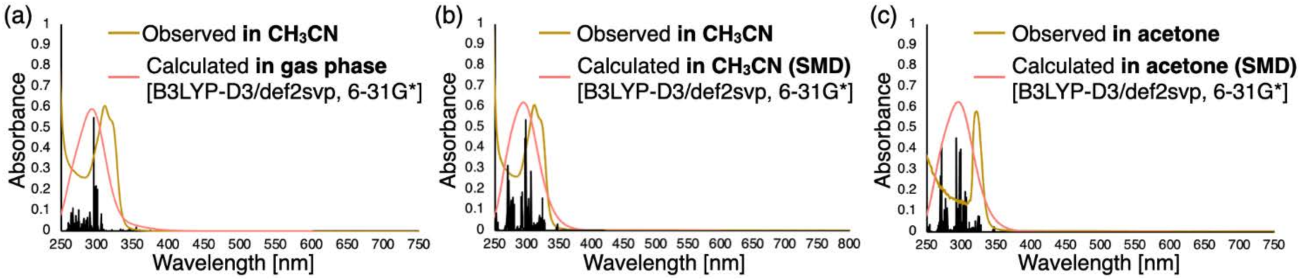

Figure S90. (dark yellow line) The observed UV-vis spectrum of $H_6\mathbf{1}^{6+}$ in CH_3CN and (pink and black lines) calculated UV-vis spectra of $[H_6\mathbf{1}\cdot OTf]^{5+}$ [B3LYP-D3/def2svp for Pd, 6-31G* for other atoms], (a) in gas phase, (b) in CH_3CN (SMD model) and (c) in acetone (SMD model).

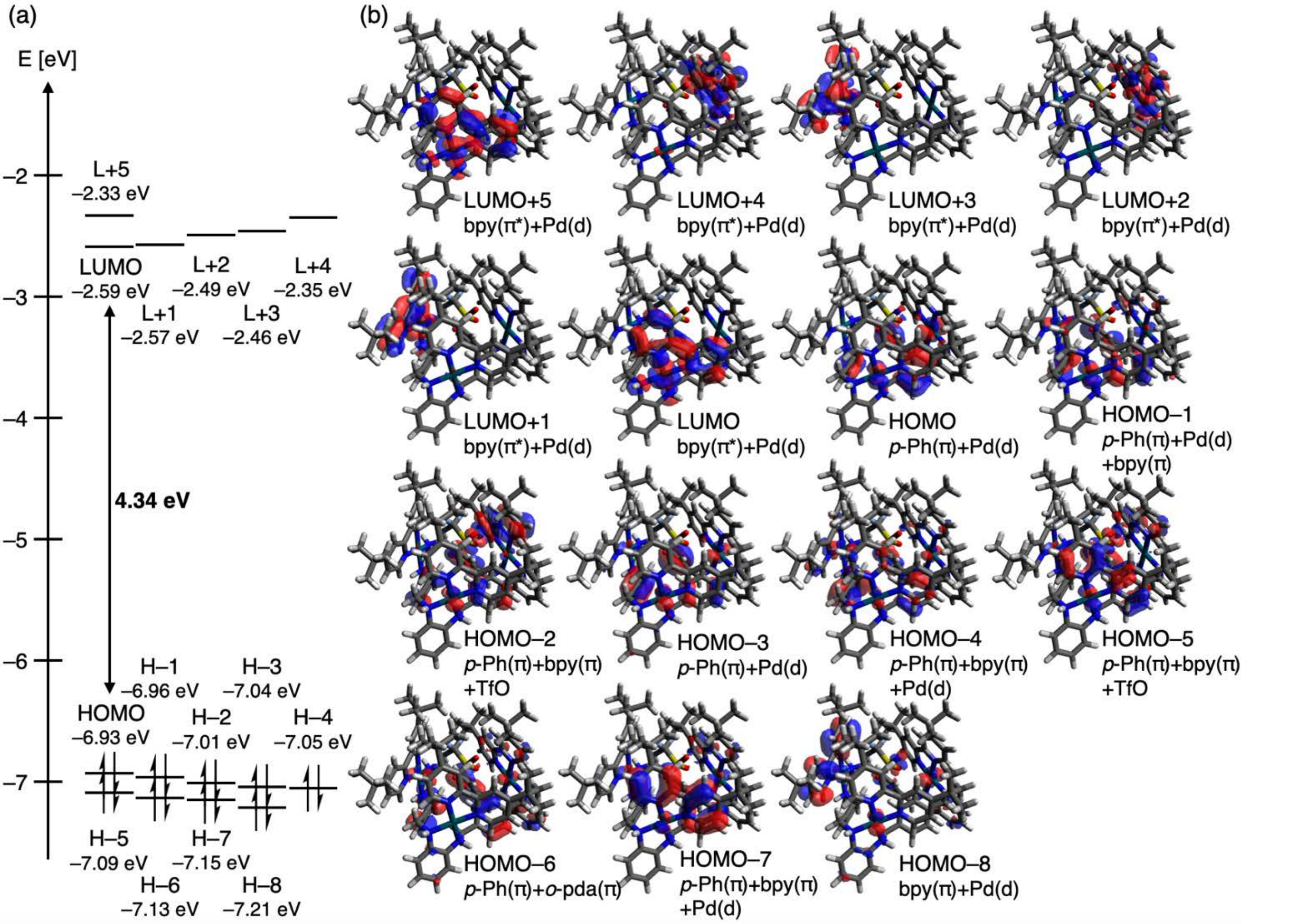
(a)
E [eV]
−2
−3
−4
−5
−6
−7
L+5
−2.33 eV
LUMO
−2.59 eV
L+1
−2.57 eV
L+2
−2.49 eV
L+3
−2.46 eV
L+4
−2.35 eV
4.34 eV
H−1
−6.96 eV
H−3
−7.04 eV
HOMO
−6.93 eV
H−2
−7.01 eV
H−4
−7.05 eV
H−5
−7.09 eV
H−7
−7.15 eV
H−6
−7.13 eV
H−8
−7.21 eV
(b)
LUMO+5
bpy(π*)+Pd(d)
LUMO+4
bpy(π*)+Pd(d)
LUMO+3
bpy(π*)+Pd(d)
LUMO+2
bpy(π*)+Pd(d)
LUMO+1
bpy(π*)+Pd(d)
LUMO
bpy(π*)+Pd(d)
HOMO
p-Ph(π)+Pd(d)
HOMO−1
p-Ph(π)+Pd(d)
+bpy(π)
HOMO−2
p-Ph(π)+bpy(π)
+TfO
HOMO−3
p-Ph(π)+Pd(d)
HOMO−4
p-Ph(π)+bpy(π)
+Pd(d)
HOMO−5
p-Ph(π)+bpy(π)
+TfO
HOMO−6
p-Ph(π)+o-pda(π)
HOMO−7
p-Ph(π)+bpy(π)
+Pd(d)
HOMO−8
bpy(π)+Pd(d)

(c)

State	ΔE [eV]	λ [nm]	f	Character
A	4.051	306.1	0.057	HOMO–2 -> LUMO+4 (31%), HOMO–1 -> LUMO+4 (21%), HOMO–2 -> LUMO+2 (10%)
A	4.158	298.2	0.107	HOMO–8 -> LUMO+3 (24%), HOMO–5 -> LUMO+1 (18%)
A	4.176	296.9	0.089	HOMO–7 -> LUMO+5 (16%), HOMO–5 -> LUMO+5 (11%)
A	4.179	296.7	0.057	HOMO–3 -> LUMO+4 (17%), HOMO–6 -> LUMO+4 (15%), HOMO–4 -> LUMO+4 (14%)

(d)

	LUMO+5	LUMO+4	LUMO+3	LUMO+2	LUMO+1	LUMO	HOMO	HOMO–1
Pd	27.0%	16.4%	30.2%	40.7%	26.7%	29.0%	25.8%	14.9%
o-pda	1.0%	0.6%	1.2%	1.7%	1.2%	1.2%	3.8%	5.3%
bpy	60.8%	75.0%	56.3%	41.3%	60.3%	57.1%	4.9%	13.8%
p-Ph	0.5%	0.5%	0.3%	0.3%	0.4%	0.4%	58.8%	60.4%
TfO⁻	0.1%	0.2%	0.0%	0.1%	0.0%	0.1%	2.2%	0.4%

	HOMO–2	HOMO–3	HOMO–4	HOMO–5	HOMO–6	HOMO–7	HOMO–8
Pd	6.4%	13.4%	13.1%	8.5%	6.8%	11.4%	12.6%
o-pda	1.3%	3.8%	1.7%	4.4%	17.5%	1.4%	9.0%
bpy	37.8%	7.7%	23.3%	31.5%	10.0%	64.5%	64.4%
p-Ph	39.9%	70.3%	57.7%	37.8%	60.9%	19.4%	5.6%
TfO⁻	11.9%	0.3%	0.3%	14.4%	0.6%	0.7%	5.5%

Figure S91. (a) The orbital energy level of $[H_6\mathbf{1}\cdot OTf]^{5+}$ based on DFT calculation in CH_3CN (SMD model) [B3LYP-D3/def2svp for Pd, 6-31G* for other atoms], (b) calculated frontier orbitals of $[H_6\mathbf{1}\cdot OTf]^{5+}$ (isosurface value = 0.025), (c) assignment of excited states with transition energy (Δ*E*), wavelength (*λ*) and oscillator strength (*f*) calculated in CH_3CN (SMD model) in Figure S90 (b) and (d) Mulliken population decomposition analysis of LUMO+5 to HOMO–8 of $[H_6\mathbf{1}\cdot OTf]^{5+}$ in CH_3CN (SMD model). Pd, *o*-pda, bpy, *p*-Ph and TfO^- indicate Pd atoms, *ortho*-phenylenediamine, bipyridine, *para*-phenylene moieties and triflate anion, respectively.

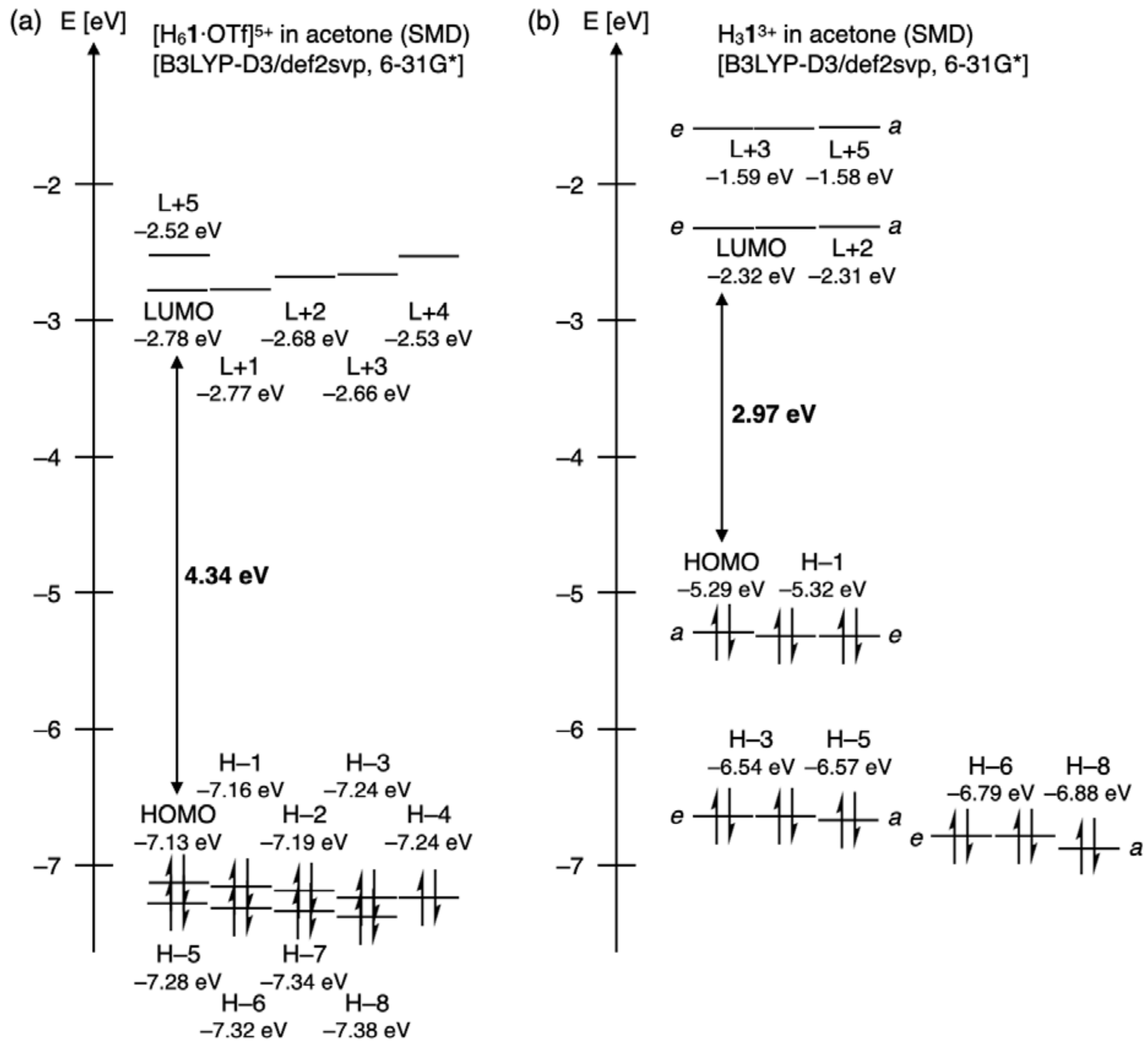

Figure S92. The orbital energy level of (a) $[H_6\mathbf{1}\cdot OTf]^{5+}$ and (b) $H_3\mathbf{1}^{3+}$ based on DFT calculation in acetone (SMD model) [B3LYP-D3/def2svp for Pd, 6-31G* for other atoms].

5.6 DFT calculations of 1

Geometry optimization, vibrational frequency and TD-DFT calculations of **1** were also conducted to support the formation of **1** and analyze the UV-vis spectrum under the same calculation conditions [B3LYP-D3/def2svp for Pd, 6-31G* for other atoms]. The TD-DFT calculation was performed in THF (SMD model).

The observed UV-vis spectrum was reproduced by the TD-DFT calculation, and the two characteristics absorptions were successfully assigned. The absorptions around 870 and 510 nm were assigned to ILCT from the lone pair orbitals (HOMO–HOMO–2) of the deprotonated amine nitrogen moieties to the π* orbitals (LUMO–LUMO+2) of the bipyridine moieties. Compared with $[H_6\mathbf{1}\cdot OTf]^{5+}$ and $H_3\mathbf{1}^{3+}$, the HOMO energy level was significantly destabilized by the deprotonation using ^{t}BuOK, resulting in the significant red-shift of the absorption to the near IR region. The experimental and

calculation results supported the formation fully deprotonated trinuclear Pd^{II} complex **1** by the reaction with ^{t}BuOK. The simulated spectrum is also consistent with the formation of other possible products such as H_2**1**$^{2+}$ and H**1**$^+$ having one or two di-deprotonated *o*-phenylenediamine moieties.

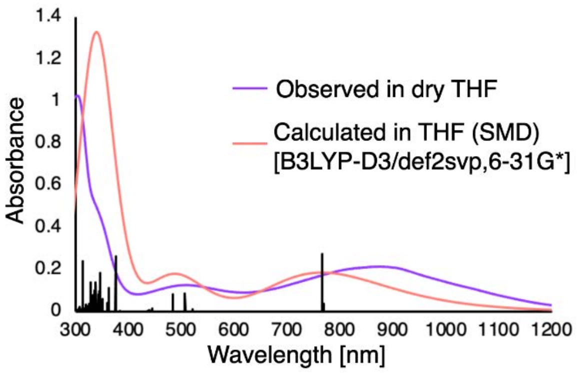

Figure S93. (purple line) The observed UV-vis spectrum of **1** in THF and (pink and black lines) calculated UV-vis spectra of **1** [B3LYP-D3/def2svp for Pd, 6-31G* for other atoms] in THF (SMD model).

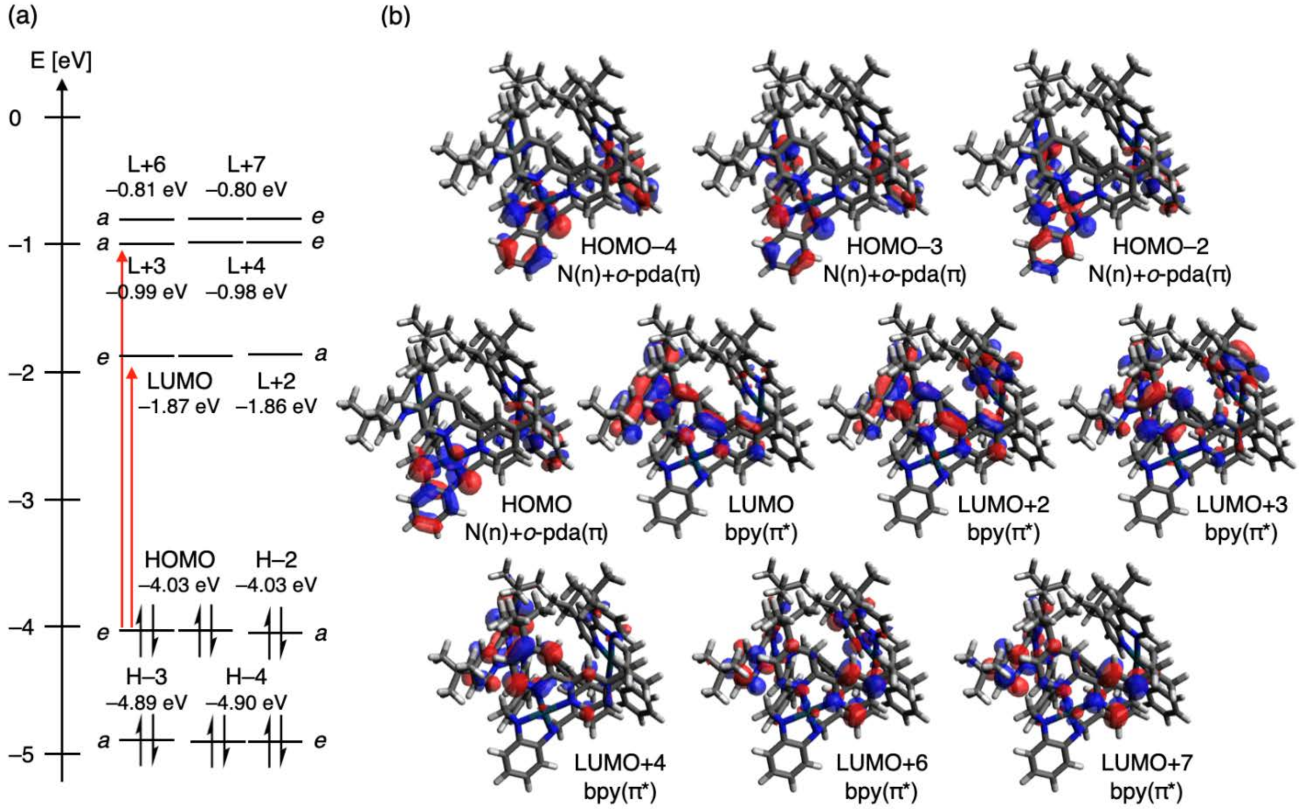

(c)

State	ΔE [eV]	λ [nm]	f	Character
E	1.614	768.4	0.024	HOMO(*e*)->LUMO(*e*) (38%), HOMO(*e*)->LUMO+2(*a*) (30%), HOMO–2(*a*)->LUMO(*e*) (30%)
A	1.620	765.3	0.091	HOMO(*e*)->LUMO(*e*) (72%), HOMO–2(*a*)->LUMO+2(*a*) (27%)
A	2.447	506.6	0.029	HOMO(*e*)->LUMO+4(*e*) (28%), HOMO–4(*e*)->LUMO(*e*) (20%), HOMO–3(*a*)->LUMO+2(*a*) (13%), HOMO–2(*a*)->LUMO+3(*a*) (13%)
E	2.567	483.1	0.054	HOMO(*e*)->LUMO+6(*a*) (16%), HOMO(*e*)->LUMO+7(*e*) (16%), HOMO–2(*a*)->LUMO+7(*e*) (14%), HOMO(*e*)->LUMO+3(*a*) (10%), HOMO(*e*)->LUMO+4(*e*) (10%)

(d)

	HOMO–3	HOMO	LUMO	LUMO+3	LUMO+6
Pd	14.9%	11.2%	3.2%	2.3%	3.3%
N(n)	45.5%	48.4%	1.3%	1.0%	1.6%
o-pda	26.4%	28.4%	0.2%	0.2%	0.2%
bpy	3.7%	3.0%	92.3%	91.8%	88.5%

Figure S94. (a) The orbital energy level of **1** based on DFT calculation in THF (SMD model) [B3LYP-D3/def2svp for Pd, 6-31G* for other atoms], (b) calculated frontier orbitals of **1** (isosurface value = 0.025), (c) assignment of excited states with transition energy (Δ*E*), wavelength (*λ*) and oscillator strength (*f*) calculated in THF (SMD model) in Figure S88 (b) and (d) Mulliken population decomposition analysis of LUMO+6, LUMO+3, LUMO, HOMO and HOMO–3 of **1** in THF (SMD model). Pd, N(n), *o*-pda and bpy indicate Pd atoms, lone pairs of amine nitrogen atoms, *ortho*-phenylenediamine and bipyridine moieties, respectively.

6. References

1. Nakajima, T.; Tashiro, S.; Ehara, M.; Shionoya M. Selective synthesis of tightly- and loosely-twisted metallomacrocycle isomers towards precise control of helicity inversion motion. *Nat. Commun.* **2023**, *14*, 7868. DOI: https://doi.org/10.1038/s41467-023-43658-5
2. Hayashi, R.; Tashiro, S.; Asakura, M.; Mitsui, S.; Shionoya, M. Effector-dependent structural transformation of a crystalline framework with allosteric effects on molecular recognition ability. *Nat. Commun*. **2023**, *14*, 4490. DOI: https://doi.org/10.1038/s41467-023-40091-6
3. Dolomanov, O.V.; Bourhis, L. J.; Gildea, R. J.; Howard, J. A. K.; Puschmann, H. *OLEX2*: a complete structure solution, refinement and analysis program. *J. Appl. Cryst.* **2009**, *42*, 339–341. DOI: https://doi.org/10.1107/S0021889808042726
4. Sheldrick, G. M. *SHELXL-97: Program for refinement of crystal structure.* University of Göttingen, Göttingen, Germany (1997); Sheldrick, G. M. *SHELXL-2013*. University of Göttingen, Göttingen, Germany (2013).
5. Perrin, C. L. & Dwyer, T. J. Application of two-dimensional NMR to kinetics of chemical exchange. *Chem. Rev.* **1990**, *90*, 935–967. DOI: https://doi.org/10.1021/cr00104a002
6. StatPlus:mac, AnalystSoft Inc. – statistical analysis program for macOS. Version v8.
7. Gaussian 16, Revision C.01, Frisch, M. J.; Trucks, G. W.; Schlegel, H. B.; Scuseria, G. E.; Robb, M. A.; Cheeseman, J. R.; Scalmani, G.; Barone, V.; Petersson, G. A.; Nakatsuji, H.; Li, X.; Caricato, M.; Marenich, A. V.; Bloino, J.; Janesko, B. G.; Gomperts, R.; Mennucci, B.; Hratchian, H. P.; Ortiz, J. V.; Izmaylov, A. F.; Sonnenberg, J. L.; Williams-Young, D.; Ding, F.; Lipparini, F.; Egidi, F.; Goings, J.; Peng, B.; Petrone, A.; Henderson, T.; Ranasinghe, D.; Zakrzewski, V. G.; Gao, J.; Rega, N.; Zheng, G.; Liang, W.; Hada, M.; Ehara, M.; Toyota, K.; Fukuda, R.; Hasegawa, J.; Ishida, M.; Nakajima, T.; Honda, Y.; Kitao, O.; Nakai, H.; Vreven, T.; Throssell, K.; Montgomery, Jr. J. A.; Peralta, J. E.; Ogliaro, F.; Bearpark, M. J.; Heyd, J. J.; Brothers, E. N.; Kudin, K. N.; Staroverov, V. N.; Keith, T. A.; Kobayashi, R.; Normand, J.; Raghavachari, K.; Rendell, A. P.; Burant, J. C.; Iyengar, S. S.; Tomasi, J.; Cossi, M.; Millam, J. M.; Klene, M.; Adamo, C.; Cammi, R.; Ochterski, J. W.; Martin, R. L.; Morokuma, K.; Farkas, O.; Foresman, J. B.; Fox, D. J. Gaussian, Inc., Wallingford CT (2019).
8. Becke, A. D. Density-functional thermochemistry. III. The role of exact exchange. *J. Chem. Phys.* **1993**, *98*, 5648–5652. DOI: https://doi.org/10.1063/1.464913
9. Stephens, P. J.; Devlin, F. J.; Chabalowski, C. F.; Frisch, M. J. *Ab initio* calculation of vibrational absorption and circular dichroism spectra using density functional force field. *J. Chem. Phys.* **1994**, *98*, 11623–11627. DOI: https://doi.org/10.1021/j100096a001
10. Grimme, S.; Antony, J.; Ehrlich, S.; Krieg, H. A consistent and accurate *ab initio* parametrization of density functional dispersion correction (DFT-D) for the 94 elements H-Pu. *J. Chem. Phys.* **2010**, *132*, 154104. DOI: https://doi.org/10.1063/1.3382344

11. Weigend, F.; Ahlrichs, R. Balanced basis sets of split valence, triple zeta valence and quadruple zeta valence quality for H to Rn: design and assessment of accuracy. *Phys. Chem. Chem. Phys.* **2005**, *7*, 3297–3305. DOI: https://doi.org/10.1039/B508541A
12. Hehre, W. J.; Ditchfield, R.; Pople, J. A. Self-consistent molecular orbital methods. XII. Further extensions of Gaussian-type basis sets for use in molecular orbital studies of organic molecules. *J. Chem. Phys.* **1972**, *56*, 2257–2261. DOI: https://doi.org/10.1063/1.1677527
13. Hariharan, P. C.; Pople, J. A. The influence of polarization functions on molecular orbital hydrogenation energies. *Theoret. Chim. Acta* **1973**, *28*, 213–222. DOI: https://doi.org/10.1007/BF00533485
14. Krishnan, R.; Binkley, J. S.; Seeger, R.; Pople, J. A. Self-consistent molecular orbital methods. XX. A basis set for correlated wave functions. *J. Chem. Phys.* **1980**, *72*, 650–654. DOI: https://doi.org/10.1063/1.438955
15. Clark, T.; Chandrasekhar, J.; Spitznagel, G. W.; Schleyer, P. v. R. Efficient diffuse function-augmented basis sets for anion calculations. III. The 3-21+G basis set for first-row elements, Li-F. *J. Comput. Chem.* **1983**, *4*, 294–301. DOI: https://doi.org/10.1002/jcc.540040303
16. O'Boyle, N. M.; Tenderholt, A. L.; Langner, K. M. Cclib: a library for package-independent computational chemistry algorisms. *J. Comput. Chem.* **2008**, *29*, 839–845. DOI: https://doi.org/10.1002/jcc.20823
17. Marenich, A. V.; Cramer, C. J.; Truhlar, D. G. Universal solvation model based on solute electron density and on a continuum model of the solvent defined by the bulk dielectric constant and atomic surface tensions. *J. Phys. Chem. B* **2009**, *113*, 6378–6396. DOI: https://doi.org/10.1021/jp810292n
18. Lu, T.; Chen, F. A multifunctional wavefunction analyzer. *J. Comput. Chem.* **2012**, *33*, 580–592. DOI: https://doi.org/10.1002/jcc.22885
19. Hanwell, M. D.; Curtis, D. E.; Lonie, D. C.; Vandermeersch, T.; Zurek, E.; Hutchison, G. R. Avogadro: an advanced semantic chemical editor, visualization, and analysis platform. *J. Cheminf.* **2012**, *4*, 17. DOI: https://doi.org/10.1186/1758-2946-4-17
20. NBO 7.0. Glendening, E. D.; Badenhoop, J. K.; Reed, A. E.; Carpenter, J. E.; Bohmann, J. A.; Morales, C. M.; Karafiloglou, P.; Landis, C. R.; Weinhold, F. Theoretical Chemistry Institute, University of Wisconsin, Madison, WI (2018).
21. Yanai, T.; Tew, D. P.; Handy, N. C. A new hybrid exchange-correlation functional using the Coulomb-attenuating method (CAM-B3LYP). *Chem. Phys. Lett.* **2004**, *393*, 51–57. DOI: https://doi.org/10.1016/j.cplett.2004.06.011
22. Zhao, Y.; Truhlar, D. G. The M06 suite of density functionals for main group thermochemistry, thermochemical kinetics, noncovalent interactions, excited states, and transition elements: two new functionals and systematic testing of four M06-class functionals and 12 other functionals. *Theor. Chem. Acc.* **2008**, *120*, 215–241. DOI: https://doi.org/10.1007/s00214-007-0310-x
23. Yu, H. S.; Li, S. L.; Truhlar, D. G. MN15: A Kohn-Sham global-hybrid exchange-correlation density functional with broad accuracy for multi-reference and single-reference systems and

noncovalent interactions. *Chem. Sci.* **2016**, *7*, 5032–5051. DOI: https://doi.org/10.1039/C6SC00705H

24. Chai, J.-D. & Head-Gordon M. Systematic optimization of long-range corrected hybrid density functionals. *J. Chem. Phys.* **2008**, *128*, 084106. DOI: https://doi.org/10.1063/1.2834918
